# Achieving coordinated national immunity and cholera elimination in Haiti through vaccination: a modelling study

**DOI:** 10.1016/S2214-109X(20)30310-7

**Published:** 2020-07-22

**Authors:** Elizabeth C Lee, Dennis L Chao, Joseph C Lemaitre, Laura Matrajt, Damiano Pasetto, Javier Perez-Saez, Flavio Finger, Andrea Rinaldo, Jonathan D Sugimoto, M Elizabeth Halloran, Ira M Longini, Ralph Ternier, Kenia Vissieres, Andrew S Azman, Justin Lessler, Louise C Ivers

**Affiliations:** a Department of Epidemiology, Johns Hopkins Bloomberg School of Public Health, Baltimore, MD, USA; b Institute for Disease Modeling, Bellevue, WA, USA; c Laboratory of Ecohydrology, School of Architecture, Civil and Environmental Engineering, École Polytechnique Fédérale de Lausanne, Lausanne, Switzerland; d Vaccine and Infectious Disease Division, Fred Hutchinson Cancer Research Center, Seattle, WA, USA; e Department of Environmental Sciences, Informatics and Statistics, Ca' Foscari University of Venice, Venice, Italy; f Centre for Mathematical Modelling of Infectious Diseases and Department for Infectious Disease Epidemiology, Faculty of Epidemiology and Population Health, London School of Hygiene & Tropical Medicine, London, UK; g Department of Biostatistics, University of Washington, Seattle, WA, USA; h Department of Biostatistics, College of Public Health and Health Professions, and Emerging Pathogens Institute, University of Florida, Gainesville, FL, USA; i Partners In Health/Zanmi Lasante, Port-au-Prince, Haiti; j Department of Global Health and Social Medicine, Harvard Medical School, Boston, MA, USA; k Center for Global Health, Massachusetts General Hospital, Boston, MA, USA

## Abstract

**Background:**

Cholera was introduced into Haiti in 2010. Since then, more than 820 000 cases and nearly 10 000 deaths have been reported. Oral cholera vaccine (OCV) is safe and effective, but has not been seen as a primary tool for cholera elimination due to a limited period of protection and constrained supplies. Regionally, epidemic cholera is contained to the island of Hispaniola, and the lowest numbers of cases since the epidemic began were reported in 2019. Hence, Haiti may represent a unique opportunity to eliminate cholera with OCV.

**Methods:**

In this modelling study, we assessed the probability of elimination, time to elimination, and percentage of cases averted with OCV campaign scenarios in Haiti through simulations from four modelling teams. For a 10-year period from January 19, 2019, to Jan 13, 2029, we compared a no vaccination scenario with five OCV campaign scenarios that differed in geographical scope, coverage, and rollout duration. Teams used weekly department-level reports of suspected cholera cases from the Haiti Ministry of Public Health and Population to calibrate the models and used common vaccine-related assumptions, but other model features were determined independently.

**Findings:**

Among campaigns with the same vaccination coverage (70% fully vaccinated), the median probability of elimination after 5 years was 0–18% for no vaccination, 0–33% for 2-year campaigns focused in the two departments with the highest historical incidence, 0–72% for three-department campaigns, and 35–100% for nationwide campaigns. Two-department campaigns averted a median of 12–58% of infections, three-department campaigns averted 29–80% of infections, and national campaigns averted 58–95% of infections. Extending the national campaign to a 5-year rollout (compared to a 2-year rollout), reduced the probability of elimination to 0–95% and the proportion of cases averted to 37–86%.

**Interpretation:**

Models suggest that the probability of achieving zero transmission of *Vibrio cholerae* in Haiti with current methods of control is low, and that bolder action is needed to promote elimination of cholera from the region. Large-scale cholera vaccination campaigns in Haiti would offer the opportunity to synchronise nationwide immunity, providing near-term population protection while improvements to water and sanitation promote long-term cholera elimination.

**Funding:**

Bill & Melinda Gates Foundation, Global Good Fund, Institute for Disease Modeling, Swiss National Science Foundation, and US National Institutes of Health.

## Introduction

On Jan 12, 2010, a massive earthquake struck Haiti, displacing more than 1 million people and further disrupting the already inadequate water and sanitation infrastructure.^1^ In October, 2010, pandemic *Vibrio cholerae* O1 was introduced into Haiti for the first time by soldiers from the UN Stabilization Mission, who were themselves using deficient sanitation facilities.^2^ This initiated one of the largest cholera outbreaks in the modern era, resulting in more than 600 000 reported cases and more than 7000 reported deaths in the first 2 years.^3^ Subsequent studies suggest that more deaths might have occurred, especially in rural communities with limited access to health services and poor disease surveillance.^4^ Cholera has since become endemic in the country, resulting in more than 820 000 reported cases and nearly 10 000 deaths as of Jan 18, 2020. Although incidence rates have declined substantially in the past few years, in 2018, more than 3700 cholera cases and 41 deaths were reported across nine of the ten departments (first-level administrative units) in Haiti (no cases were reported in the tenth department).^5^

Killed oral cholera vaccine (OCV) has become accepted as a safe and effective tool for cholera prevention and control. The standard two-dose course is 76% (95% CI 62–85) effective against clinical disease.^6^ However, protection from the vaccine wanes over time, and OCV is far less effective in young children than in adults (vaccine efficacy is 30% in children younger than 5 years).^6^ Further, despite prequalification of the vaccine by WHO in 2011 and establishment of the global OCV stockpile in 2013, vaccine supply is constrained by a limited global manufacturing capacity. Approximately 23 million doses were delivered per year in 2018 and 2019 to serve the estimated 1·3 billion people who are at risk worldwide,^7^, ^8^ and requests from the OCV stockpile exceeded annual production. For these reasons, OCV is not generally considered a practical tool for sustained cholera elimination, since herd immunity would be difficult to achieve and maintain.

**Research in context**

**Evidence before this study**

We searched PubMed without language or date restrictions on Oct 4, 2019, for all records matching (“cholera*” AND “Haiti” AND (“vaccin*” OR “elim*”)) in any field and added one known article on the probability of elimination of cholera that was not indexed by PubMed to our review. Of 94 results, four articles were not about the cholera outbreak in Haiti or the use of cholera vaccination and 34 were not original research articles. 14 articles presented research on cholera biology or cholera vaccine biology, through discussion of *Vibrio cholerae* genetics, immunogenicity of oral cholera vaccine (OCV), or prospective vaccine candidate antigens. 20 articles assessed the effectiveness of OCV, evaluated OCV campaign implementation or attitudes and knowledge about cholera control, or presented lessons learned about outbreak response and policy as a result of the cholera outbreak in Haiti. Seven articles were about general cholera outbreak epidemiology in Haiti, and six articles were broadly related to cholera transmission modelling but not in Haiti or were not about the impact of vaccination. Of the nine remaining articles, five examined the impact of potential OCV campaigns at an early timepoint (2010–11), when the cholera outbreak in Haiti still exhibited epidemic dynamics, and one projected the impact of OCV campaigns planned after Hurricane Matthew in 2016. Two of the articles considered prospects for cholera elimination in Haiti in 2013 and 2014 and found that further targeted interventions were needed. One study from 2017 modelled the potential for OCV campaigns to eliminate cholera transmission in the Ouest department.

**Added value of this study**

Although the lowest number of cholera cases in Haiti since the outbreak began was reported in 2019, model simulations suggest that *V cholerae* transmission could persist without additional cholera control interventions. We found that, although a single two-department vaccination campaign may avert roughly 13–58% of *V cholerae* infections over a 5-year period, only a nationwide campaign led to a high probability of cholera elimination in our modelling simulations. Previous assessments of the impact of OCV use in Haiti were made early during the outbreak when OCV campaigns were unlikely to lead to cholera elimination. Our study projects cholera transmission in Haiti with multiple years of more recent data, and directly examines the prospect of cholera elimination without an OCV vaccination campaign (that is, maintaining the status quo) and under various mass OCV campaign scenarios. In bringing together results from four modelling teams, our study provides robust evidence about the current state of cholera transmission across Haiti and the potential impact of several mass OCV campaign scenarios.

**Implications of all the available evidence**

Although cholera elimination might be possible without large-scale vaccine or water and sanitation interventions, this work provides strong support that ambitious nationwide vaccination campaigns could break the cycle of endemic cholera transmission in Haiti as long-term improvements to water and sanitation infrastructure are made.

However, the situation in Haiti could present a unique opportunity to eliminate cholera from a region through the use of vaccine as a complement to investment in long-term water, sanitation, and hygiene infrastructure. Outside Haiti, the Americas are largely free from sustained cholera transmission. Only the Dominican Republic, which shares the island of Hispaniola with Haiti, has reported active transmission, but at far lower rates (2800 suspected cases annually in 2014–17 *vs* 58 700 in Haiti), and the last reported cases were in 2018.^9^, ^10^, ^11^, ^12^, ^13^, ^14^, ^15^, ^16^, ^17^ Further, Haiti is a fairly small country (<13 million people) compared with other countries with endemic cholera such as the Democratic Republic of the Congo (85 million people) and Bangladesh (160 million people). If mass vaccination could achieve coordinated immunity to cholera throughout Haiti, and this immunity could be maintained for a long enough period to clear cholera from local water supplies, the country would become cholera free, and would probably remain so because of the low historical probability of introduction.^1^

Several previous studies have simulated the impact of cholera control interventions in Haiti. Two studies compared the effects of OCV campaigns and water, sanitation, and hygiene improvements.^18^, ^19^ However, most examined effects only within the first few years of the outbreak (when epidemic cholera dynamics prevailed),^18^, ^19^, ^20^, ^21^ did not perform long-term projections,^19^, ^21^, ^22^ focused on a single department,^23^ or analysed historical OCV campaigns.^20^, ^24^

In this study, we assessed the impact of five prospective OCV campaign scenarios compared with a status quo scenario (no vaccination) for a projected 10-year period in Haiti. Four independent modelling teams expanded on previously developed models of cholera transmission and vaccination interventions in Haiti^21^, ^22^, ^25^, ^26^ to simulate the effects of mass vaccination campaigns of varying geographical scope, vaccination coverage, and rollout duration to assess the probability of elimination, time to elimination, and percentage of cases averted in each scenario. The aim of these analyses was to determine the feasibility of cholera elimination in Haiti if the status quo (which includes regional and local cholera control measures but no vaccination) were maintained and through OCV use alone, and to inform ongoing policy discussions about the scope and rollout of potential OCV campaigns in Haiti in the near future.

## Methods

### Study overview

In this modelling study, we examined the health impact and feasibility of cholera elimination in Haiti with mass vaccination campaigns by establishing a consortium of four independent research teams that had previously modelled cholera transmission in Haiti. Teams fit their models to a common cholera incidence data source and generated model projections of true and reported cholera incidence for 10 years beyond the end of the data available for model fitting. Six projections were produced by each model: a status quo (no vaccination) scenario and five vaccination campaign scenarios that differed by deployment and vaccination coverage. Teams were asked to estimate the probability of cholera elimination, time to cholera elimination, and the health impact of mass OCV campaigns for each scenario.

### Project coordination

We discussed the goals of the project and methods with partners in the Haiti Ministry of Public Health and Population at the onset of this initiative for feedback on the approach and primary assumptions. Once work had started, we had several consultative meetings in Haiti and by teleconference with epidemiologists, researchers, and clinicians who were involved in the cholera response in Haiti in their individual capacities.

For ease of comparison, the consortium decided on common parameters and assumptions related to vaccine protection and vaccine campaign logistics (detailed in the following sections). We also shared common data sources, used common definitions of disease elimination, and produced comparable outputs for figures and analyses. All other modelling decisions and assumptions were left to the discretion of each team, as described in appendix 3. The final models underwent internal review from at least one other team.

### Data sources

**Figure 1:**
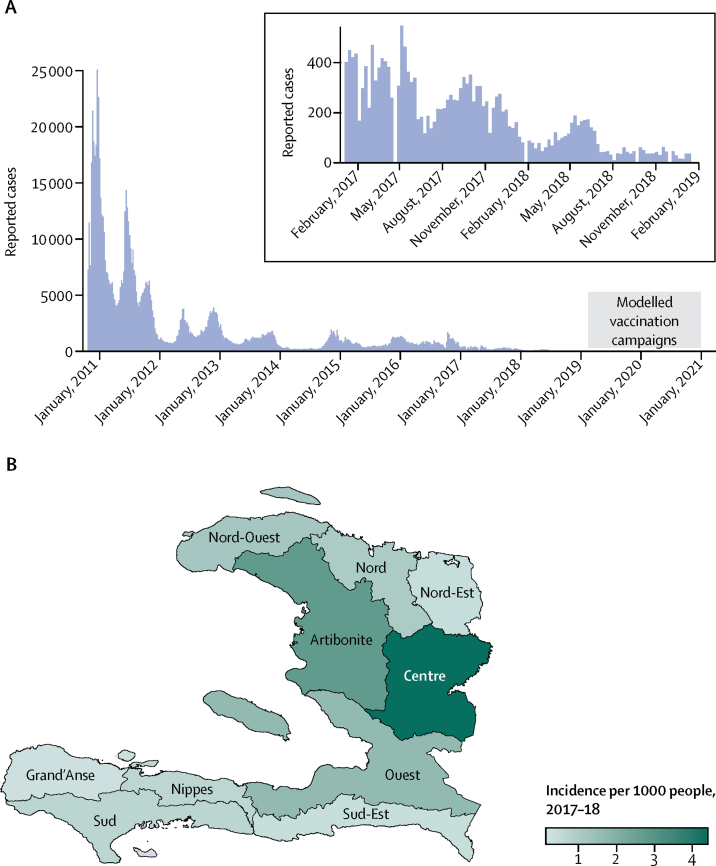
Historical cholera incidence in Haiti (A) Weekly reports of cholera to the Ministère de la Santé Publique et de la Population in Haiti from October, 2010, to January, 2019, with an inset of the period after January, 2017. The grey box represents the 2-year vaccination campaign period in the modelling exercise. (B) Map of reported cholera incidence per 1000 people from 2017 to 2018 across Haitian departments.

All teams calibrated their models to publicly available weekly department-level reports of suspected cholera cases from the Haiti Ministry of Public Health and Population website.^5^ Data were available for the epidemiological week ending on Oct 23, 2010, to the week ending on Jan 12, 2019 (figure 1). Teams also had access to optional shared data sources on diagnostic testing and previous OCV campaigns, which were used to calibrate or validate the models (appendix 3 pp 3–4).

### Epidemiological modelling

The models ranged from simple stochastic compartmental models to agent-based models of cholera dynamics in the entire country (table). Model 1 (Johns Hopkins Bloomberg School of Public Health, Baltimore, MD, USA) represented all of Haiti as a single population in a stochastic compartmental model. Model 2 (Fred Hutchinson Cancer Research Center, Seattle, WA, USA; and University of Florida, Gainesville, FL, USA) was a deterministic metapopulation model and Model 3 (École Polytechnique Fédérale de Lausanne, Lausanne, Switzerland) was a stochastic metapopulation model, each of which had an independent approach to modelling interdepartmental connectivity and the dynamics of cholera reservoirs. Model 4 (Institute for Disease Modeling, Bellevue, WA, USA) was an agent-based model that used a synthetic representation of the Haitian population, and its household structure, connectivity, and interaction with aquatic reservoirs. Supplementary methods, results, and model code are available from all modelling teams in appendix 3 (p 12–16) and online.

**Table:** Summary of key model features across teams

	**Spatial scale**	**Seasonality function**	**Environmental transmission**	**Age structure**	**Spatial transmission dynamics**
Model 1^22^	National	Basis splines	None	None	None
Model 2	Department	Sinusoidal	Yes	None	Road and river networks
Model 3^24^, ^25^, ^26^, ^27^	Department	Rainfall-driven	Yes	None	Calibrated human mobility
Model 4^21^	1 km × 1 km grid	Rainfall-driven	Yes	Yes	Road and river networks, commuting

**Figure 2:**
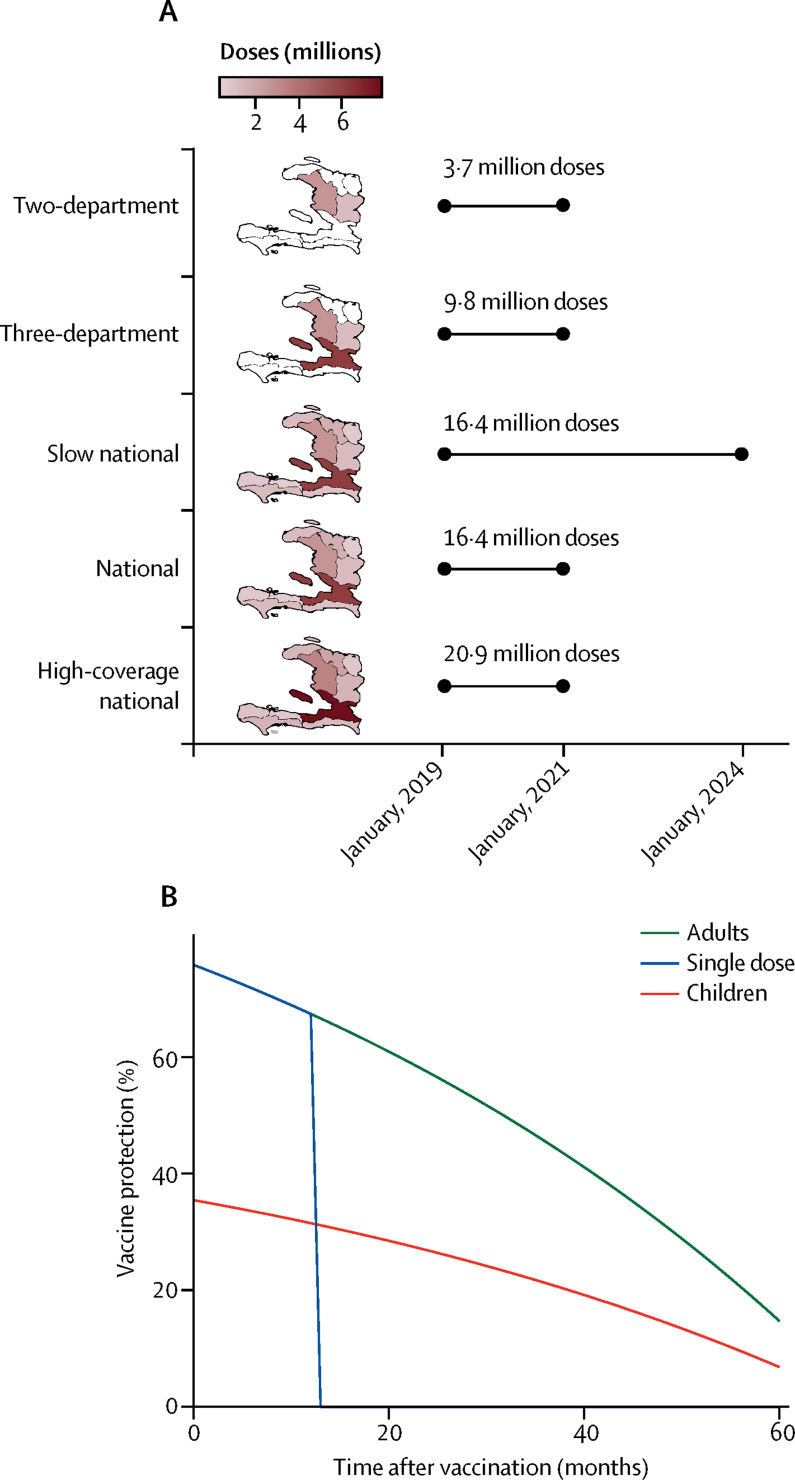
Vaccination campaign scenarios and vaccine protection assumptions (A) Summary of the geographical scope, vaccination campaign deployment, and total number of oral cholera vaccine (OCV) doses needed for each of the five modelled vaccination campaign scenarios. Departments in white did not receive any OCV doses in a given campaign. (B) Vaccine protection assumptions for two doses administered to adults, two doses administered to children, and one dose administered to adults.

Teams simulated six scenarios that used different combinations of parameters for vaccine campaign logistics and vaccination coverage (figure 2). These strategies included four vaccine campaign deployment strategies, each starting the day or week (depending on model implementation) after the last data point used for model calibration (week ending Jan 12, 2019). Campaigns targeted departments in order of highest to lowest 2017–18 cumulative incidence (figure 1, appendix 3 p 5) and distributed 4–21 million doses across different scenarios (appendix 3 p 5).

The six scenarios were as follows: maintaining the status quo (no vaccination); an OCV campaign over 2 years with baseline vaccination coverage, limited to the two departments most affected by cholera (Centre and Artibonite), similar to the national cholera elimination plan for Haiti^28^ (two-department); an OCV campaign over 2 years with baseline vaccination coverage, limited to the departments of Centre, Artibonite, and Ouest (which includes the populous capital Port-au-Prince; three-department); a national OCV campaign over 2 years with baseline vaccination coverage (national); a national OCV campaign over 5 years with baseline vaccination coverage (slow national); and a national OCV campaign over 2 years with high vaccination coverage (high-coverage national).

All four models included a seasonally varying transmission parameter, with some driven by rainfall and others making no mechanistic assumptions (table). Models 1, 2, and 3 had two calibration periods (for periods of epidemic and endemic dynamics), and simulated future scenarios based on parameters fit to the more recent calibration period. Concomitant non-vaccine interventions and changes to cholera risk factors such as access to improved water and sanitation were not explicitly modelled. All models assumed that external conditions except those related to vaccination campaigns remained constant during the projection period.

### Vaccination coverage

Killed OCVs are licensed as a two-dose regimen, with doses taken at least 2 weeks apart.^6^ Our baseline scenario assumes that vaccine coverage is the same in all departments, with 70% two-dose coverage, 10% one-dose coverage, and 20% receiving no vaccine. In the high-coverage campaign, departments were assumed to achieve 95% two-dose coverage and 1·67% one-dose only coverage, with 3·33% receiving no vaccine by the end of the campaign.

All teams assumed that initial vaccine protection was 76%, as estimated by a recent case-control study in Haiti.^29^ We estimated waning vaccine protection for 60 months after vaccination by fitting a log-linear weighted regression model to the raw data from a published meta-analysis on killed OCV efficacy against medically attended culture-confirmed cholera (figure 2, appendix 3 p 6).^6^ To be conservative, we assumed that the vaccine provided no protection after the end of 5 years.

The four models made different assumptions about the types of protection provided by the vaccine. In Model 1, vaccination reduced the probability of clinical disease and individual infectiousness. In Model 2, Model 3, and Model 4, vaccination reduced susceptibility to infection.

According to estimates from a recent meta-analysis, in children aged less than 5 years OCV is on average 46·9% as effective as in adults.^6^ As there are limited data on vaccine efficacy and effectiveness among children, we used this conservative multiplier to adjust the adult vaccine protection for children younger than 5 years (figure 2, appendix 3 p 6). Children aged 5 years or older were assumed to have the same protection as adults.

In the first year after vaccination, individuals who received a single vaccine dose were assumed to have the same protection as those with two doses, after which the single-dose protection dropped to zero, similar to results from a case-control study in Haiti (figure 2, appendix 3 p 6).^30^, ^31^

### Model outcomes

Each team estimated the probability of elimination within 10 years after the start of each vaccination campaign, which was defined as the proportion of simulations that achieved less than one infection with *V cholerae* (including reported and unreported infections) over at least 52 consecutive weeks. Within the context of our modelling exercise, a 10-year period without resurgence was deemed adequate to represent true elimination of disease transmission (ie, *V cholerae* would be unlikely to be reseeded in the population from human or environmental reservoirs within Haiti). As defined in these experiments, elimination represents a state of no underlying transmission, and not a state of no reported cases. We also recorded the elimination date for each simulation, which was defined as the start of the period in which a simulation achieved less than one cholera infection for at least 52 consecutive weeks.

In addition to elimination metrics, we estimated the cumulative number of infections in a given scenario and calculated the median percentage of infections averted in each scenario after the start of the vaccination campaign (compared with the status quo scenario).

### Role of the funding source

The funders of the study had no role in study design, data collection, data analysis, data interpretation, or writing of the report. The corresponding author had full access to all the data in the study and had final responsibility for the decision to submit for publication.

## Results

Teams performed model selection, model calibration, and assessment of model fit independently (appendix 3 p 12–61). Model 1 and Model 4 were best at capturing the timing of both 2010 and 2011 epidemic peaks, whereas Model 2 best captured the magnitude of the 2010 epidemic peak and missed the 2011 peak entirely (appendix 3 p 7). Model 3 did not calibrate Ouest department until June, 2017, but the model fits for the other nine departments were well calibrated to the timing and magnitude of seasonal oscillations during the epidemic period (appendix 3 p 41). The range of 95% CIs for all model fits captured the actual weekly reported cases in Haiti during the last year of the calibration period (Jan 19, 2018, to Jan 18, 2019), but median estimates overestimated reported cases (appendix 3 p 7). Model 2 and Model 3 median estimates best captured the magnitude of reported cases in the last calibration year. Across models, median estimates for weekly reported cases ranged from 20 (95% CI 0–877) in Model 2 to 832 (0–1004) in Model 4 during the last week of the calibration period (week beginning Jan 12, 2019), when 37 cases were actually reported. We estimated that, as of Jan 19, 2019, 40–93% (range of the median estimates across models) of the Haitian population was susceptible to infection with *V cholerae* (appendix 3 p 11).

**Figure 3:**
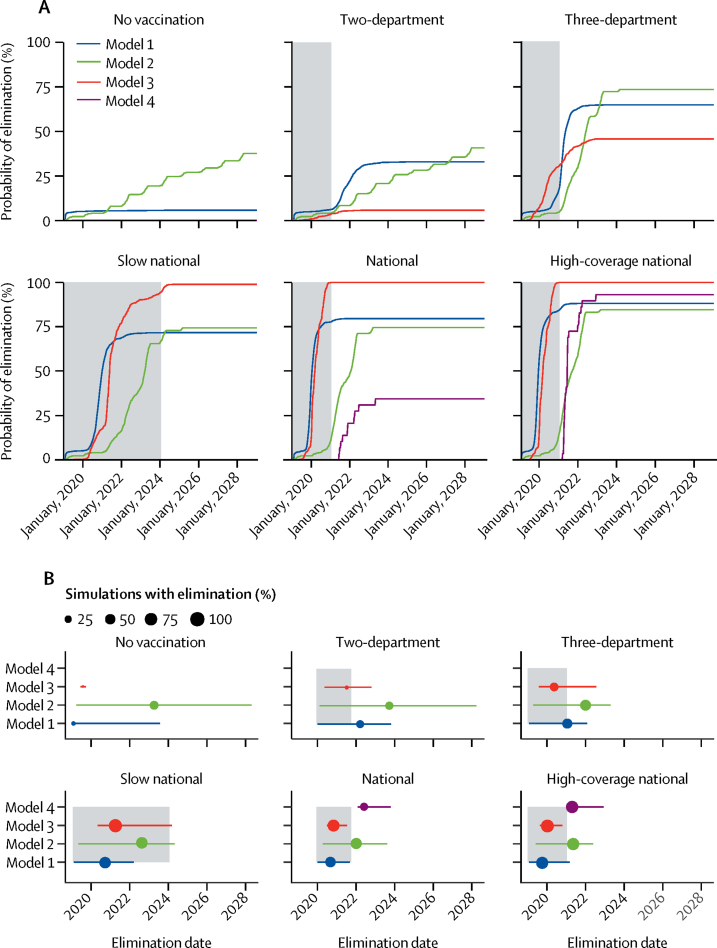
Model outcomes for probability of elimination and elimination date (A) Probability of elimination across simulations during the 10-year projection period across four models in six primary scenarios. (B) Median elimination date (points) and 95% CIs (error bars) for each model and scenario across simulations that achieved elimination. Point size represents the percentage of simulations that achieved elimination. Model scenarios with no depicted information had zero probability of elimination. For each panel, the grey shaded area depicts the duration of the vaccination campaign.

The consensus across the four models is that a 2-year nationwide campaign with coverage similar to that achieved by previous, smaller scale, OCV campaigns in Haiti (70% two-dose coverage)^32^, ^33^ has a moderate chance of achieving true cholera elimination 5 years after the start of the campaign (34–100% of simulations, summarised across teams; figure 3, appendix 3 p 10). If high coverage is achieved (95% two-dose coverage), the models agree that cholera elimination is almost guaranteed after a nationwide campaign (88–100% of simulations). By contrast, simulations with vaccine deployment based on Haiti's most recently published national strategy to eliminate cholera, which aimed to target 1·8 million people primarily in the two most cholera-affected departments (Centre and Artibonite),^34^ suggest that this strategy has a very low probability of achieving elimination (0–33% of simulations) through OCV alone. There was a substantial difference between the outcomes of the two-department and three-department campaigns (the median probability of elimination across models ranged from 0% to 65% 5 years after the start of vaccination campaigns) because the three-department campaign administered more than 2·5 times more doses than the two-department campaign. Although the models were designed primarily to examine the impact of vaccination campaigns, we also projected cholera incidence without future vaccination campaigns, and found that, across models, there was a 0–18% probability of elimination by January, 2024.

We examined the date of elimination (the first day after the start of vaccination campaigns on which there was less than one infection in total for at least 52 consecutive weeks) in model and scenario simulations that achieved elimination (figure 3). The median elimination date for Model 1 was within 1 year of the end of vaccination rollout for the two-department campaign (September, 2021) and three-department campaign (March, 2021), and in the middle of the vaccination rollout for all three national campaigns (ranging from December, 2019, to November, 2020). The median elimination date for Model 2 was mostly outside the vaccination rollout period of the campaigns (ranging from June, 2021, to April, 2024), whereas the median elimination date for Model 3 was always before the end of the vaccination campaigns (ranging from March, 2020, to May, 2021). Model 4 achieved elimination only in the national (median elimination date in November, 2021) and high-coverage national scenarios (median elimination date in June, 2021).

**Figure 4:**
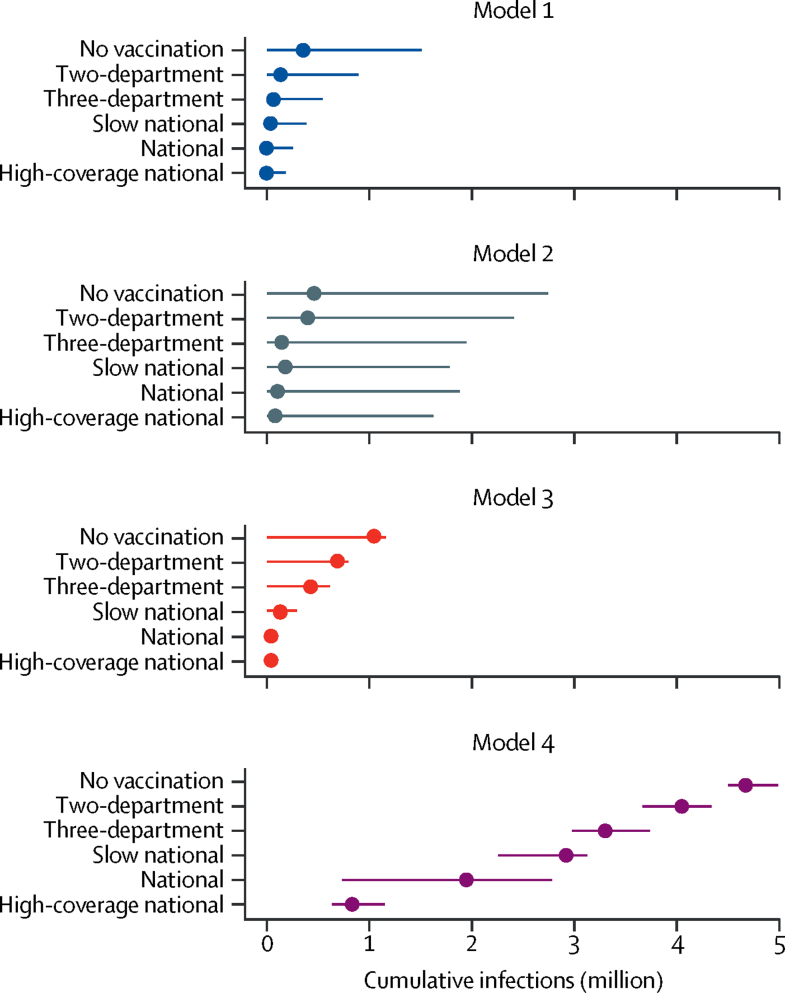
Cumulative infections from February, 2019, to February, 2024 Median estimate (points) with 95% CIs (error bars) of cumulative infections from February, 2019, to February, 2024, by model and projection scenario. The period from February, 2019, to February, 2024, represents a 5-year period from the start of the vaccination campaigns.

We also compared the percentage of averted infections within 5 years of the start of vaccination campaigns for various scenarios (figure 4, appendix 3 p 11). The two-department campaign averted a median of 13–58% of infections, the three-department campaign averted a median of 29–80% of infections, the national 2-year campaign averted 58–94% of infections, the national 5-year campaign averted 37–86% of infections, and the high-coverage national 2-year campaign averted 80–94% of infections across models.

## Discussion

Although the cholera epidemic declined substantially in Haiti in the year after completing this analysis, our results suggest that cholera transmission may persist, with historically observed rates of under-reporting and asymptomatic infection. In 2019, Haiti's national plan for the elimination of cholera proposed to primarily target the two departments with the highest cholera incidence (Centre and Artibonite) for mass vaccination campaigns;^34^ however, our multimodelling study suggests that this strategy would avert 13–58% of infections within a 5-year period and yield a low probability (0–33%) of achieving elimination. Only when the models simulated a nationwide vaccination campaign with a fairly short rollout duration (2 years) did all models project at least some probability of elimination, with 58–95% of infections averted within a 5-year period.

The consideration of mass OCV use in Haiti should not be construed as a call to decrease efforts to improve access to safely managed and sustainable water and sanitation facilities in the country. Instead, the modelresults, which suggest a low probability of elimination under the current conditions, should be used to motivate much larger investments in comprehensive interventions that also include this crucial infrastructure. The UN Haiti Cholera Response Multi-Partner Trust Fund, established in response to the UN's responsibility for introducing *V cholerae* into Haiti, is far from reaching its US$400 million fundraising goal. Haiti's cholera control plan, and indeed the capacity of its health system torespond to epidemic disease, is likewise underfunded. Although the estimated Haitian population living in households with an improved water source increased from 65% to 74% between 2012 and 2017, 32% of the population remained more than 30 min away from a water source by their primary means of travel in 2017 and the largest-scale water and sanitation improvements remain focused on urban areas.^35^ Universal access to clean water and sanitation is a crucial component of the UN Sustainable Development Goals and a human right, and, until further progress is achieved, Haiti remains vulnerable to reintroduction of cholera (appendix 3 p 11). However, halting endemic transmission through vaccination could remove pandemic cholera from the Americas for decades, protecting vulnerable populations from one of the deadliest water-borne pathogens.

The incidence of cholera in Haiti has declined steadily since 2012, and some think that the country could be on track to elimination with current control activities. Indeed, from the completion of our modelling exercise in February, 2019, to the time of writing (June, 2020), no confirmed cases of cholera have been reported in Haiti, although active surveillance has been greatly impacted by sociopolitical unrest.^5^ Comprehensive multisectoral interventions, including identification of cases in the community; appropriate treatment of patients; water, sanitation, and hygiene interventions; and case-area-targeted rapid response teams might have contributed to a change in the evolution of the outbreak, although few studies have measured the impact of individual interventions.^36^, ^37^ Under robust surveillance, observing zero cases for 1 year may support evidence for true elimination (appendix 3 p 10). However, prolonged periods of civil unrest and fuel shortages resulted in extended durations of limited movement in Haiti in 2019,^38^ and, combined with a report of lower-than-expected sensitivity for stool culture,^39^ current surveillance activities could miss some cases.

Further, our modelling shows that periods of low-to-no detected cholera cases followed by disease resurgence are possible (appendix 3 pp 8–9), and resurging outbreaks from the same genetic clade of *V cholerae* in Yemen suggest that resurgence without new introduction is possible in real-world settings.^40^ Model simulations suggest that the probability of true elimination (in contrast to the absence of clinical cases) is low (0–18% of simulations) without changes to current conditions. As immunity from the original outbreak and the 870 000 vaccinated individuals from November, 2016, to May, 2018 (appendix 3 p 11), wanes in Haiti, and as susceptible birth cohorts expand, it is not unreasonable to believe that the country could be at risk of a cholera resurgence. Hence, continued, vigilant surveillance is necessary.

Each model had its own set of limitations with regard to its mechanisms for characterising cholera transmission and vaccine dynamics; not all models in our exercise included spatial heterogeneity in cholera transmission, population movement data, population dynamics, and environmental reservoir or transmission components (appendix 3 pp 12–61). The collective exercise was limited by an absence of data on loss of immunity after natural infection, the importance of environmental reservoirs in cholera transmission, and reporting and asymptomatic rates of cholera in Haiti (appendix 3 p 6), and the inability to predict changes in future disease control measures and dynamic disease transmission regimes. However, all models explicitly incorporated parameter uncertainty to some extent in their estimates, which mitigates some of these limitations. Indeed, the strength of the multimodelling exercise was that model results were interpreted collectively, thus individual model assumptions and parameters were treated as sensitivity analyses for the modelling exercise as a whole.

We interpreted divergence in the model results as a signal of greater uncertainty. Model 1 and Model 2 estimated higher probabilities of elimination for the limited vaccination scenarios than did Model 3 and Model 4 (figure 3A); Model 1 lacked the spatial compartmentalisation that may have reduced the probability of elimination in other models, whereas Model 2 had a longer infectious period, shorter duration of immunity from natural infection, and a constant (but lower, comparable mean) value for vaccine protection during the period of vaccine-induced immunity (appendix 3 pp 6, 12–61). Simulations tended to achieve elimination earlier in Model 1 and Model 3 than in Model 2 and Model 4 (figure 3B), which may be related to the short-to-non-existent persistence of *V cholerae* in the environment in Model 1 and Model 3 (appendix 3 p 6). In general, Model 4 had outlying results (figure 4), but it was the only model that was not separately calibrated to the initial epidemic and later endemic transmission periods. Moreover, as a result of its fine spatial scale, Model 4 was able to sustain cholera transmission up to 2019 only with relatively high transmission rates, leading to a relatively large number of infections of *V cholerae* throughout the projection period.

If vaccine supplies and other resources were unconstrained, a mass vaccination campaign in Haiti would have few disadvantages, since the vaccine has few, if any, side-effects. In reality, OCV supplies are severely limited, resources for public health are insufficient, and mass OCV campaigns no longer seem likely in Haiti given the absence of reported, confirmed cholera cases. Should cases re-emerge, the unique situation in Haiti represents a rare opportunity to use OCV to eliminate cholera from an entire region of the world, rather than as a temporary measure to respond to continuing flare-ups and disasters. Our modelling study suggests that this goal is achievable with a high-quality, large-scale campaign with high population coverage. Such an effort would represent an innovative, and perhaps radical, use of public health resources, but might offer substantial long-term benefits and provide a reprieve to the already stretched public health facilities and emergency response sector in Haiti. If a large-scale OCV campaign prevented future outbreaks for decades, it would ultimately consume fewer resources than maintaining the current conditions.

Our study lends credence to the idea that large vaccination campaigns that result in synchronised immunity in the population can lead to substantial reduction in cholera transmission and may even lead to sustained elimination in settings that are isolated from new *V cholerae* introductions. Recent efforts from the WHO-led Global Task Force on Cholera Control have spurred many cholera-affected countries to begin developing national cholera control plans that integrate activities from several sectors. As we work towards the goal of global cholera elimination, and as cholera-affected countries become surrounded by cholera-free areas with minimal probability of new introductions, our study suggests that national mass vaccination campaigns may become a viable strategy for cholera elimination in other settings. Coupled with investments in large-scale public water and sanitation systems, mass vaccination campaigns could transform the health of millions of people worldwide.

## Supplementary Materials

French translation of the abstract

Kreyol translation of the abstract
