# Supplementary material for: Achieving coordinated national immunity and cholera elimination in Haiti through vaccination: a modelling study
Source: Lancet Glob Health. 2020 Jul 22;8(8):e1081–9. doi: 10.1016/S2214-109X(20)30310-7 (PMC7738665; doi:10.1016/S2214-109X(20)30310-7)
Supplement: French translation of the abstract [file mmc1.pdf]

THE LANCET
Global Health

Supplementary appendix 1

This translation in French was submitted by the authors and we reproduce it as supplied. It has not been peer reviewed. *The Lancet*'s editorial processes have only been applied to the original in English, which should serve as reference for this manuscript.

Cette traduction en français a été proposée par les auteurs et nous l'avons reproduite telle quelle. Elle n'a pas été examinée par des pairs. Les processus éditoriaux de *Lancet* n'ont été appliqués qu'à l'original en anglais, ce qui devrait servir de référence à ce manuscrit.

Supplement to: Lee EC, Chao DL, Lemaitre JC, et al. Achieving coordinated national immunity and cholera elimination in Haiti through vaccination: a modelling study. *Lancet Glob Health* 2020; **8:** e1081–89.

Contexte : En 2010, le choléra a été introduit en Haïti et depuis, plus de 820 000 cas et près de 10 000 morts ont été reportés. Le vaccin anticholérique oral (VCO) est sûr et efficace, mais n'a pas été considéré comme moyen principal pour l'élimination du choléra à cause de la durée limitée de sa protection et de sa disponibilité restreinte. Dans toute la région, le choléra épidémique est présent uniquement sur l'île d'Hispaniola, et l'année 2019 a vu le nombre de cas le plus faible depuis le début de l'épidémie.

Méthodes : Dans cette étude de modélisation, nous avons examiné la probabilité d'élimination, le temps d'élimination, et le pourcentage de cas évités à travers différents scénarios de campagnes de VCO en Haïti avec les simulations de quatre équipes de recherche. Sur une période de 10 ans, du 19 janvier 2019 au 13 janvier 2029, nous avons comparé un scénario sans vaccination (donc en continuant les conditions actuelles) avec cinq scénarios de campagnes de VCO qui diffèrent par l'étendue géographique, la couverture vaccinale, et la durée du déploiement. Les modèles ont été calibrés sur les cas suspects de choléra des rapports hebdomadaires du Ministère de la Santé Publique et de la Population d'Haïti. Les quatre modèles se sont basés sur les mêmes paramètres de vaccination, mais toutes les autres caractéristiques ont été déterminées indépendamment par chaque équipe.

Résultats : Parmi les campagnes de même couverture vaccinale (70% de la population cible pleinement vaccinée), l'écart entre les probabilités médiane d'élimination au bout de 5 ans était de 0-18% pour un scénario sans vaccination ; 0-33% pour une campagne de deux ans ciblant les 2 départements avec l'incidence la plus élevée ; 0-72% pour une campagne sur 3 départements, et 35-100% pour une campagne nationale. Selon les modélisations et sur une période de 5 ans, les campagnes sur 2 départements ont évité une médiane de 12-58% des infections, les campagnes sur 3 départements ont évité une médiane de 29-80% des infections, et les campagnes nationales ont évité 58-95% des infections. En étendant la durée de la campagne nationale pendant cinq ans, par rapport aux campagnes sur deux ans, la probabilité d'élimination et le pourcentage des cas évités ont baissé à 0-95% et à 37-86% respectivement.

Interprétation : Les modélisations suggèrent qu'il est peu probable d'interrompre la transmission de *Vibrio cholerae* en Haïti en poursuivant les stratégies actuelles et que des actions plus audacieuses sont nécessaires pour atteindre l'élimination du choléra dans la région. Les campagnes de vaccination de masse en Haïti présenteraient l'opportunité de synchroniser l'immunité nationale, et donc de protéger la population à court terme, pendant que l'amélioration de la qualité de l'eau et de l'assainissement permettrait l'élimination du choléra à long terme.
