# Supplementary material for: Achieving coordinated national immunity and cholera elimination in Haiti through vaccination: a modelling study
Source: Lancet Glob Health. 2020 Jul 22;8(8):e1081–9. doi: 10.1016/S2214-109X(20)30310-7 (PMC7738665; doi:10.1016/S2214-109X(20)30310-7)
Supplement: Kreyol translation of the abstract [file mmc2.pdf]

THE LANCET
Global Health

Supplementary appendix 2

This translation in Kreyol was submitted by the authors and we reproduce it as supplied. It has not been peer reviewed. *The Lancet*'s editorial processes have only been applied to the original in English, which should serve as reference for this manuscript.

Se otè yo ki soumèt tradiksyon an kreyòl sa epi nou repwodi li jan yo te ba nou li a. Lòt konfrè syan-tifik pa te evalye tradiksyon sa nan kad pwosesis redaksyonèl Lancet yo. Se vèsyon orijinal an anglè a sèlman ki te egzamine swivan pwosesis redaksyonèl Lancet yo epi ki dwe sèvi kòm referans pou maniskri a.

Supplement to: Lee EC, Chao DL, Lemaitre JC, et al. Achieving coordinated national immunity and cholera elimination in Haiti through vaccination: a modelling study. *Lancet Glob Health* 2020; **8:** e1081–89.

Kontèks: Kolera te entwodui an Ayiti an 2010. Depi lè sa a, yo te rapòte plis pase 820000 ka ak prèske 10000 lanmò. Vaksen nan bouch kont kolera a (Oral Cholera Vaccine, OCV) jeneralman san danje epi li efikas, men gen pwofesyonèl sante piblik ki pa te wè li kòm yon zouti prensipal pou eliminasyon kolera akòz dire pwoteksyon li bay ak apwovizyònman li limite. Nan rejyon Amerik Latin ak Karayib epidemi kolera a rete nan limit zile Ispayola, epi pou ane 2019 la yo te rapòte kantite ka ki pi ba depi kòmansman epidemi an. Pakonsekan, Ayiti ka reprezante yon okazyon eksepsyonèl pou elimine kolera avèk OCV.

Metòd: Nan etid modelizasyon sa a, nou itilize similasyon kat ekip modelizasyon pou evalye pwobabilite pou eliminasyon, tan pou eliminasyon, epi pousantaj ka ki evite atravè kèk senaryo kanpay OCV an Ayiti. Pou yon peryòd 10 ane soti 19 janvye 2019 pou rive 13 janvye 2029, nou te konpare yon senaryo san vaksinasyon (sètadi, kontinye ak kondisyon aktyèl yo) avèk senk senaryo kanpay OCV ki te diferan sou plan jewografik, kouvèti vaksinal, epi dire deplwaman. Ekip yo te itilize rapò Ministè Sante Piblik ak Popilasyon Ayisyen an sòti chak semèn sou ka kolera sispèk onivo depatmantal epi menm sipozisyon sou vaksination pou kalibre modèl yo, men yo te detèmine lòt karakteristik modèl yo nan yon fason endepandan.

Rezilta: Pou senaryo kote kanpay yo te genyen menm kouvèti vaksinal (70% moun te vaksine), entèval pwobablite medyàn pou eliminasyon apre 5 ane te: san vaksinasyon: 0-18%; kanpay sou 2 ane nan de (2) depatman kote te genyen plis ka kolera istorikman: 0-33%; kanpay nan twa (3) depatman: 0-72%; kanpay nasyonal: 35-100%. Kanpay nan de (2) depatman te evite an medyàn 12-58% enfeksyon; kanpay nan twa (3) depatman te evite an medyàn 29-80% enfeksyon; kanpay nasyonal te evite an medyàn 58-95% enfeksyon. Ekstansyon kanpay nasyonal la sou 5 ane a, konpare a 2 ane, redwi pwobalite eliminsayon a 0-95%, epi redwi kantite ka evite a 37-86%.

Entèpretasyon: Modèl yo sijere ke pwobabilite a fèb pou Ayiti rive a zewo transmisyon ka *Vibrio cholerae* avèk metòd kontwòl aktyèl yo, epi aksyon ki pi desisif nesesè pou favorize eliminasyon kolera nan rejyon an. Kanpay vaksinasyon kont kolera nan yon kad elaji an Ayiti tap ofri posiblite pou ranfòse pwoteksyon iminitè anmenmtan nan tout peyi a, sa ki tap bay yon pwoteksyon akoutèm nan popilasyon an, pandan amelyorasyon sistèm dlo ak asenisman ap favorize eliminasyon kolera alontèm.
