# Supplementary material for: Achieving coordinated national immunity and cholera elimination in Haiti through vaccination: a modelling study
Source: Lancet Glob Health. 2020 Jul 22;8(8):e1081–9. doi: 10.1016/S2214-109X(20)30310-7 (PMC7738665; doi:10.1016/S2214-109X(20)30310-7)
Supplement: Supplementary appendix 3 [file mmc3.pdf]

THE LANCET
Global Health

Supplementary appendix 3

This appendix formed part of the original submission and has been peer reviewed. We post it as supplied by the authors.

Supplement to: Lee EC, Chao DL, Lemaitre JC, et al. Achieving coordinated national immunity and cholera elimination in Haiti through vaccination: a modelling study. *Lancet Glob Health* 2020; **8:** e1081–89.

Supplementary Material: Achieving coordinated national immunity and cholera elimination in Haiti through vaccination - a modeling study

Elizabeth C. Lee[1*], Dennis L. Chao[2*], Joseph Lemaitre[3*], Laura Matrajt[4*], Damiano Pasetto[3,5], Javier Perez-Saez[3], Flavio Finger[6,7], Andrea Rinaldo[3], Jonathan D. Sugimoto[4], M. Elizabeth Halloran[4,8], Ira M. Longini Jr.[9,10], Ralph Ternier[11], Kenia Vissieres[11], Andrew S. Azman[1†], Justin Lessler[1†], and Louise C. Ivers[12,13†‡]

[1]Department of Epidemiology, Johns Hopkins Bloomberg School of Public Health, Baltimore, MD, USA
[2]Institute for Disease Modeling, Bellevue, WA, USA
[3]Laboratory of Ecohydrology, School of Architecture, Civil and Environmental Engineering, École Polytechnique Fédérale de Lausanne, Lausanne, Switzerland
[4]Vaccine and Infectious Disease Division, Fred Hutchinson Cancer Research Center, Seattle, WA, USA
[5]Department of Environmental Sciences, Informatics and Statistics, Ca' Foscari University of Venice, Venice, Italy
[6]Centre for Mathematical Modelling of Infectious Diseases, London School of Hygiene and Tropical Medicine, London, UK
[7]Department for Infectious Disease Epidemiology, Faculty of Epidemiology and Population Health, London School of Hygiene and Tropical Medicine, London, UK
[8]Department of Biostatistics, University of Washington, Seattle, WA, USA
[9]Department of Biostatistics, College of Public Health and Health Professions, University of Florida, Gainesville, FL, USA
[10]Emerging Pathogens Institute, University of Florida, Gainesville, FL, USA
[11]Partners In Health/Zanmi Lasante, Port-au-Prince, Haiti
[12]Department of Global Health and Social Medicine, Harvard Medical School, Boston, MA, USA
[13]Center for Global Health, Massachusetts General Hospital, Boston, MA, USA
[*]denotes equal contribution
[†]denotes co-senior author
[‡]denotes corresponding author

Contents

1 Introduction to the modeling exercise **3**
1.1 Methods 3
1.1.1 Data Sources 3
1.1.2 Vaccine Campaign Logistics 4
1.1.3 Vaccine efficacy 5
1.1.4 Summary of model parameters 6
1.1.5 Data and model access 6
1.2 Results 7
1.2.1 Model calibration 7
1.2.2 Model projections 7
1.2.3 Probability of Elimination 10
1.2.4 Infections Averted 10
1.2.5 Re-accumulation of Susceptible Individuals 10

2 Model-1 Supplement **12**
2.1 Model Description 12
2.1.1 Base Model Structure 12
2.1.2 Modeling Vaccination Campaigns 13
2.2 Model Calibration 15
2.2.1 Epidemic phase 15
2.2.2 Endemic phase 16
2.3 Assessment of Model Fit 17
2.4 Simulation of Vaccination Campaigns 20
2.4.1 Deployment of Vaccination Campaigns 20
2.5 Calculating Elimination Measures 21

3 Model-2 Supplement **23**
3.1 Model Description 23
3.2 Model Fitting/Calibration 31
3.2.1 Assessment of Model Fit 33
3.2.2 Computation of summary statistics 33

4 Model-3 Supplement **34**
4.1 Model Description 34
4.1.1 Model Dynamics 34
4.1.2 Model equations 37
4.1.3 Additional Data 38
4.1.4 Model runs 39
4.2 Model Selection and Fitting/Calibration 40
4.3 Assessment of Model Fit 40

5 Model-4 Supplement **44**
5.1 Model Description 44
5.2 Model Selection and Fitting/Calibration 49
5.3 Assessment of Model Fit 51
5.4 Simulation of Vaccination Campaigns 58
5.5 Calculating Elimination Measures 59

Introduction to the modeling exercise

The primary objective of this modeling exercise was to forecast the potential for oral cholera vaccination (OCV) campaigns to eliminate cholera from Haiti. We formed a modeling consortium of research teams that had previously modeled cholera transmission dynamics in Haiti [1, 2, 3]. For ease of comparison, we decided upon common parameters and assumptions related to vaccine protection and vaccine campaign logistics. We shared common data sources, used a common definition of elimination across models and produced comparable outputs for figures and analyses. All other modeling decisions and assumptions were left to the discretion of each team.

All teams adapted and improved upon their previously published models of Haiti transmission dynamics for this new modeling effort. In brief, teams fit their models to a common cholera incidence data source, using the different slices of the data according to their model structures and assumptions. Then they generated model projections of true and reported cholera incidence 10 years past the end of the data available for model fitting. Multiple 10-year projections were produced by each model; a *status quo* scenario and five primary vaccination campaign scenarios that differ by deployment and vaccination coverage. To coordinate the modeling efforts, we held bi-weekly phone calls to discuss progress, troubleshoot problems, and settle on common assumptions and timelines.

We discussed the goals of the project and methods with partners in the Haiti Ministry of Public Health and Population (MSPP) at the onset of this initiative for feedback on the approach and primary assumptions. Once work had started, we had multiple consultative meetings in Haiti and by teleconference with epidemiologists, researchers and clinicians that had were involved in the cholera response in Haiti in their individual capacities.

1.1 Methods

1.1.1 Data Sources

All teams fit their models to publicly available weekly department-level cholera reports of suspected cases from the MSPP website [4]. Data were available for the week of October 23, 2010 through the week of January 12, 2019.

Teams also had the option to use the following additional shared data sources, formally or informally in to calibrate or validate the models:

1. The number of confirmatory tests (culture) conducted and the number positive in 2016 and 2017 from US CDC and MSPP;

2. The number of doses administered and fully vaccinated persons from coverage surveys of historical deployments of OCV in Haiti among selected communes in Grand'Anse (first round: November 2016; second round: May to June 2017), Sud (first round: November 2016 to January 2017; second round: May to June 2017), Ouest (first round: July 2017; second round: August 2017), Centre (first round: November 2017; second round: December 2017), and Artibonite (first round: April 2018; second round: May 2018) (Table S1).

Table S1: Mass OCV campaigns in Haiti after Hurricane Matthew. FVPs stands for fully vaccinated persons. Source: Global Task Force on Cholera Control

Department	Commune	Round 1 dates	Round 2 dates	Target Population	Doses Round 1	Doses Round 2	FVPs
Grand'Anse	Anse d'Hainault	11/8/2016-11/18/2016	5/26/2017-6/2/2017	37616	32184	22840	21255
Grand'Anse	Beaumont	11/8/2016-11/18/2016	5/26/2017-6/2/2017	32635	30486	19298	17270
Grand'Anse	Bonbon	11/8/2016-11/18/2016	5/26/2017-6/2/2017	8900	9716	6965	5688
Grand'Anse	Chambellan	11/8/2016-11/18/2016	5/26/2017-6/2/2017	27342	26168	20839	18979
Grand'Anse	Dame Marie	11/8/2016-11/18/2016	5/26/2017-6/2/2017	40041	43335	29068	26480
Grand'Anse	Jeremie	11/8/2016-11/18/2016	5/26/2017-6/2/2017	138802	138463	110950	90245
Grand'Anse	Les Irois	11/8/2016-11/18/2016	5/26/2017-6/2/2017	24155	25213	19936	17882
Grand'Anse	Moron	11/8/2016-11/18/2016	5/26/2017-6/2/2017	32197	27973	22600	19732
Grand'Anse	Pestel	11/8/2016-11/18/2016	5/26/2017-6/2/2017	46149	38210	31734	26897
Sud	Aquin	11/8/2016-11/18/2016	5/27/2017-6/2/2017	107695	90738	87105	70633
Sud	Camp Perrin	11/8/2016-11/18/2016	5/27/2017-5/31/2017	46547	44773	43663	39029
Sud	Chardonnieres	11/8/2016-11/18/2016	5/27/2017-5/31/2017	26083	16036	17815	15989
Sud	Coteaux	12/9/2016-12/15/2016	5/27/2017-5/31/2017	22014	13799	17889	16487
Sud	Les Anglais	11/8/2016-11/18/2016	5/27/2017-6/2/2017	30888	35172	21184	19300
Sud	Les Cayes	11/8/2016-11/18/2016	5/27/2017-6/2/2017	156762	162791	134432	110318
Sud	Port-a-Piment	11/8/2016-11/18/2016	5/27/2017-6/1/2017	19554	12339	13490	11748
Sud	Port Salut	11/8/2016-11/18/2016	5/27/2017-6/1/2017	19736	22594	20370	16017
Sud	Tiburon	1/12/2017-1/18/2017	5/27/2017-6/1/2017	20437	9355	13965	11366
Ouest	Port-au-Prince	7/25/2017-7/28/2017	8/22/2017-8/25/2017	4053	3106	3314	2829
Centre	Mirebalais	11/15/2017-11/21/2017	12/10/2017-12/16/2017	98564	88377	85112	69905
Artibonite	Saint Miche de l'Attalaye	4/17/2018-4/23/2018	5/9/2018-5/15/2018	62241	62469	64743	59537

1.1.2 Vaccine Campaign Logistics

Rollout

We implemented four vaccine campaign deployment scenarios, each starting the day or week (depending on model implementation) after the last data point used for model calibration (week of January 12, 2019). Campaigns targeted departments in order of 2017-2018 cumulative incidence from highest to lowest (Table S2).

Table S2: Departments in Haiti, ordered by cumulative incidence from 2017-2018

Campaign Order	Department	Population (2015)	Incidence per 1K (2017/18)
1	Centre	746,236	4.30
2	Artibonite	1,727,524	2.73
3	Ouest	4,029,705	1.60
4	Nord Ouest	728,807	1.14
5	Nord	1,067,177	1.02
6	Sud	774,976	0.65
7	Nippes	342,525	0.62
8	Nord Est	393,967	0.42
9	Sud Est	632,601	0.36
10	Grand'Anse	468,301	0.25

In our rollout scenarios, we aimed to capture the potential impacts of three general types of vaccination approaches: (1) rapid vaccination of a limited geographic area; (2) rapid vaccination of the entire country; and (3) national vaccination over a longer rollout period. Specifically, the campaign scenarios are:

- **Two-department**: Vaccination limited to the departments of Centre and Artibonite over a 2-year period, similar to plans outlined in the national cholera elimination plan for Haiti [5].
- **Three-department**: Vaccination limited to the departments of Centre, Artibonite, and Ouest (which includes the populous Port-au-Prince) over a 2-year period.
- **Fast national** (named 'National' in the main text): Countrywide vaccination implemented over a 2-year period.
- **Slow national**: Countrywide vaccination implemented over a 5-year period.

Vaccination Coverage

Killed OCVs are licensed as a two-dose regimen, with doses taken at least two weeks apart [6]. All simulated campaigns aimed to vaccinated everyone with two doses, however, following data from previous vaccination campaigns, a fraction of individuals only received a single dose and some remained unvaccinated. In our primary simulations, we assume that vaccine coverage is the same in all departments with 70% two-dose coverage, 10% one-dose coverage and 20% receiving no vaccine. We also simulated one 'high coverage' campaign, where departments achieved 95% two-dose coverage, 1.67% one-dose coverage and 3.33% unvaccinated at the end of the campaign.

Combining vaccination rollout and coverage scenarios, we had simulated results for five vaccination scenarios that required different numbers of vaccines (Table S3).

Table S3: Roughly, the number of vaccines needed to complete each vaccination deployment scenario.

Scenario	Number of OCV Doses Needed
2-department	3.71 million
3-department	9.76 million
slow national	16.37 million
fast national	16.37 million
high coverage fast national	20.91 million

1.1.3 Vaccine efficacy

Waning vaccine efficacy among adults was pre-set (Figure 2, Table S4). All teams assumed that initial vaccine efficacy was 76%, as estimated by a recent case-control study in Haiti (Figure 2) [7]. We estimated waning vaccine efficacy for 60 months after vaccination by fitting a log-linear weighted regression model to the raw data from a published meta-analysis on killed OCV efficacy (excluding a single outlier estimate of vaccine efficacy in India after 5 years to be conservative) (Figure 2) [6]. We assumed that the vaccine afforded no protection after the end of five years.

The average vaccine efficacy among children under five years old was 46.9% (0.30/0.64) as effective as that in adults [6]. As there are limited data on vaccine efficacy among children, we used this ratio as a conservative multiplier to adjust the adult vaccine efficacy for vaccine efficacy among children under five (Table S4).

In the first year after vaccination, individuals who received a single dose of vaccine were assumed to have the same protection as those with two doses, after which period the single dose efficacy drops to zero (Table S4).

Table S4: Vaccine efficacy assumptions for adults and children used in modeling scenarios.

Months after vaccination	2-Dose Efficacy, Adults (%)	1-Dose Efficacy, Adults (%)	2-Dose Efficacy, Children (%)	1-Dose Efficacy, Children (%)
0	76	76	36	36
6	72	72	34	34
12	68	68	32	32
18	63	0	30	0
24	58	0	27	0
30	52	0	24	0
36	46	0	22	0
42	39	0	18	0
48	32	0	15	0
54	24	0	11	0
60	15	0	7	0

1.1.4 Summary of model parameters

While not all models utilize the same parameters, we compiled a table with comparable model parameters (Table S5).

Table S5: Summary of shared parameters across models.

Parameters	Model-1	Model-2	Model-3	Model-4
Mean percentage of infections reported as cases	48	20	97 (before 1 Jan 2018) 10 (after 1 Jan 2018)	30
Relative risk of transmission from asymptomatic to symptomatic individuals	0.05	0.001	0.043	0.1
Mean persistence of V. cholerae in the environmental reservoir (days)	NA	21	2.8	14
Mean duration of immunity after natural infection (years)	8	5	8	8
Duration of infectious period (days)	2	7	5	1-3

1.1.5 Data and model access

All modeling teams have provided a detailed supplementary methods and results section, which are assembled collectively at the summary DOI: 10.5281/zenodo.3361800. We performed an internal review of these supplementary documents in order to enhance the readability of these materials. Teams have also made available input data and model code at the following DOIs:

- Model-1, Johns Hopkins Bloomberg School of Public Health: 10.5281/zenodo.3360991
- Model-2, Fred Hutchinson Cancer Research Center and University of Florida: 10.5281/zenodo.3360857
- Model-3, École Polytechnique Fédérale de Lausanne: 10.5281/zenodo.3360723
- Model-4, Institute for Disease Modeling: 10.5281/zenodo.3360885

1.2 Results

1.2.1 Model calibration

We present all four model fits to weekly reported cholera cases from MSPP across Haiti from October 23, 2010 through the week of January 12, 2019 (Figure S1). Model-3 does not calibrate its model for Ouest department in Haiti until the week of June 10, 2017, which is why there is no national fit reported until time (See Model-3 Supplement for details).

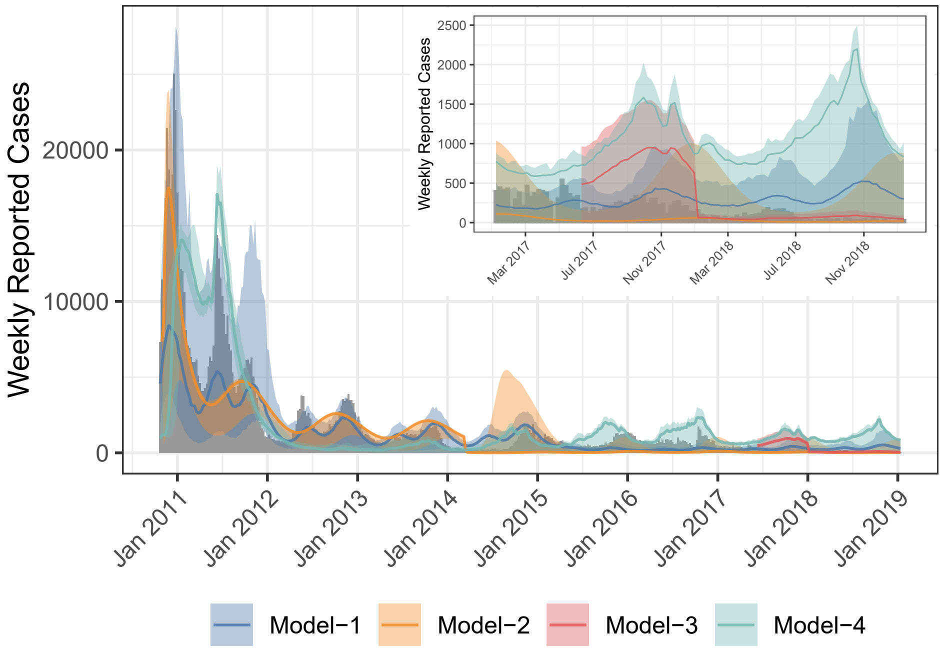

Figure S1: Model calibration to weekly reported cholera cases from MSPP (grey bars) across Haiti from October 23, 2010 through the week of January 12, 2019. The solid lines represent the median and the shaded areas represent 95% CIs. The inset presents the same model calibration zoomed into the period from January 2017 through January 2019. Model-3 has no national-level calibration before June 10, 2017, so that model is not plotted before that time.

1.2.2 Model projections

We present the projections of reported cholera cases and true infection with *Vibrio cholerae* for the status quo and five vaccination scenarios (Figures S2 and S3). Here, 'observed cholera cases' include all symptomatic and reported cases and 'infections' include all cases that may result in onward transmission of disease (e.g., reported and unreported cases, symptomatic and potentially asymptomatic cholera).

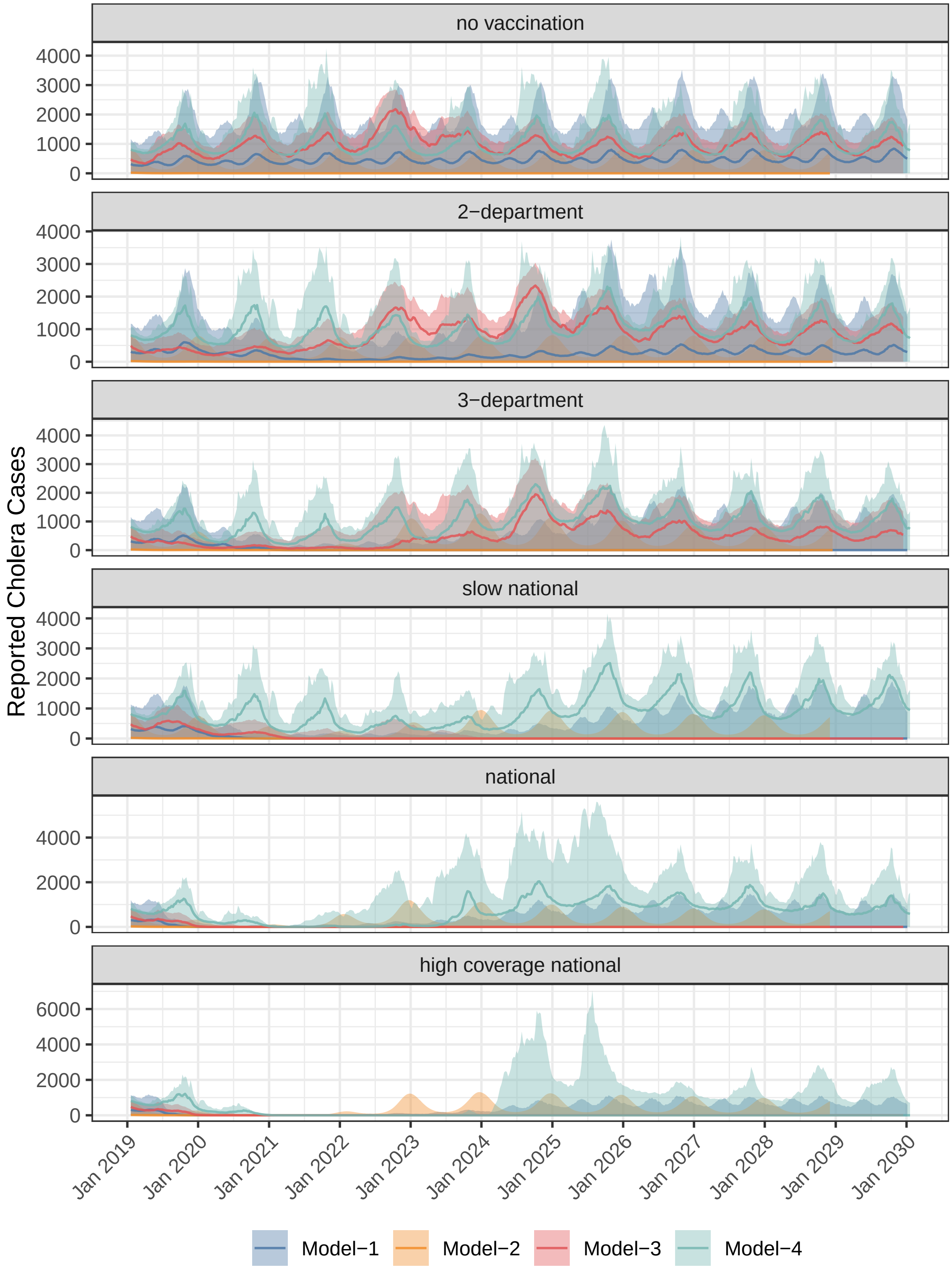

Figure S2: Projections of reported cholera cases for the no vaccination (‘status quo‘ in the main text) scenario and five vaccination scenarios across teams. Lines represent the median estimate for each model and the shaded ribbon represents an upper and lower bound interval, as appropriate to the model.

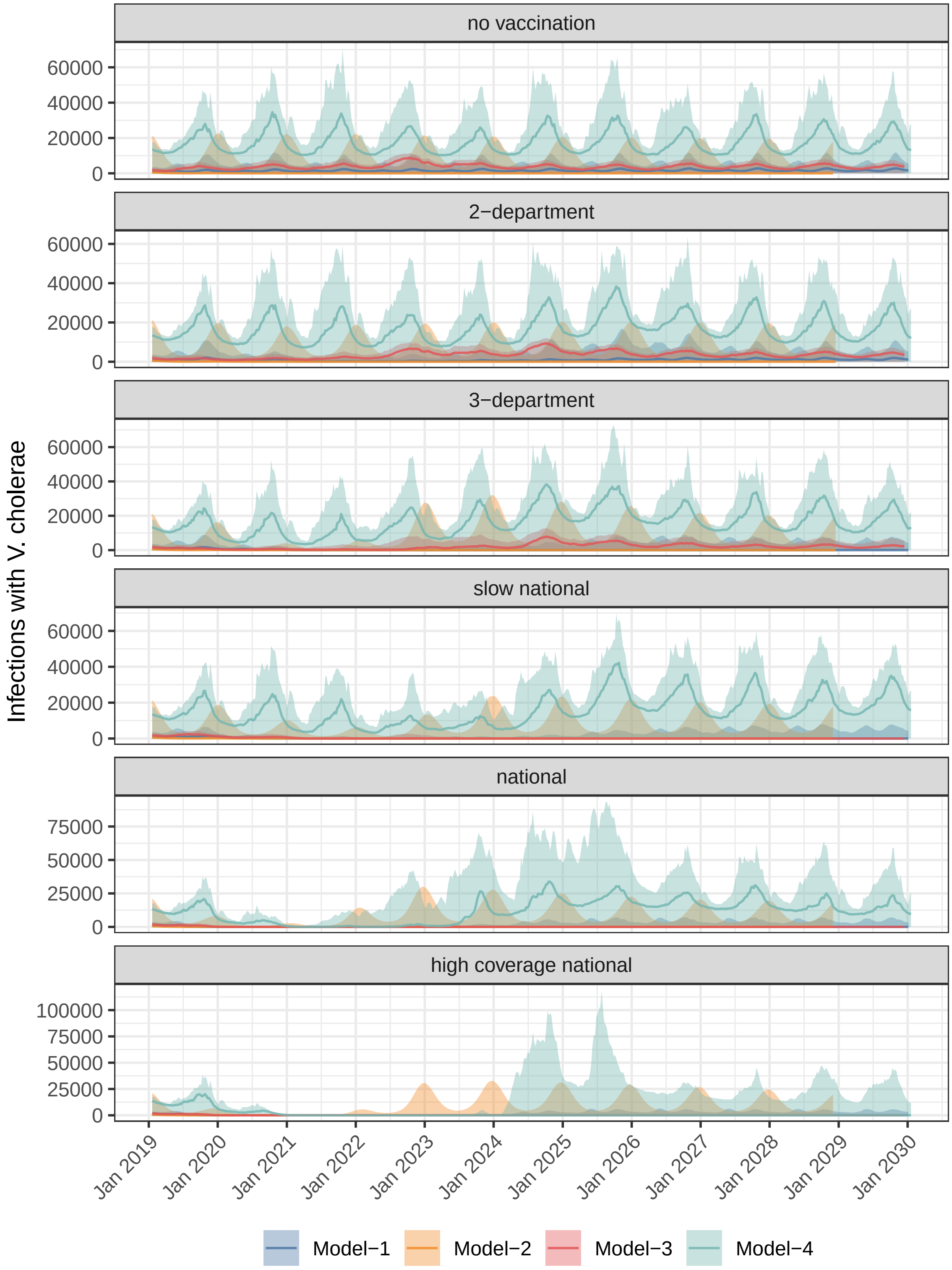

Figure S3: Projections of infections with *Vibrio cholerae* for the no vaccination ('status quo' in the main text) scenario and five vaccination scenarios across teams. Lines represent the median estimate for each model and the shaded ribbon represents an upper and lower bound interval, as appropriate to the model.

1.2.3 Probability of Elimination

Next we present summaries of probability of elimination for the no vaccination (status quo) and five vaccination scenarios at five years after the start of vaccination campaigns (February 2024) (Table S6). The February 2024 time point is the earliest time point after the end of all vaccination campaign scenarios.

Table S6: Probability of elimination (%) 5 years after the start of vaccination campaigns across scenarios and teams.

Model	no vaccination	2-department	3-department	slow national	fast national	high coverage fast national
1	5.8	32.7	64.5	71.6	79.6	88.2
2	35.2	36.3	77.7	72.5	79.5	87.6
3	4.1	9.6	48.2	94.4	100	100
4	0	0	0	0	34.5	93.1

Since the end of our calibration period until February 2020, there have been 0 confirmed cholera cases in Haiti. We examined the probability of observing less than 1 case from January 2019 to January 2020 in our model projections (Table S7).

Table S7: Probability of observing 0 cases and true elimination from January 2019 to January 2020.

Model	Probability of observing 0 cases from January 2019 to January 2020 (%)	Probability of true elimination conditional on probability of observing 0 cases from Jan 2019-2020 (%)
1	5	100
2	22	100
3	2	100
4	0	NA

1.2.4 Infections Averted

We calculated the median number of infections averted (includes observed and unobserved cholera cases) 5 years after the start of vaccination campaigns across scenarios (Figures 4 and S4). Fast national campaigns were the most effective in reducing cases, but a 3-department campaign averted roughly twice as many cases as a 2-department campaign. The slow national campaign and 3-department campaigns have relatively similar health outcomes, thus highlighting the trade-offs between a smaller and faster rollout versus a larger and prolonged campaign.

1.2.5 Re-accumulation of Susceptible Individuals

Haiti MSPP did not report any laboratory-confirmed cholera cases between February 2019 and January 2020. Consequently, we examined the re-accumulation of susceptible individuals in Haiti in our model estimates and projections in the no vaccination scenario from June 2017 through 2030. Three median model estimates projected that 80-93% of the population was susceptible in January 2020; the fourth model projected that 39% of the population was susceptible at that time S5.

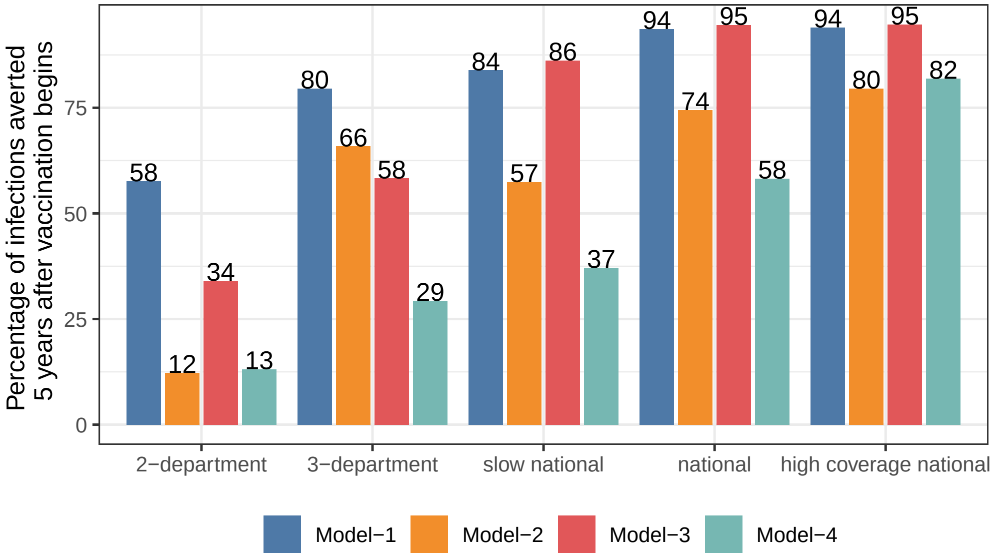

Figure S4: Median infections averted from February 2019 to February 2024. This period represents 5 years after the start of vaccination campaigns across scenarios and teams.

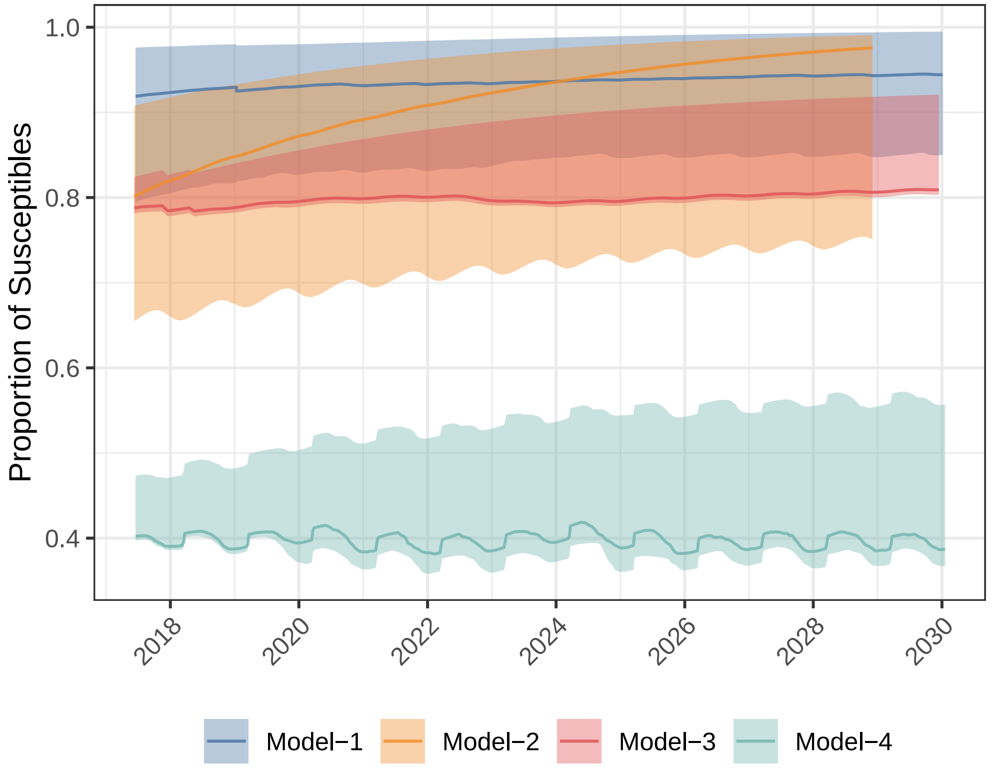

Figure S5: Calibrated and projected proportion of susceptible individuals in the population in the status quo from June 2017 through through 2029.

Model-1 Supplement

Elizabeth C. Lee, Andrew S. Azman, and Justin Lessler
Johns Hopkins Bloomberg School of Public Health

2.1 Model Description

In this study, we adapted a deterministic compartmental model that had been used previously to compare the potential impact of one-dose and two-dose oral cholera vaccine regimens in Port-au-Prince for use at the country-level in Haiti [3]. In brief, the original version of this model made simple assumptions about cholera transmission and disease course; the model assumed Susceptible - Exposed - Infectious - Recovered (SEIR) dynamics; the city population of Port-au-Prince experienced equal potential disease contact with all other individuals in the population (homogeneous mixing); and disease transmission occurred through person-to-person contact with added variability according to a seasonally-varying transmission term. Due to the outbreak-response-focus of the initial study, waning immunity was not considered in this original model. Importantly, there were no explicit model components capturing transmission due to contact with contaminated environments, seasonality in transmission due to climatic changes (e.g., rainfall), or spatial heterogeneities in transmission.

2.1.1 Base Model Structure

For this study, we constructed a continuous-time, discrete-state Susceptible - Exposed - Infectious - Asymptomatic - Recovered - Susceptible (SEIARS) compartmental model with population dynamics. The Infectious state represents symptomatic, infectious individuals, while the Asymptomatic state represents asymptomatic individuals, who are only a fraction as infectious as Infectious individuals. We model loss of immunity among recovered individuals, births into the susceptible population, and deaths by natural causes from all compartments due to the long-term projection of vaccine effects. Compared to the original model, we allow for the possibility of non-homogeneous mixing among potential disease-causing contacts in the population, using methods similar to those used in classic time series Susceptible - Infectious - Recovered (TSIR) models [8, 9], due to observed differences in disease dynamics across departments in Haiti and the country-level scale of our model. The original model included a term for introductions into the modeled city from the rest of the country, but we removed this term since introductions into Haiti are thought to be extremely rare [10].

The underlying dynamics of our model may be represented by the following set of differential equations:

$$\begin{aligned}
\frac{dS}{dt} &= -\lambda S - \delta S + \alpha R + \mu N \\
\frac{dE}{dt} &= -\sigma(1-\theta_0)E - \sigma\theta_0 E - \delta E + \lambda S \\
\frac{dI}{dt} &= -\gamma I - \delta I + \sigma(1-\theta_0)E \\
\frac{dA}{dt} &= -\gamma A - \delta A + \sigma\theta_0 E \\
\frac{dR}{dt} &= -\alpha R - \delta R + \gamma I + \gamma A
\end{aligned}$$

where λ is the force of infection, δ is the natural death rate, α is the rate of recovery from infection, μ is the birth rate, $1/\sigma$ is the latent period, θ_0 is the proportion of Exposed individuals that become Asymptomatic infections,

$1/\gamma$ represents the mean infectious period, and S, E, I, A, R, N represent the Susceptible, Exposed, symptomatic Infectious, Asymptomatic infectious, Recovered, and total populations, respectively. The force of infection is modeled as:

$$\lambda = (I + (1 - \kappa)A)^{\nu}\beta/N,$$

where κ is the relative reduction in infectiousness for Asymptomatic individuals as compare to Infectious individuals, ν is the population mixing coefficient that captures deviations from homogeneous mixing, N is the (current) total population size, and β is the seasonal transmission term. We note that only vaccinated individuals may develop Asymptomatic infections.

Seasonal transmission is modeled as:

$$\beta = \sum_{j=1}^{6} \beta_j s_j$$

from j periodic basis spline terms (s_j) with six basis splines each with six degrees of freedom [3]. We construct the seasonal transmission term with basis splines in order to calibrate the shape, phase, and magnitude of the seasonal component simultaneously; this enables variable complexity in the seasonality of cholera transmission.

We assumed that our cholera incidence surveillance data did not represent all symptomatic cholera infections in Haiti, and thus would be best represented by a partially-observed Markov Process. Thus, the likelihood of our model was

$$L(\boldsymbol{X}|cases) = NegBinom(\rho\xi, \tau)$$

where $\boldsymbol{X}$ is the vector of model parameters and the observation process is parameterized with the mean, which was the product of reporting rate ρ and incidence of new symptomatic infections ξ, and the inverse dispersion τ.

2.1.2 Modeling Vaccination Campaigns

Vaccine Effects

Vaccination has two direct effects in the model:

1. Vaccinated individuals in the Exposed compartment had some probability of becoming Asymptomatic infections; this probability ψ_t was equal to adult vaccine efficacy t months after vaccination. Multiple waning vaccine efficacy scenarios were pre-determined by the modeling exercise.

2. Asymptomatic individuals are assumed to be less infectious than Infectious individuals; the force of infection term λ modifies the relative reduction in infectiousness with the parameter κ, which was fixed to 0.95 in our model (Asymptomatic individuals were 95% less infectious than Infectious individuals).

Tracking Vaccinated Individuals

Vaccination campaigns are tracked as cohorts for each department, and each cohort is represented by another set of Susceptible - Exposed - Infectious - Asymptomatic - Recovered compartments. Thus, the base model with vaccination is modified to:

$$\frac{dS}{dt} = -\lambda S - \delta S + \alpha R + \mu N - \sum_{i=1}^{d_i} \eta_{ik} S$$
$$\frac{dE}{dt} = -\sigma(1-\theta_0)E - \sigma\theta_0 E - \delta E + \lambda S - \sum_{i=1}^{d_i} \eta_{ik} I$$
$$\frac{dI}{dt} = -\gamma I - \delta I + \sigma(1-\theta_0)E - \sum_{i=1}^{d_i} \eta_{ik} E$$
$$\frac{dA}{dt} = -\gamma A - \delta A + \sigma\theta_0 E - \sum_{i=1}^{d_i} \eta_{ik} A$$
$$\frac{dR}{dt} = -\alpha R - \delta R + \gamma I + \gamma A - \sum_{i=1}^{d_i} \eta_{ik} R$$

where i indicates the department, d_i is the number of departments vaccinated in a given scenario, and η_{ik} is the vaccination rate in a department in model week k. While a real departmental vaccination campaign would likely take place over many weeks, we push all of the vaccines for a given department into our model in a single week at the end of the rollout period. This model implementation enables easier tracking of waning vaccine immunity and is conservative in the effects of the vaccine since vaccine effects are experienced only at the very end of the vaccination campaign period. Thus, the vaccination rate η_{ik} is only non-zero during a single week that represents the rollout of a given departmental campaign, according to Table S12. Each of the d_i vaccination cohorts may be represented as:

$$\frac{dSv_i}{dt} = -\lambda Sv_i - \delta Sv_i + \alpha Rv_i + \mu Nv_i + \eta_{ik} S$$
$$\frac{dEv_i}{dt} = -\sigma(1-\theta_{vk})Ev_i - \sigma\theta_{vk} Ev_i - \delta Ev_i + \lambda Sv_i + \eta_{ik} E$$
$$\frac{dIv_i}{dt} = -\gamma Iv_i - \delta Iv_i + \sigma(1-\theta_{vk})Ev_i + \eta_{ik} I$$
$$\frac{dAv_i}{dt} = -\gamma Av_i - \delta Av_i + \sigma\theta_{vk} Ev_i + \eta_{ik} A$$
$$\frac{dRv_i}{dt} = -\alpha Rv_i - \delta Rv_i + \gamma Iv_i + \gamma Av_i + \eta_{ik} R$$

where θ_{vk} represents is the population vaccine efficacy at week k. Population vaccine efficacy was calculated as

$$\theta_{vk} = \psi_t * (1 - (1 - 0.4688) * 0.11),$$

where ψ_t, the adult vaccine efficacy at month t after vaccination, is scaled to the population-weighted average of vaccine efficacy among people over and under five years old. Vaccine efficacy among children under five was assumed to be 0.4688 times as effective as vaccine efficacy among adults [6], and 11% of the Haitian population was identified as under five years old [11]. A single dose of cholera vaccine was assumed to confer the same level of protection as two doses in the first year and no protection in subsequent years of vaccination.

The force of infection λ is the same for Susceptible individuals in all vaccination cohorts

$$\lambda = ((I + \sum_{i=1}^{d_i} Iv_i) + (1-\kappa)(A + \sum_{i=1}^{d_i} Av_i))^{\nu} \beta / N,$$

so the vaccination cohort structure is used solely for tracking the time of vaccination and applying the appropriate vaccine efficacy for a given time point in the model. Figure S6 depicts the model structure for a two department vaccination campaign.

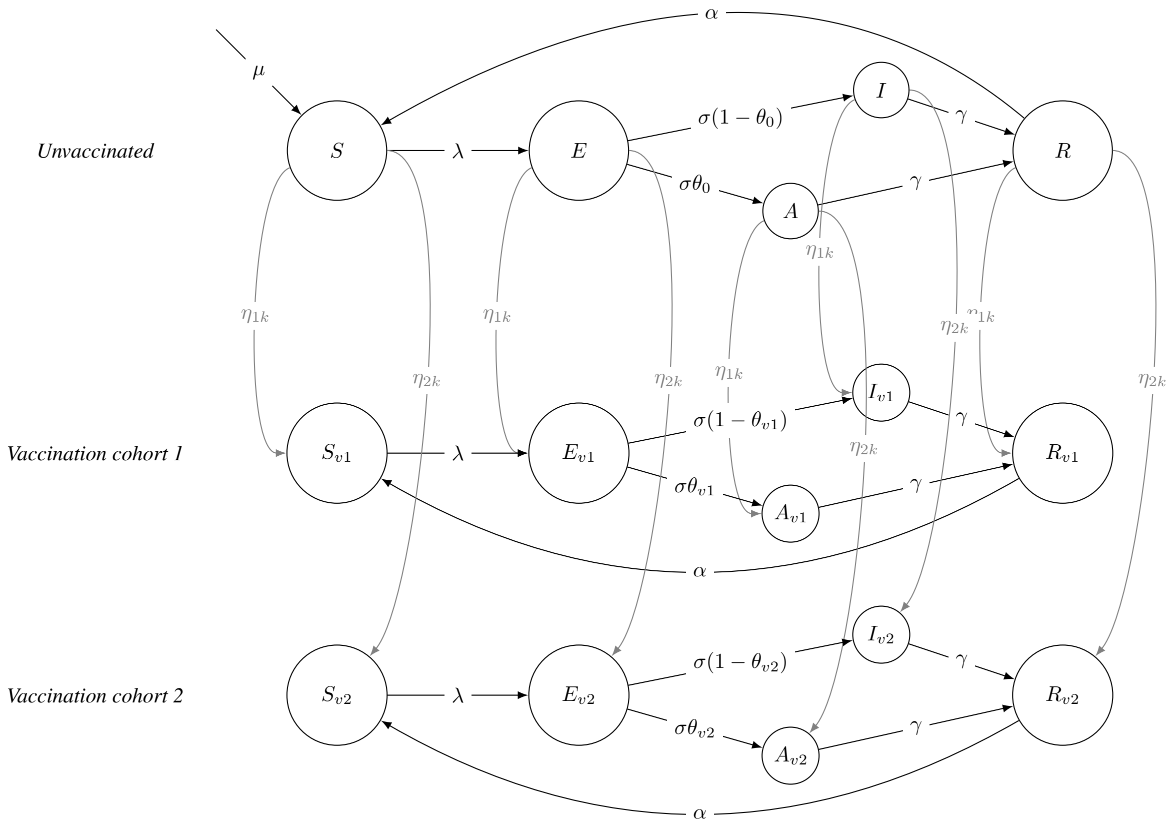

Figure S6: Demonstrative flow diagram of the model structure for a two department vaccination campaign. Death rates (δ) from all compartments are not shown to improve diagram readability.

2.2 Model Calibration

We calibrated our model in two stages using the R package POMP (v1.19) [12, 13]. The first stage calibrated the model during the "epidemic" phase of the data, which we assumed was from October 2010 through March 2015 with the second "epidemic" stage going from April 2015 through January 2019 (end of the calibration period). The model was parameterized with parameters scaled to week-long time steps and model states were extracted at weekly intervals, in line with data reports.

2.2.1 Epidemic phase

We profiled four parameters using Latin Hypercube Sampling, reporting rate (ρ), the first component of the basis spline (β_1), population mixing coefficient (ν), and inverse dispersion (θ), to generate 300 sets of starting values that covered a wide range of parameter space (Table S8). Fixed and fitted parameters are described in Tables S9 and S10.

We first used trajectory matching to perform maximum likelihood estimation on our model's deterministic skeleton. We passed the parameter estimates from trajectory matching as starting values for the improved iterated filtering algorithm [14]. We iterated 100 particles 100 times for each set of starting values and the resulting parameter estimates among parameter sets with zero filtering failures are reported in Table S10.

Table S8: Ranges for starting parameters profiled in the epidemic phase.

Parameter	Range	Rationale	Reference
ρ	$[10^{-8}, 1]$	reporting proportion must exceed zero	
β_1	$[10^{-9}, 10]$	beta spline terms are dependent; for a given beta term, a value of 10 corresponds to a basic reproductive number (R0) of roughly 2.85	
ν	$[.95, 1]$	mixing coefficients in other model contexts are usually close to unity	[8, 9]
τ	$[1, 20]$	dispersion in the observation process likely exceeds that of a Poisson distribution if correctly specified	

Table S9: Parameters that were fixed across both model calibration stages.

Parameter	Value	Description	Reference
$N.0$	10,911,819	population of Haiti at the start of the study period	[15]
μ	.43	birth rate per 1000 population per week	[16]
δ	.14	natural death rate per 1000 population per week	[16]
$1/\sigma$	1.4	latent period (days)	[17]
$1/\gamma$	2	infectious period (days)	[18]
$1/\alpha$	8	mean duration of natural immunity (years)	range [19, 20]
θ_0	0	proportion of non-vaccinated Exposed individuals that develop Asymptomatic infection	assumed
κ	.95	proportion of reduction in transmissibility among Asymptomatic infections	assumed

Table S10: Parameters estimates or values for the epidemic phase of the data after model calibration.

Parameter	Calibrated	Median Estimate [Range]	Description
β_1	yes	5.8 [.04,12.1]	transmission parameter (1st component of basis spline)
β_2	yes	3.5 [0,10.3]	transmission parameter (2nd component of basis spline)
β_3	yes	4.4 [.18,9.7]	transmission parameter (3rd component of basis spline)
β_4	yes	2.8 [0,8.3]	transmission parameter (4th component of basis spline)
β_5	yes	6.2 [.8,12.2]	transmission parameter (5th component of basis spline)
β_6	yes	2.4 [0,7.7]	transmission parameter (6th component of basis spline)
ν	yes	.96 [.90,1.0]	population mixing coefficient (1 represents homogeneous mixing)
ρ	yes	.34 [.13,1]	reporting fraction
τ	yes	4.0 [1.7,5.9]	inverse dispersion for the negative binomial measurement model
$E.0$	yes	405 [1,1752]	initial number of Exposed individuals
$I.0$	yes	518 [1,2271]	initial number of Infected individuals

2.2.2 Endemic phase

After calibrating the epidemic phase of the data, we passed the final epidemic parameter estimates and state values (number of individuals in each compartment) at the final time point as starting parameters for the endemic model. The starting state values were fixed for each parameter set, but all other parameters calibrated in the epidemic phase were re-calibrated in the endemic phase with the iterated filtering algorithm (Table S11).

Table S11: Parameters estimates or values for the endemic phase of the data after model calibration.

Parameter	Calibrated	Median Estimate [Range]	Description
β_1	yes	5.4 [.06,80.4]	transmission parameter (1st component of basis spline)
β_2	yes	3.3 [0,36.2]	transmission parameter (2nd component of basis spline)
β_3	yes	4.3 [.07,63.1]	transmission parameter (3rd component of basis spline)
β_4	yes	3.5 [0,12.3]	transmission parameter (4th component of basis spline)
β_5	yes	5.0 [.3,98.8]	transmission parameter (5th component of basis spline)
β_6	yes	2.7 [0,9.7]	transmission parameter (6th component of basis spline)
ν	yes	.96 [.64,1.0]	population mixing coefficient (1 represents homogeneous mixing)
ρ	yes	.34 [.11,1]	reporting fraction
τ	yes	4.1 [2.1,7.0]	inverse dispersion for the negative binomial measurement model
$E.0$	no	248 [1,1693]	initial number of Exposed individuals, simulated from the epidemic model fits
$I.0$	no	324 [1,2160]	initial number of Infected individuals, simulated from the epidemic model fits

2.3 Assessment of Model Fit

We assessed the model fit visually over the entire time series and for the tail-end of the endemic phase due to its importance in realistic forward projections (Figure S7). While individual realizations simulated from a single parameter set retained substantial variability, summarizing the fits across multiple realizations and parameter sets presented a reasonable fit across both epidemic and endemic phases of the data. We also examined the reasonableness of all fitted parameter values (Figure S8 and Figure S9). In this examination, we note that estimates for reporting rate ρ are bimodal in both phases, with modes at 0.25 and near 1. We removed parameter sets in the endemic phase with very low log-likelihoods (log-likelihood ¡ -3000) because they corresponded to outlying calibrated parameter values. We used all remaining parameter sets to project the long-term effects of mass oral cholera vaccination campaigns in Haiti.

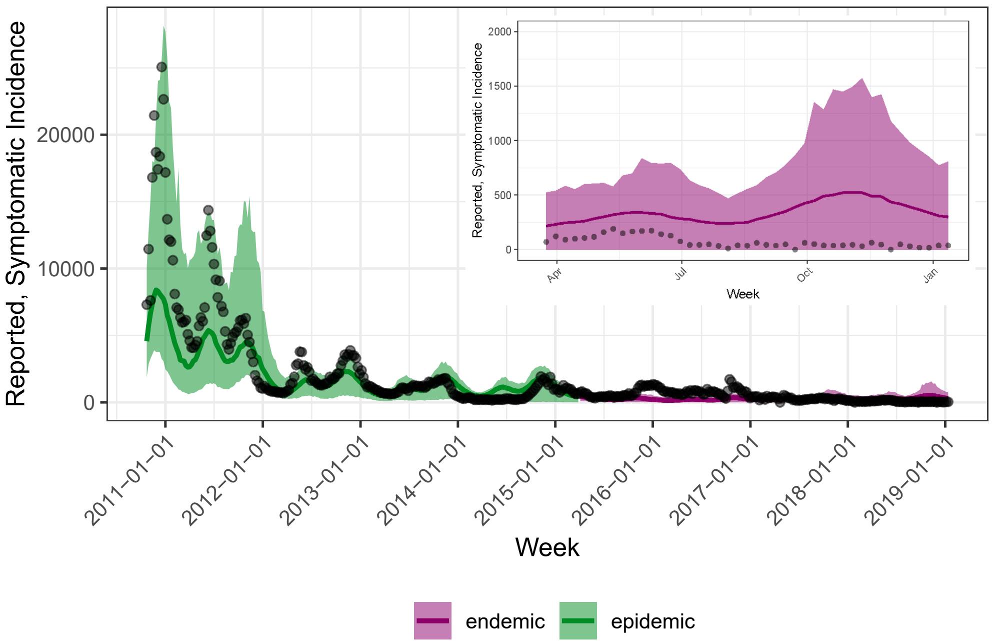

Figure S7: Model calibration of observed symptomatic cases for epidemic and endemic phases together. The inset represents the model calibration of observed symptomatic cases for the end of the endemic phase. Colored lines represent the median of the model calibrations across parameter sets and colored ribbons represent the range of model calibration values from the 2.5 to 97.5 percentile. Black dots represent Haiti cholera surveillance data.

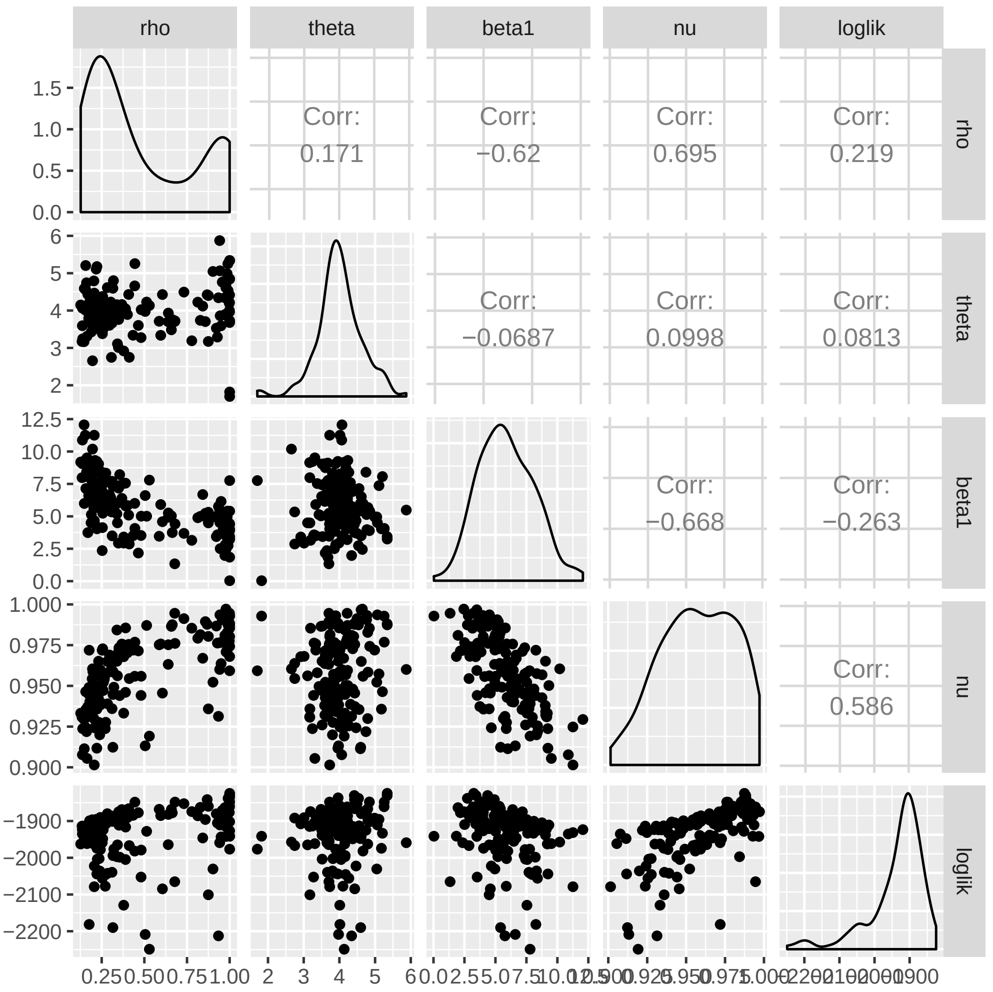

Figure S8: Distribution of parameter estimates and log likelihood for epidemic phase.

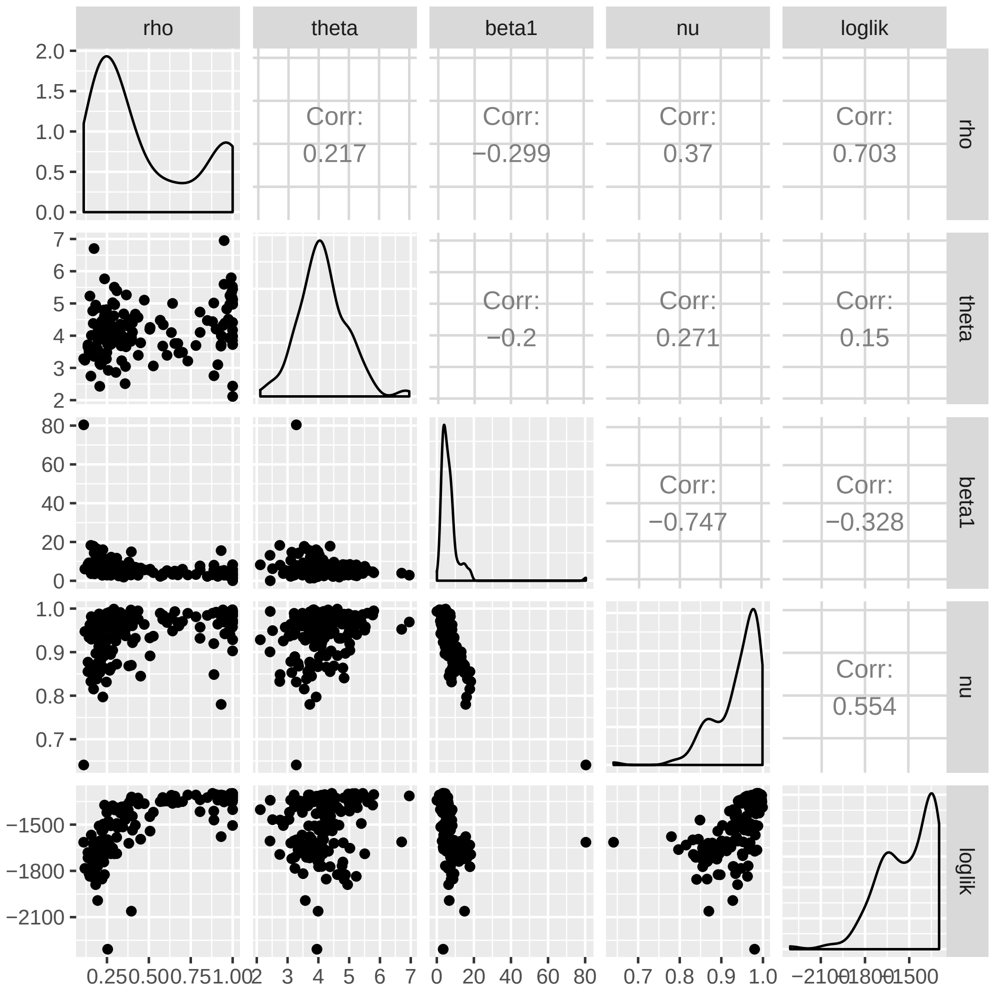

Figure S9: Distribution of parameter estimates and likelihood for endemic phase.

2.4 Simulation of Vaccination Campaigns

We used the estimated endemic phase parameters and the simulated states at the final time point to generate stochastic forecasts of observed and true cholera incidence in Haiti for 10 years. We compared model projections representing the status quo, where current control measures continue constantly over the 10 year forecast, to 36 vaccination scenarios that vary in their assumptions of the speed and geographic coverage of vaccination campaigns, levels of 2-dose and 1-dose vaccination coverage, and the efficacy of vaccine-induced immunity over time. Projections were simulated in POMP using an Euler approximation at day-long time intervals (delta.t = 1/7; models were calibrated at week-long time intervals). The observation process was stochastically simulated on top of the latent Markov process where the number of observed cases was assumed to follow a negative binomial distribution, as follows:

$$cases = NegBinom(\rho\xi, \tau)$$

2.4.1 Deployment of Vaccination Campaigns

Our model had only country-level disease dynamics, yet the pre-determined vaccination scenarios called for department-specific vaccination campaigns. As mentioned above, we simplified our deployment so that vaccination occurs in cohorts that represent vaccination of in a given department target, and all vaccines for a campaign are deployed in a

single week. These rollouts are evenly spaced in time over two or five year periods, as appropriate to the vaccination scenario, according to the schedule described in Figure S10 and Table S12.

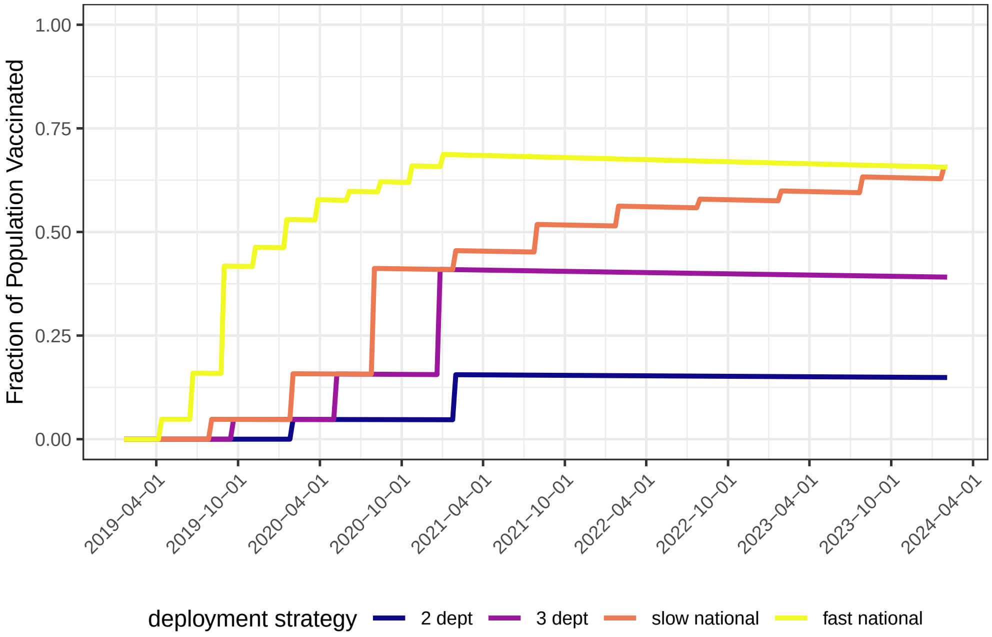

Figure S10: Influx of vaccines into the model projections, by vaccine deployment scenario. The fraction of the population vaccinated declines after the end of vaccination campaigns because the population is growing over time.

Table S12: Deployment schedule of vaccination campaigns in model projections.

Department	2 Dept	3 Dept	Slow National	Fast National
Centre	Feb 1, 2020	Sep 21, 2019	Aug 3, 2019	Apr 13, 2019
Artibonite	Jan 30, 2021	May 9, 2020	Jun 22, 2019	Feb 1, 2020
Ouest		Dec 26, 2020	Aug 31, 2019	Aug 1, 2020
Nord-Ouest			Nov 9, 2019	Jan 30, 2021
Nord			Jan 18, 2020	Jul 31, 2021
Sud			Mar 28, 2020	Jan 29, 2022
Nippes			Jun 6, 2020	Jul 30, 2022
Nord-Est			Aug 15, 2020	Jan 28, 2023
Sud-Est			Oct 24, 2020	Jul 29, 2023
Grand'Anse			Jan 2, 2021	Jan 27, 2024

2.5 Calculating Elimination Measures

We assessed the probability of elimination at three, five, and ten years after the start of the vaccination campaign. Elimination is defined as achieving a true incidence below one case for at least 52 consecutive weeks and remaining at this level through to the end of the 10 year projection period. The probability of elimination at a given time point is the proportion of simulations that achieved the start of an elimination state before the time point of interest (e.g., began the 52 consecutive weeks below one case before three years after vaccination start). True incidence includes all symptomatic and asymptomatic cases; this is different than the observed, symptomatic cases, which were the subset of data that had been calibrated to the Haiti surveillance data.

Among simulations that achieved elimination, we calculated the median time to elimination, which is the median number of weeks between the start of vaccination and elimination across simulations.

Model-2 Supplement

Laura Matrajt, Jonathan D. Sugimoto, M. Elizabeth Halloran, and Ira M. Longini Jr.
Fred Hutchinson Cancer Research Center and University of Florida

3.1 Model Description

We constructed a deterministic meta-population model to simulate cholera transmission and vaccination in Haiti. Our model consists of a network of ten nodes representing the ten administrative departments of Haiti. For each department, susceptible individuals S_i get infected upon contact with an infected person (at a rate β) or with a water source (at a rate β_W, modulated by a sinusoidal function representing the dry and rainy season). Upon infection, individuals become exposed E_i (infected but not yet infectious) for $1/\gamma_E$ weeks. A proportion k of these individuals will develop symptoms and transitions to the infectious symptomatic (I_i) compartment. These individuals shed bacteria into the environment at a rate μ. The remaining $1-k$ infectious individuals are assumed to be asymtomatic (A_i), and are assumed to be significantly less infectious and shed significantly less cholera in the environment (represented by multipliers red_β and red_μ respectively). Infected individuals remain infectious for a period of $1/\gamma$ weeks after which they are assumed to be fully recovered and to have transient natural immunity to cholera infections. Natural immunity (both for symptomatic and asymptomatic infections) is assumed to last $1/\sigma$ weeks, after which recovered individuals become fully susceptible to infection again.

In addition, our model tracks the amount of bacteria present in a water reservoir W_i. Bacteria present in water decays at a rate δ. Finally, departments are connected at two levels: through a network of major highways, representing the flow of people in the country, and through a network of rivers, representing the flow of water (see figure S14). A full description of the human mobility network and water mobility network is given below.

Vaccination: We simulate vaccination with one or two doses of Oral Cholera Vaccine (OCV), by explicitly adding vaccination compartments (for one or two doses) to our model. We have the following nomenclature for the vaccination compartments: (V_{ij}, E_{ij}, I_{ij}, A_{ij}, R_{ij} and RA_{ij} for vaccinated susceptible, exposed, infectious symptomatic or asymptomatic and recovered symptomatic or asymptomatic. The index i ($i \in \{1, ..10\}$) represents the department and $j = \{1, 2\}$ represents vaccination with one or two doses respectively. For bookkeeping purposes, we further separated vaccinated individuals who are younger than five years old in different compartments, where we assumed that the proportion of children under five years old would stay constant over the whole simulation period, but we do not explicitly model aging. Hence, V_{ij_5}, E_{ij_5}, I_{ij_5}, A_{ij_5}, R_{ij_5} and RA_{ij_5} represent the analogous vaccination compartments for children under five years old. Vaccine protection is assumed to be "leaky" [21], so that vaccinated individuals see their force of infection reduced by θ_j ($j = 1, 2$ represents vaccination with one or two doses respectively and θ_{j_5} represents vaccination with one or two doses for children under five years old). Vaccination was implemented in weekly pulses, and vaccinated individuals are assumed to have immediate protection with those vaccinated with a single dose being protected for one year, while those fully vaccinated being protected for five years [6]. For example, to model a vaccination campaign with 80% coverage in all departments over a two-year period, we first computed the total number of doses to be given in a single week, and then modeled a vaccination campaign where each week that number of people would be vaccinated in a single department instantaneously. We vaccinated departments in order according to the 2018 reported cholera incidence, with those with the highest incidence being vaccinated first. Figure S11 provides the fraction of the total population vaccinated each week for each vaccination scenario (see the meta supplement for full descriptions of each vaccination scenario). We modeled a conservative scenario where only susceptible individuals will gain protection from the vaccine, so that recovered, infected or latent individuals do not benefit in the model from any vaccine protection. Vaccine efficacy was derived from [6]. Because this is a deterministic model and vaccination was administered weekly, we could not model the vaccine efficacy in a dynamic way, instead, we utilized the median

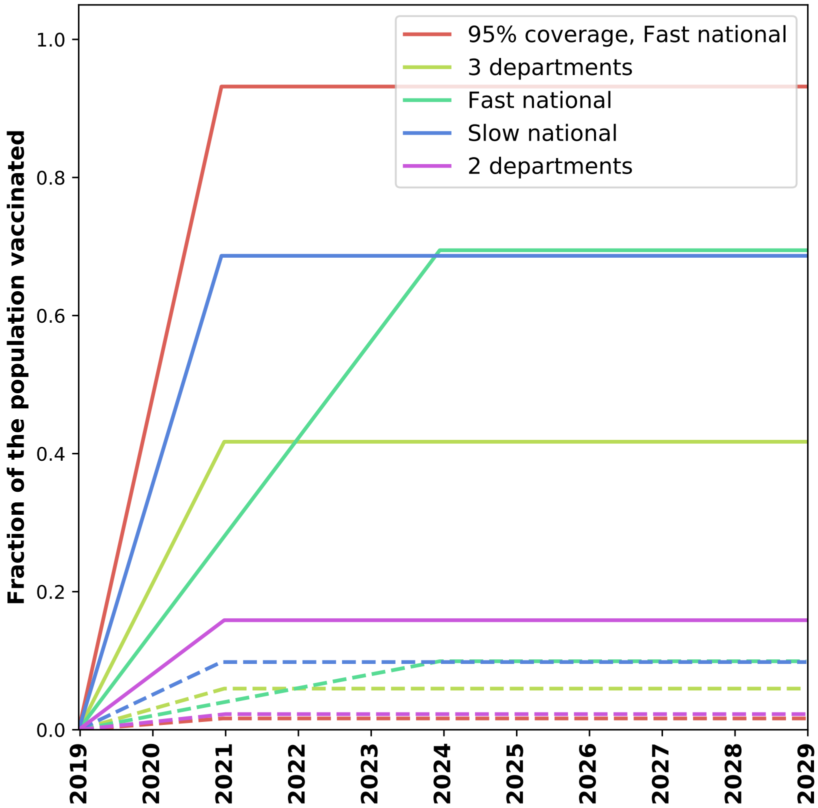

Figure S11: Fraction of the population vaccinated for each scenario considered with two doses (solid lines) or one dose (dashed lines). A full description of the vaccination scearios can be found in the meta supplement.

over five years (two doses) or over one year (one dose) of the log-linear weighted regression model presented in the main supplement. This resulted in $VE_2 = 51.9\%$ for two doses and $VE_1 = 42.9\%$ for a single dose. OCV was assumed to be less effective ($0.4688 * VE_{1,2}$) in children under five. Table S13 shows all the parameters and the values used in the model.

Network structure: Political boundaries, road networks, and river systems shapefiles and an interpolated population-density surface were obtained from the dataverse website maintained by Harvard University [22]. An elevation surface raster was also obtained and processed from [23]. We generated a matrix D describing the average distance that individuals would be expected to travel by major road between each pair of departments as follows. We overlaid the political boundary shapefile on the interpolated population-density raster and we calculated the population-density weighted centroid for each department. Each weighted centroid was then related to nearest point along the road network shapefile, which allowed us to calculate the distance (in km) along the shortest interdepartmental path across primary, primary link, secondary, or secondary link highways. Finally, we used a standard gravity model [24] to describe the movement of people through the network, hence obtaining the matrix T used in the equations below, with

$$T_{ij} = v_{rate} * \frac{Pop_i * Pop_j}{D_{ij}^2}$$

where Pop_i is the total population for department i and v_{rate} is a free parameter.

As rivers and streams typically flow in one direction, the matrix characterizing the potential fluvial flow of free-living V. cholerae between a given pair of departments will not necessarily be symmetric. To generate this matrix, we

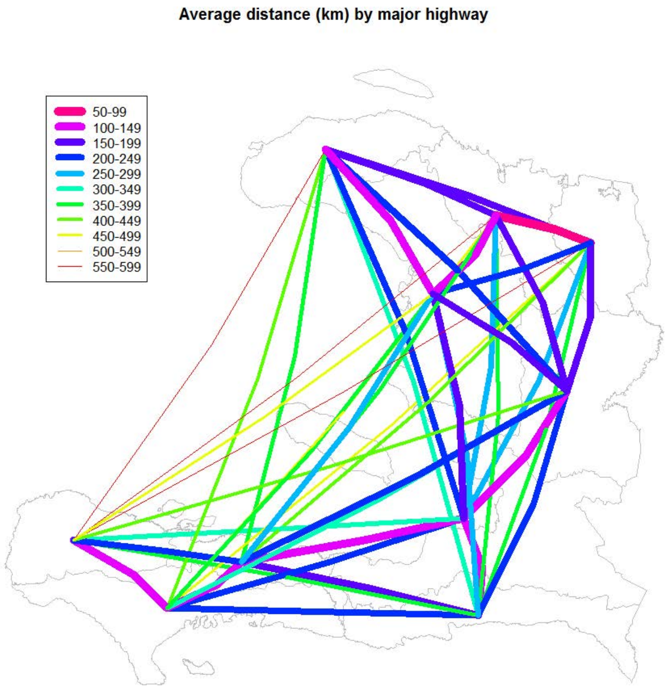

Figure S12: Schematic representation of the human mobility network used in the model. Lines between two departments represent the distance (kilometers) by major highway between the geographic centers of each pair. Line thickness is inversely proportional to distance between departments, so that lines between the closest departments are the thickest, representing the departments the most connected via human mobility.

used the elevation raster to determine the direction of flow for each segment of river and the total loss in elevation along its length. Since contamination of the river water depends upon the deposition of untreated human waste, we also used population density surface to calculate the average population density along the banks of each segment of river. Using this information we calculated an index of potential exposure for each segment of river equal to its length in meters multiplied by the average population density along its length and divided by the amount of elevation (meters) loss. This index should take on higher values where more people are present along the banks of a river segment or where the river segment is longer, and it should decrease in value where the grade is steeper (i.e., the river is likely flowing faster and more likely to carry V. cholerae away faster). To generate the final matrix WMat describing the department to department flow of river water, we sum up the potential exposure index values for all segments of any river that flows out of department A and into department B, even if a river flows through another department in between. Where one river flows into another river, the segments of the second river downstream from the junction are considered to inherit the potential exposure index level of all segments of both rivers that are upstream from the junction. Figure S13 provides a visual representation of the water matrix obtained through this procedure.

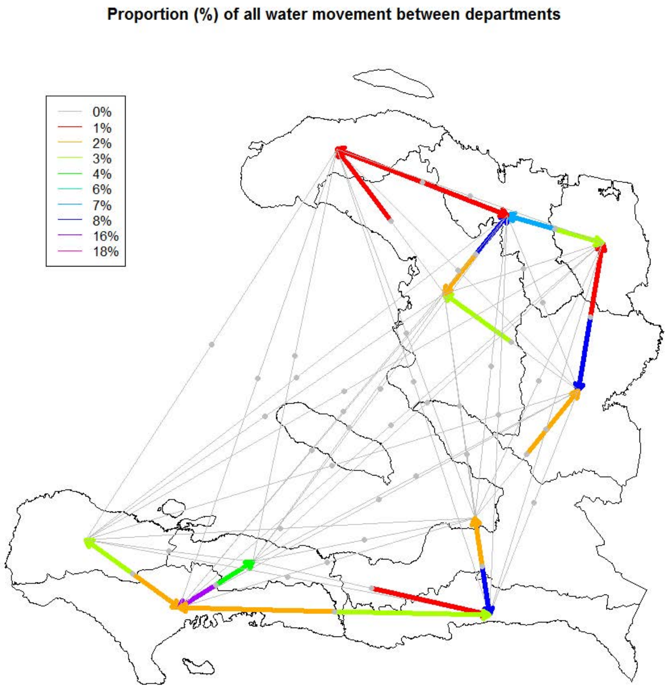

Figure S13: Schematic representation of the water network used in the model. Each arrow represents the proportion of all inter-departmental river water flow that occurs between each pair of departments. The asymmetric nature of the flow is depicted as arrows pointing toward the receiving department with the midpoint (gray circles along lines) of the line between each pair of departments as the beginning of each arrow. Gray lines indicate a zero level of water flow.

With all these assumptions, we obtain the following system of differential equations for each department (index i has been omitted from the equations for simplicity, except in the transport terms where the indices are left for clarity):

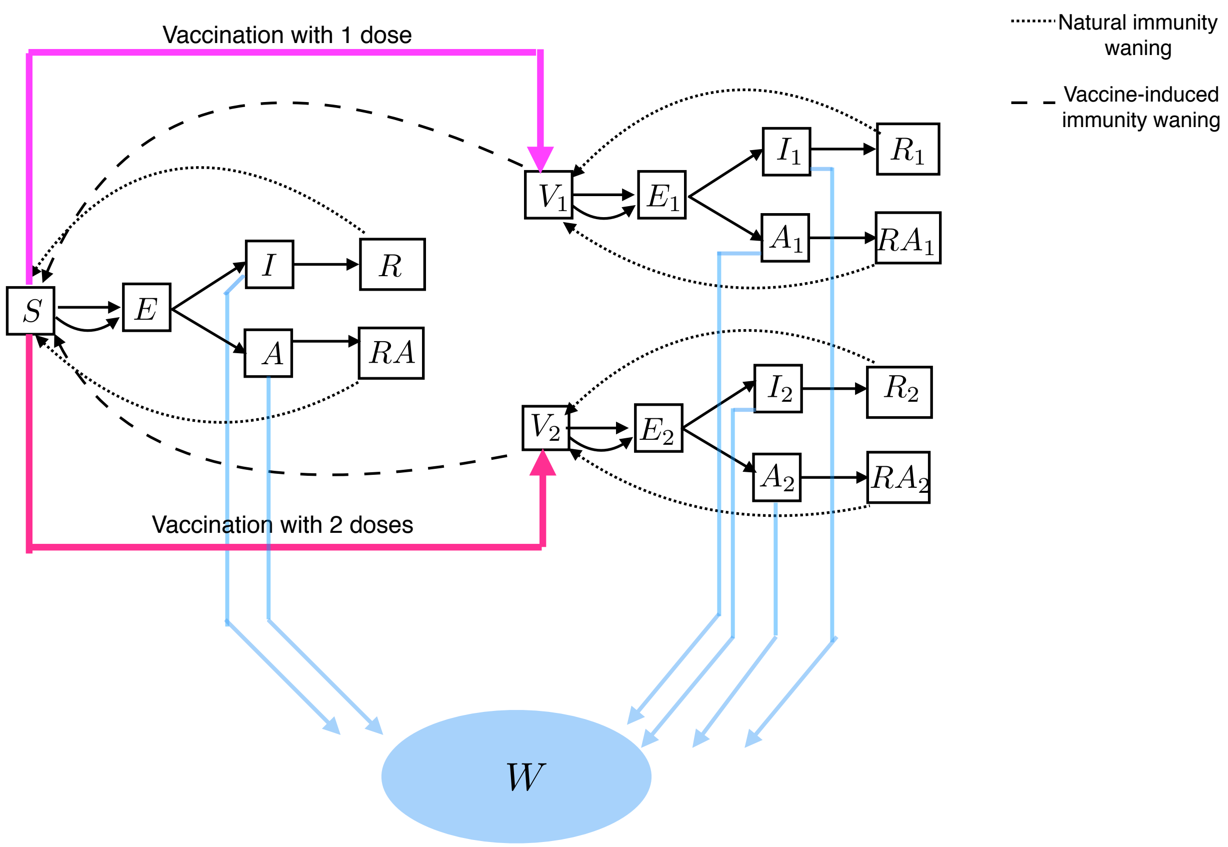

Figure S14: Schematic diagram of the cholera transmission and vaccination dynamics in a single department.

Unvaccinated individuals:

$$\frac{dS}{dt} = -\lambda S + \sigma(R + R_A) + \omega_1(V_1 + V_{1_5}) + \omega_2(V_2 + V_{2_5}) + \left\{ \sum_{j=1}^{n} T_{ji} S_j - \sum_{j=1}^{n} T_{ij} S_i \right\}$$

$$\frac{dE}{dt} = \lambda S - \gamma_E E + \left\{ \sum_{j=1}^{n} T_{ji} E_j - \sum_{j=1}^{n} T_{ij} E_i \right\}$$

$$\frac{dI}{dt} = k\gamma_E E - (\gamma) I + \left\{ \sum_{j=1}^{n} T_{ji} I_j - \sum_{j=1}^{n} T_{ij} I_i \right\}$$

$$\frac{dA}{dt} = (1-k)\gamma_E E - \gamma A + \left\{ \sum_{j=1}^{n} T_{ji} A_j - \sum_{j=1}^{n} T_{ij} A_i \right\}$$

$$\frac{dR}{dt} = \gamma I - \sigma R + \left\{ \sum_{j=1}^{n} T_{ji} R_j - \sum_{j=1}^{n} T_{ij} R_i \right\}$$

$$\frac{dR_A}{dt} = \gamma A - \sigma R_A + \left\{ \sum_{j=1}^{n} T_{ji} R_{Aj} - \sum_{j=1}^{n} T_{ij} R_{Ai} \right\}$$

Vaccinated individuals:

Vaccinated with one dose over 5:

$$\frac{dV_1}{dt} = -\theta_1(\lambda V_1) + \sigma(R_1 + R_{A1}) - \omega_1 V_1 + \Big\{ \sum_{j=1}^{n} T_{ji} V_{1j} - \sum_{j=1}^{n} T_{ij} V_{1i} \Big\}$$

$$\frac{dE_1}{dt} = \theta_1(\lambda V_1) - \gamma_E E_1 + \Big\{ \sum_{j=1}^{n} T_{ji} E_{1j} - \sum_{j=1}^{n} T_{ij} E_{1i} \Big\}$$

$$\frac{dI_1}{dt} = k\gamma_E E_1 - \gamma I_1 + \Big\{ \sum_{j=1}^{n} T_{ji} I_{1j} - \sum_{j=1}^{n} T_{ij} I_{1i} \Big\}$$

$$\frac{dA_1}{dt} = (1-k)\gamma_E E_1 - \gamma A_1 + \Big\{ \sum_{j=1}^{n} T_{ji} A_{1j} - \sum_{j=1}^{n} T_{ij} A_{1i} \Big\}$$

$$\frac{dR_1}{dt} = \gamma I1 - \sigma R_1 + \Big\{ \sum_{j=1}^{n} T_{ji} R_{1j} - \sum_{j=1}^{n} T_{ij} R_{1i} \Big\}$$

$$\frac{dR_{A1}}{dt} = \gamma A_1 - \sigma R_{A1} + \Big\{ \sum_{j=1}^{n} T_{ji} R_{A1j} - \sum_{j=1}^{n} T_{ij} R_{A1i} \Big\}$$

Vaccinated with one dose under 5:

$$\frac{dV_{1_5}}{dt} = -\theta_{1_5}(\lambda V_{1_5}) + \sigma(R_{1_5} + R_{A1_5}) - \omega_1 V_{1_5} + \Big\{ \sum_{j=1}^{n} T_{ji} V_{1_5 j} - \sum_{j=1}^{n} T_{ij} V_{1_5 i} \Big\}$$

$$\frac{dE_{1_5}}{dt} = \theta_{1_5}(\lambda V_{1_5}) - \gamma_E E_{1_5} + \Big\{ \sum_{j=1}^{n} T_{ji} E_{1_5 j} - \sum_{j=1}^{n} T_{ij} E_{1_5 i} \Big\}$$

$$\frac{dI_{1_5}}{dt} = k\gamma_E E_{1_5} - \gamma I_{1_5} + \Big\{ \sum_{j=1}^{n} T_{ji} I_{1_5 j} - \sum_{j=1}^{n} T_{ij} I_{1_5 i} \Big\}$$

$$\frac{dA_{1_5}}{dt} = (1-k)\gamma_E E_{1_5} - \gamma A_{1_5} + \Big\{ \sum_{j=1}^{n} T_{ji} A_{1_5 j} - \sum_{j=1}^{n} T_{ij} A_{1_5 i} \Big\}$$

$$\frac{dR_{1_5}}{dt} = \gamma I_{1_5} - \sigma R_{1_5} + \Big\{ \sum_{j=1}^{n} T_{ji} R_{1_5 j} - \sum_{j=1}^{n} T_{ij} R_{1_5 i} \Big\}$$

$$\frac{dR_{A1_5}}{dt} = \gamma A_{1_5} - \sigma R_{A1_5} + \Big\{ \sum_{j=1}^{n} T_{ji} R_{A1_5 j} - \sum_{j=1}^{n} T_{ij} R_{A1_5 i} \Big\}$$

Vaccinated with two doses over 5:

$$\frac{dV_2}{dt} = -\theta_2(\lambda V_2) + \sigma(R_2 + R_{A2}) - \omega_2 V_2 + \Big\{\sum_{j=1}^{n} T_{ji}V_{2j} - \sum_{j=1}^{n} T_{ij}V_{2i}\Big\}$$

$$\frac{dE_2}{dt} = \theta_2(\lambda V_2) - \gamma_E E_2 + \Big\{\sum_{j=1}^{n} T_{ji}E_{2j} - \sum_{j=1}^{n} T_{ij}E_{2i}\Big\}$$

$$\frac{dI_2}{dt} = k\gamma_E E_2 - \gamma I_2 + \Big\{\sum_{j=1}^{n} T_{ji}I_{2j} - \sum_{j=1}^{n} T_{ij}I_{2i}\Big\}$$

$$\frac{dA_2}{dt} = (1-k)\gamma_E E_2 - \gamma A_2 + \Big\{\sum_{j=1}^{n} T_{ji}A_{2j} - \sum_{j=1}^{n} T_{ij}A_{2i}\Big\}$$

$$\frac{dR_2}{dt} = \gamma I_2 - \sigma R_2 + \Big\{\sum_{j=1}^{n} T_{ji}R_{2j} - \sum_{j=1}^{n} T_{ij}R_{2i}\Big\}$$

$$\frac{dR_{A2}}{dt} = \gamma A_2 - \sigma R_{A2i} + \Big\{\sum_{j=1}^{n} T_{ji}R_{A2j} - \sum_{j=1}^{n} T_{ij}R_{A2i}\Big\}$$

Vaccinated with two doses under 5:

$$\frac{dV_{2_5}}{dt} = -\theta_{2_5}(\lambda V_{2_5}) + \sigma(R_{2_5} + R_{A2_5}) - \omega_2 V_{2_5} + \Big\{\sum_{j=1}^{n} T_{ji}V_{2_5 j} - \sum_{j=1}^{n} T_{ij}V_{2_5 i}\Big\}$$

$$\frac{dE_{2_5}}{dt} = \theta_{2_5}(\lambda V_{2_5}) - \gamma_E E_{2_5} + \Big\{\sum_{j=1}^{n} T_{ji}E_{2_5 j} - \sum_{j=1}^{n} T_{ij}E_{2_5 i}\Big\}$$

$$\frac{dI_{2_5}}{dt} = k\gamma_E E_{2_5} - \gamma I_{2_5} + \Big\{\sum_{j=1}^{n} T_{ji}I_{2_5 j} - \sum_{j=1}^{n} T_{ij}I_{2_5 i}\Big\}$$

$$\frac{dA_{2_5}}{dt} = (1-k)\gamma_E E_{2_5} - \gamma A_{2_5} + \Big\{\sum_{j=1}^{n} T_{ji}A_{2_5 j} - \sum_{j=1}^{n} T_{ij}A_{2_5 i}\Big\}$$

$$\frac{dR_{2_5}}{dt} = \gamma I_{2_5} - \sigma R_{2_5} + \Big\{\sum_{j=1}^{n} T_{ji}R_{2_5 j} - \sum_{j=1}^{n} T_{ij}R_{2_5 i}\Big\}$$

$$\frac{dR_{A2_5}}{dt} = \gamma A_{2_5} - \sigma R_{A2_5} + \Big\{\sum_{j=1}^{n} T_{ji}R_{A2_5 j} - \sum_{j=1}^{n} T_{ij}R_{A2_5 i}\Big\}$$

Water equations:

$$\frac{dW}{dt} = (\mu(I + I_1 + I_2 + I_{1_5} + I_{2_5}) + \mu_A(A + A_1 + A_2 + A_{1_5} + A_{2_5})) - \delta W$$
$$+ w_r\Big\{\sum_{j=1}^{n}(\text{WMat}_{ji}W_j - \text{WMat}_{ij}W_i)\Big\}$$

where $\theta_1 = 1 - VE_1$ and $\theta_2 = 1 - VE_2$. In addition, T is the travel matrix representing travel through major highways and WMat is the river matrix representing the flow of water through Haiti. We assumed that $\beta_A = red_\beta\beta$ and $\mu_A = red_\mu\mu$, that is, asymptomatic individuals contribute to the infection process at a much lower rate in two ways: first, their rate of infection to other people (person-to-person infection cycle) is reduced and they shed less bacteria into the environment. In this exercise, we assumed that asymptomatic people are 1000 times less infectious to others and shed 10^7 times less bacteria than a symptomatic infected person. With this, the force of infection λ is given by:

$$\lambda = 0.5\big(1+\alpha_s\cos(2\pi\frac{t}{p_s})\big)\frac{\beta_W W}{Sat+W} + \big\{\beta(I+I_1+I_2+I_{1_5}+I_{2_5}) + \beta_A(A+A_1+A_2+A_{1_5}+A_{2_5})\big\}$$

Table S13 provides a list of the parameters used and indicates whether they were fit to the data or assumed.

Table S13: Parameters definitions and values.

Model parameter	Symbol	Value	Units	Reference
Decay of cholera in water	δ	$1/3$	weeks^{-1}	mean of range given in [25]
Fraction of symptomatic infections	k	0.2	–	[26, 27]
Infectious period	$1/\gamma$	1	weeks	mean of range given in[25]
Latent period	$1/\gamma_E$	0.18	weeks	mean of range given in [25]
Relative infectiousness of asymptomatic infections	red_β	10^{-3}	–	[28]
Relative shedding of cholera for asymptomatic infections	red_μ	10^{-7}	–	[28]
Duration of natural immunity	$1/\sigma$	5 [a]	years	–
Concentration of *V. cholerae* at which the infection rate is 50% of the maximum infection rate	Sat	10^5	cells/ml	mean of range given in [25]
Amplitude of seasonal forcing	α_s	0.4 [b]	–	–
Period of seasonal forcing	p_s	52[c]	weeks	–
Person-to-person infection rate	β	$9.9*10^{-7}$	week^{-1}	fit
Environmental infection rate	β_W	$4.03*10^{-2}$		fit
Symptomatic shedding rate	μ	$3.98*10^3$, 2.58		fit
Fraction of susceptible population in 03/2014	f	0.75	–	
Travel matrix multiplier	v_{rate}	10^{-12} [d]	–	
Rate of loss of vaccine-induced immunity for one dose	ω_1	1	years^{-1}	[6]
Rate of loss of vaccine-induced immunity for two doses	ω_2	$1/5$	years^{-1}	[6]
Vaccine efficacy for one dose	VE_1	varied		see text
Vaccine efficacy for two doses	VE_2	varied		see text
Reporting rate (symptomatic infections)	ρ	0.2	–	[29]

[a] Natural immunity was assumed to last as long as vaccine-induced immunity.
[b] α_s was chosen to mimic the observed cholera seasonal patterns in Haiti
[c] p_s was chosen to mimic the observed cholera seasonal patterns in Haiti
[d] v_rate was chosen to mimic the distribution of infections in Haiti

3.2 Model Fitting/Calibration

We fit our model to the weekly reported cases provided by Haiti MSPP in two stages. First, we utilized reported cases from 10/2010 to 03/2014 and used Ordinary Least Squares (OLS, leastsq function from optimize package in Scipy, python [30]) to fit weekly incidence provided by the model to weekly reported cases. We assumed that only 20% of cholera infections become symptomatic [26, 27] and out of those, only 20% would be reported [29]. All model parameters were fixed except for three parameters: the person-to-person infection rate, the environmental infection rate, and the symptomatic shedding rate (β, β_W and μ). When possible, fixed parameters were directly taken from data, or they were taken as averages of known ranges (see Table S13). The time period utilized in this first stage corresponds to first phase of the explosive cholera epidemic in Haiti, which occurred after the introduction of cholera in Haiti. We ran our OLS algorithm with 1000 different random starting points and used the mean of the resulting best parameter sets to run the simulations for our "best fit" (see figure S15A). We then focus on the endemic dynamics of cholera transmission, going from 03/2014 to 01/2019. There were several interventions against cholera in Haiti during this period, including vaccination campaigns, WASH interventions and rapid response teams [31], all of which might have altered the course of the cholera dynamics. Hence, we re-fit our model for this part, but we only re-fit the parameter μ while keeping the infection rates (β and β_W) constant. We could have chosen any of these three

parameters to refit our data. The parameter μ was chosen as a way to represent that most of these interventions might have decreased the rate at which symptomatic infected people would have shed cholera in the environment.

In a serosurvey conducted by Jackson *et al* [26], it was found that 39% of the population were seropositive for V. cholerae antibody tests. However, only 28% and 23% of the population were positive for specific antibody tests for the Ogawa and Inaba strains respectively. The study was conducted in the Grande Saline Commune, which was particularly hit by the cholera epidemic during 2010 and 2011. Based on these numbers, we performed sensitivity analysis and found that our model fit the data best when we assumed only 25% of the population was immune to cholera. Hence, we assumed that 75% of the population was susceptible to cholera on March 2014 (see figure S15B).

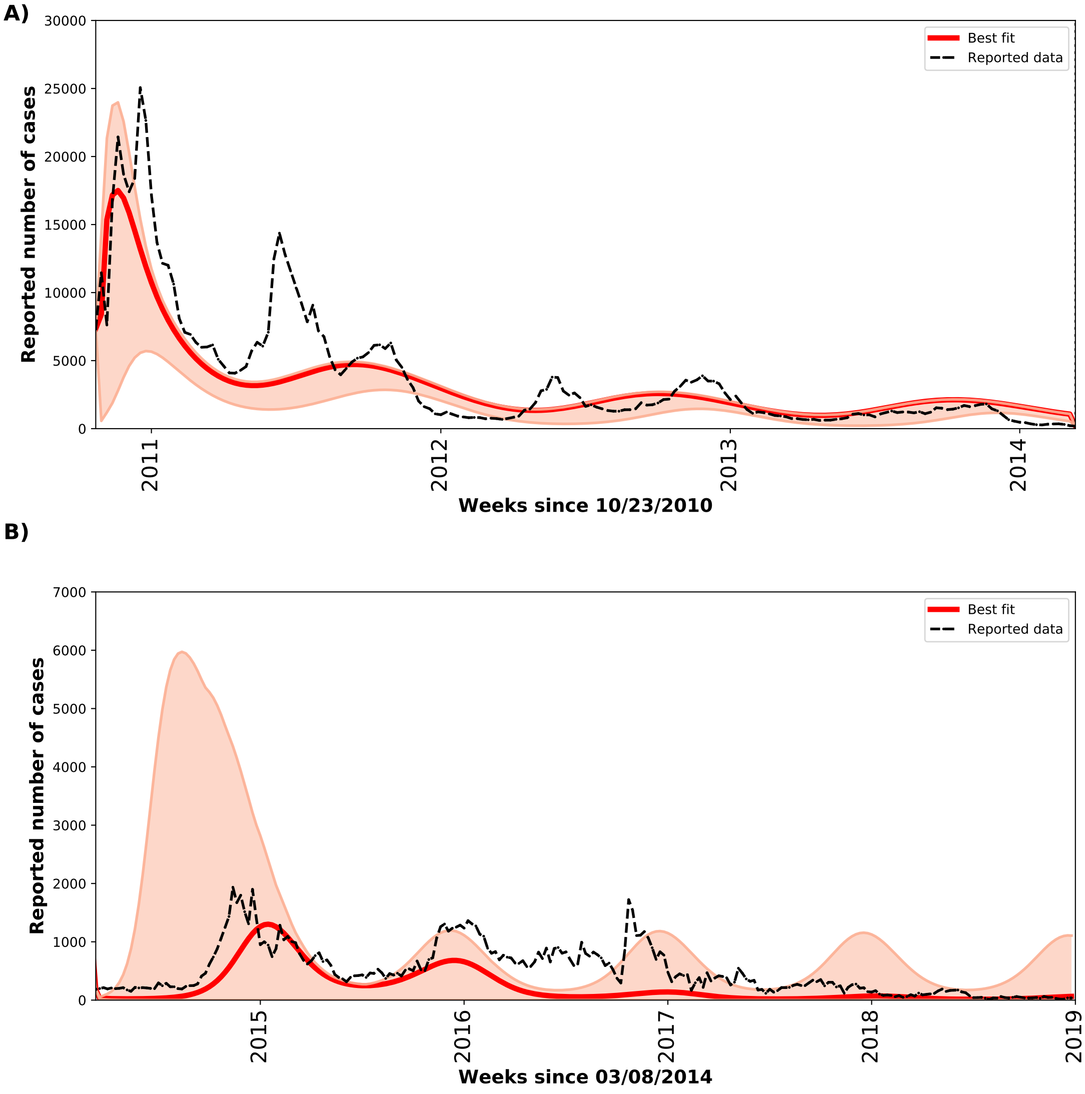

Figure S15: Model fit (red line) and reported number of cases (black dashed line) for the cholera epidemic in Haiti. Case reporting started on October 22^{n}d, 2010. The light orange area represents the range in the number of cases generated by the sensitivity analysis.

3.2.1 Assessment of Model Fit

To assess the model fit we performed extensive sensitivity analysis. We used an interval centered around each fit parameter (we used 25% of the parameter's value as the 1/2 width of the interval) and around the fraction susceptible in March 2014 (we used 10% of this value as the 1/2 width of the sampling interval) to obtain a hyper cube around our fit values. We then used a Latin hyper-cube sampling scheme to obtain 1000 parameter sets. These parameter sets were used to run all the vaccination scenarios, and were used to compute the lower and upper bounds presented in the main results.

3.2.2 Computation of summary statistics

In order to compare the results from the four modeling groups, summary statistics were pre-determined to be used by all groups (see main supplement for details). Since this is a deterministic model, these statistics were computed by using the runs from the 1000 parameter sets that were obtained above. Mainly, the mean and upper and lower bounds were computed directly from the runs. The probability of elimination was computed as the fraction of runs that had less than one infection for 52 consecutive weeks.

Code available at https://github.com/lulelita/HaitiCholeraMultiModelingProject.

Model-3 Supplement

Joseph Lemaitre, Damiano Pasetto, Javier Perez-Saez, Flavio Finger, and Andrea Rinaldo
Laboratory of Ecohydrology, Ecole Polytechnique Fédérale de Lausanne (CH)

4.1 Model Description

General Principles The cholera model we adopted to study the Haitian epidemic is a stochastic compartmental model applied at the level of the ten Haitian departments. It is the stochastic translation of a deterministic SIRB model based on Ordinary Differential Equations (ODEs) which has been extensively used to simulate the Haitian cholera epidemic in previous studies [1, 32, 33, 34, 35]. Here the model has been implemented as a discrete-state model based on a Partially-Observed Markov Process (POMP), simulating the stochastic transitions between compartments as discrete events[36].

The model subdivides the population of each department into compartments counting the number of individuals at the different stages of the disease: susceptible individuals (S), symptomatic (I) and asymptomatic (A) infected and recovered individuals (R). The main feature of our model is that it contains an environmental compartment describing the bacterial concentration (B) in the local environment, which is used to estimate the force of infection [1, 32]. Precipitation has been shown to be an important environmental driver of cholera transmission [37], especially in Haiti [1]. In our model, it increases the rate at which bacteria shed by infected individuals enter the environmental reservoir and thus increases the bacterial concentration and finally the force of infection [35]. A diagram of the model is given in Fig. S16.

4.1.1 Model Dynamics

The following dynamics characterize the model (see Figure S16):

Infection The force of infection governs the rate at which susceptible individuals become infected. A proportion σ become symptomatically infected, and the rest asymptomatically infected.

Force of Infection and mobility The force of infection in each department contains an additional term representing the number of cholera cases in the rest of Haiti. This allows for a possible introduction of cholera due to human mobility between departments. The force of infection in each department is composed of two parts. The first is related to the local bacterial concentration of the department. The second is related to case importation from other departments through human-to-human transmission. The corresponding equation for the ith department reads:

$$F_0^i(t) = \beta^i \frac{B_i(t)}{1 + B_i(t)} + c^i \sum_{j \neq i} (I_j(t) + A_j(t)).$$

The first term in the sum represents local transmission governed by the department-specific exposure parameter β^i which multiplies the logistic dose-response of the rescaled local bacterial concentration $B = B^*/K$, where B^* is the unscaled concentration of vibrios and K the half-saturation constant of the logistic function $\frac{B_i^*(t)}{K+B_i^*(t)}$. Case importation from other departments is given by the sum of the asymptomatically and symptomatically infected in department j, modulated by a parameter c^i which represents the intensity of case introduction from other departments in Haiti to department i.

Shedding Symptomatically and asymptomatically infected individuals shed bacteria. The shedding rate of asymptomatics, θ_A, is modeled as a fraction of the shedding rate of symptomatic individuals θ_I [38].

Recovery rate The recovery rate is the same for both asymptomatic and symptomatic individuals ($\gamma_I = \gamma_A = 0.2$ d^{-1}, [39, 40]).

Acquired immunity Individuals acquire natural immunity and remain in the recovered compartment (R) for a period that lasts for $1/\rho = 8$ years on average, before reintegrating the susceptible compartment.

Gamma-distributed immunity loss To better approximate the gamma distribution that typically characterizes the duration of immunity [36], recovered individuals pass through a succession of 3 separate recovered compartments (R_1, R_2, R_3) characterized by the same transition rate $\rho_1 = \rho_2 = \rho_3 = 3\rho$.

Bacterial Dynamics The size of the bacterial reservoir is proportional to the population density D_i of the department. Bacteria die at rate μ_B. Rainfall influences the bacteria concentration by increasing the rate at which bacteria enter the environmental reservoir.

Measurement Process The reported cases are modelled by a negative-binomial distribution with dispersion parameter p. We accounts for over- or under-reporting through the reporting parameter ϵ.

Stochasticity Overdispersion in the infection process is introduced by multiplying the force of infection F_0 by a time-continuous white noise process $\xi(t)$ defined as the differentiation of an integrated noise process $\xi(t) = \frac{d}{dt}\Gamma(t)$, here taken to be have a Gamma distribution with mean Δt and variance $\sigma^2 \Delta t$ [41]:

$$\xi(t) = \Gamma(t + \Delta t) - \Gamma(t) \sim \text{Gamma}\left(\frac{\Delta t}{\sigma^2}, \sigma^2\right).$$

Since $\xi(t)$ is non-negative it can serve as a multiplicative noise on the force of infection:

$$F_i(t) = F_0^i(t)\xi(t),$$

which yields to over-dispersion in the transitions.

Vaccination dynamics At each vaccination campaign, the available vaccine doses are uniformly distributed among susceptible (S), asymptomatic infected (A) and recovered (R_1, R_2, R_3) individuals. The rate of vaccination is indicated with r_V. Individuals can receive either one or two doses of OCV, which yield respective efficacies of $\eta_{1d}(t)$ and $\eta_{2d}(t)$, as defined in the main text. There is no age structure but the efficacy is set to be the population-weighted average of estimated efficacy for those under 5 years old and those over 5 years old. The model considers ten additional compartments for each vaccination campaign, in order to distinguish among individuals who received one (compartments V_{1d}^S, V_{1d}^A, $V_{1d}^{R_k}$, k =1, 2, 3) or two (compartments V_{2d}^S, V_{2d}^A, $V_{2d}^{R_k}$, k =1, 2, 3) doses of OCV. Vaccinated susceptible individuals (V_{1d}^S and V_{2d}^S) have a lower probability to become infected (and thus entering classes I or A) than non-vaccinated susceptibles. This is modeled through the multiplicative reduction of the force of infection by a factor $(1 - \eta_{1d}(t))$ or $(1 - \eta_{2d}(t))$ respectively. The vaccination campaign window is split equally between departments (i.e for a vaccination campaign of 5 years duration, each department will be vaccinated during a 6 month period). Vaccine efficacy starts waning after the first half of the duration of the department's vaccination campaign. For example, if for department i the vaccination campaign j spans from $t_a^{i,j}$ to $t_b^{i,j}$, then:

$$\eta^{i,j}(t) = \begin{cases} \eta_0(0) & \text{if t} < t_a^{i,j} + \frac{t_b^{i,j} - t_a^{i,j}}{2} \\ \eta_0(t - (t_a^{i,j} + \frac{t_b^{i,j} - t_a^{i,j}}{2})) & \text{if t} > t_a^{i,j} + \frac{t_b^{i,j} - t_a^{i,j}}{2} \end{cases} \tag{4.1}$$

where $\eta_0(t)$ is the scenario dependant vaccine efficacy as defined in the meta-supplement. The rates at which individuals leave compartments V^A and V^{R_k} (k =1, 2, 3) are equivalent to A and R_i. Individuals enter the compartment V^S with a vaccine efficacy reduced according to the amount of time they spent in V^A and V^{R_k}. The actual deployment of the vaccine doses in shown in figure S17.

Other interventions WaSH and other intervention efforts are not explicitly considered in the model, but their impact is implicitly taken into account by calibrating of the exposure rates β^i to disease incidence that occurred while interventions were taking place. β^i is modelled to be constant in time, meaning that changes in number or type interventions or population behaviour [33] over time are not taken into account.

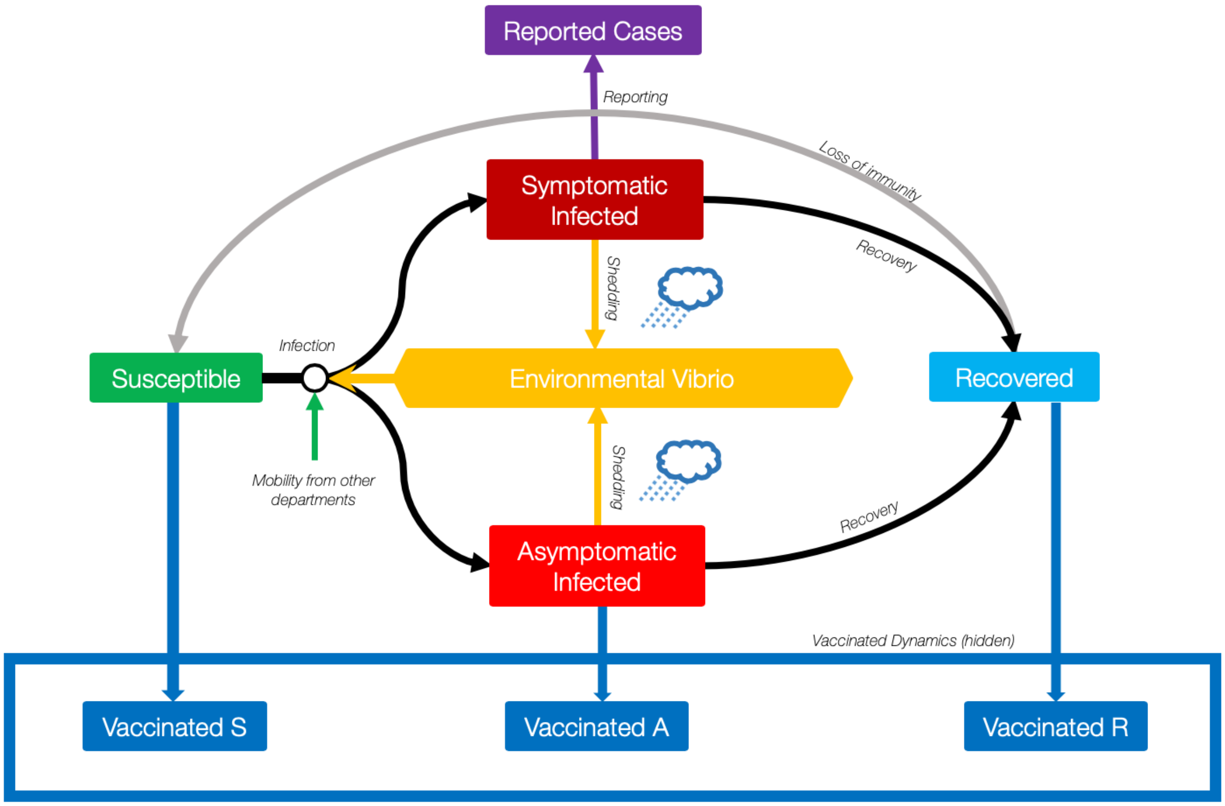

Figure S16: Schematic diagram of the cholera transmission in a single department. Dynamics of vaccinated compartments are not shown.

4.1.2 Model equations

The model is implemented as a stochastic counting process [42]. Let $N_{AB}(t)$ be the number of individuals transiting between compartments $A, B \in \mathcal{X}$ in the time interval $[0, t)$ where $\mathcal{X}$ is the state vector,

$$\mathcal{X} = \{S, I, A, R_k, V^S_{j,1d}, V^A_{j,1d}, V^{R_k}_{j,1d}, V^S_{j,2d}, V^A_{j,2d}, V^{R_k}_{j,2d}\} \text{ for } j = 1, ..., J, \text{ and } k = 1, 2, 3,$$

and J is the number of vaccination campaigns in the department. The number of transitions during a time-step Δt is $\Delta N_{AB}(t) = N_{AB}(t + \Delta t) - N_{AB}(t)$. Given the state of the system at time t, $\mathcal{X}_t$, and a force of infection $F_j(t)$ the transition rates read (transitions written only for 1 dose of OCV and one vaccination campaign):

$$\begin{aligned}
\mathbb{P}\left[\Delta N_{SI}(t) = 1 \middle| \mathcal{X}_t\right] &= \sigma F_j(t) S(t) \Delta t + o(\Delta t) \\
\mathbb{P}\left[\Delta N_{SA}(t) = 1 \middle| \mathcal{X}_t\right] &= (1-\sigma) F_j(t) S(t) \Delta t + o(\Delta t) \\
\mathbb{P}\left[\Delta N_{SV^S_{1d}}(t) = 1 \middle| \mathcal{X}_t\right] &= r_{V_{1d}}(t) S(t) \Delta t + o(\Delta t) \\
\mathbb{P}\left[\Delta N_{S\bullet}(t) = 1 \middle| \mathcal{X}_t\right] &= \mu S(t) \Delta t + o(\Delta t) \\
\mathbb{P}\left[\Delta N_{IR_1}(t) = 1 \middle| \mathcal{X}_t\right] &= \gamma I(t) \Delta t + o(\Delta t) \\
\mathbb{P}\left[\Delta N_{I\bullet}(t) = 1 \middle| \mathcal{X}_t\right] &= (\mu + \alpha) I(t) \Delta t + o(\Delta t) \\
\mathbb{P}\left[\Delta N_{AR_1}(t) = 1 \middle| \mathcal{X}_t\right] &= \gamma A(t) \Delta t + o(\Delta t) \\
\mathbb{P}\left[\Delta N_{AV^A_{1d}}(t) = 1 \middle| \mathcal{X}_t\right] &= r_{V_{1d}}(t) A(t) \Delta t + o(\Delta t) \\
\mathbb{P}\left[\Delta N_{A\bullet}(t) = 1 \middle| \mathcal{X}_t\right] &= \mu A(t) \Delta t + o(\Delta t) \\
\mathbb{P}\left[\Delta N_{R_k R_{k+1}}(t) = 1 \middle| \mathcal{X}_t\right] &= 3\rho R_k(t) \Delta t + o(\Delta t), \quad k = 1, 2 \\
\mathbb{P}\left[\Delta N_{R_3 S}(t) = 1 \middle| \mathcal{X}_t\right] &= 3\rho R_3(t) \Delta t + o(\Delta t) \\
\mathbb{P}\left[\Delta N_{R_k V^{R_k}_{1d}}(t) = 1 \middle| \mathcal{X}_t\right] &= r_{V_{1d}}(t) R_k(t) \Delta t + o(\Delta t) \quad k = 1, 2, 3 \\
\mathbb{P}\left[\Delta N_{R_k\bullet}(t) = 1 \middle| \mathcal{X}_t\right] &= \mu R_k(t) \Delta t + o(\Delta t) \quad k = 1, 2, 3 \\
\mathbb{P}\left[\Delta N_{V^S_{1d} I}(t) = 1 \middle| \mathcal{X}_t\right] &= \sigma(1 - \eta^{i,j}_{1d}(t)) F_j(t) V^S_{1d}(t) \Delta t + o(\Delta t) \\
\mathbb{P}\left[\Delta N_{V^S_{1d} A}(t) = 1 \middle| \mathcal{X}_t\right] &= (1-\sigma)(1 - \eta^{i,j}_{1d}(t)) F_j(t) V^S_{1d}(t) \Delta t + o(\Delta t) \\
\mathbb{P}\left[\Delta N_{V^S_{1d}\bullet}(t) = 1 \middle| \mathcal{X}_t\right] &= \mu V^S_{1d}(t) \Delta t + o(\Delta t) \\
\mathbb{P}\left[\Delta N_{V^A_{1d} V^{R_1}_{1d}}(t) = 1 \middle| \mathcal{X}_t\right] &= \gamma V^A_{1d}(t) \Delta t + o(\Delta t) \\
\mathbb{P}\left[\Delta N_{V^A_{1d}\bullet}(t) = 1 \middle| \mathcal{X}_t\right] &= \mu V^A_{1d}(t) \Delta t + o(\Delta t) \\
\mathbb{P}\left[\Delta N_{V^{R_k} V^{R_{k+1}}}(t) = 1 \middle| \mathcal{X}_t\right] &= 3\rho V^{R_k}(t) \Delta t + o(\Delta t), \quad k = 1, 2 \\
\mathbb{P}\left[\Delta N_{V^{R_3}_{1d} V^S_{1d}}(t) = 1 \middle| \mathcal{X}_t\right] &= 3\rho V^{R_3}_{1d}(t) \Delta t + o(\Delta t) \\
\mathbb{P}\left[\Delta N_{V^{R_k}\bullet}(t) = 1 \middle| \mathcal{X}_t\right] &= \mu V^{R_k}(t) \Delta t + o(\Delta t) \quad k = 1, 2, 3
\end{aligned} \tag{4.2}$$

assuming that $\mathbb{P}[\Delta N_{XY} > 1 | \mathcal{X}_t] = o(\Delta t)\ \forall X, Y \in \mathcal{X}$ and $\mathbb{P}[\Delta N_{X\bullet} > 1 | \mathcal{X}_t] = o(\Delta t)\ \forall X \in \mathcal{X}$. Note that $\mathbb{P}\left[\Delta N_{X\bullet}(t) = 1 \middle| \mathcal{X}_t\right]$ denotes probability that individuals die and it is governed by the same parameter μ for all compartments except I.

The ensuing stochastic variations of the state variables are:

$$
\begin{aligned}
\Delta I(t) &= \Delta N_{SI}(t) - \Delta N_{IR_1}(t) - \Delta N_{I\bullet}(t) \\
\Delta A(t) &= \Delta N_{SA}(t) - \Delta N_{AR_1}(t) - \Delta N_{AV^A}(t) - \Delta N_{A\bullet}(t) \\
\Delta R_1(t) &= \Delta N_{IR_1}(t) + \Delta N_{AR_1}(t) - \Delta N_{R_1R_2}(t) - \Delta N_{R_1V^{R_1}}(t) - \Delta N_{R_1\bullet}(t) \\
\Delta R_2(t) &= \Delta N_{R_1R_2}(t) - \Delta N_{R_2R_3}(t) - \Delta N_{R_2V^{R_2}}(t) - \Delta N_{R_2\bullet}(t) \\
\Delta R_3(t) &= \Delta N_{R_2R_3}(t) - \Delta N_{R_3S}(t) - \Delta N_{R_3V^{R_3}}(t) - \Delta N_{R_3\bullet}(t) \\
\Delta V^S(t) &= \Delta N_{SV^S}(t) - \Delta N_{V^SI}(t) - \Delta N_{V^SA}(t) - \Delta N_{V^S\bullet}(t) \\
\Delta V^A(t) &= \Delta N_{AV^A}(t) - \Delta N_{V^AV^{R_1}}(t) - \Delta N_{V^A\bullet}(t) \\
\Delta V^{R_1}(t) &= \Delta N_{R_1V^{R_1}}(t) + \Delta N_{V^AV^{R_1}}(t) - \Delta N_{V^{R_1}V^{R_2}}(t) - \Delta N_{V^{R_1}\bullet}(t) \\
\Delta V^{R_2}(t) &= \Delta N_{R_2V^{R_2}}(t) + \Delta N_{V^{R_1}V^{R_2}}(t) - \Delta N_{V^{R_2}V^{R_3}}(t) - \Delta N_{R_2\bullet}(t) \\
\Delta V^{R_3}(t) &= \Delta N_{R_3V^{R_3}}(t) + \Delta N_{V^{R_2}V^{R_3}}(t) - \Delta N_{V^{R_3}V^S}(t) - \Delta N_{V^{R_3}\bullet}(t) \\
S(t) &= H_i - \sum_{X \in \mathcal{X} \setminus \{S\}} X(t),
\end{aligned}
\tag{4.3}
$$

where the equation for $S(t)$ enforces a constant total population. The rescaled bacterial concentration B is necessary to estimate the force of infection and is computed using the following ODE:

$$
\frac{dB}{dt} = -\mu_B B + \left(1 + \lambda \left(J(t)\right)^r\right) D_i \left[\theta_I I + \theta_A A\right] \tag{4.4}
$$

with D_i the average population density of the department (total department population over department area) and $J(t)$ the precipitation over time. Parameter μ_B expresses the mortality rate of the bacteria in the environment, θ_I and θ_A are the shedding rates of symptomatically and asymptomatically infected individuals, and λ and r are the parameters of the power-law that controls the non-linear impact of precipitation.

Let $C(t_j)$ denote the number of people that develop symptoms and seek healthcare during the observation interval $[t_j, t_{j+1})$ (i.e. the true incidence). Thus:

$$
C(t_j) = [N_{SI}(t_{j+1}) - N_{SI}(t_j)] + [N_{V^SI}(t_{j+1}) - N_{V^SI}(t_j)]. \tag{4.5}
$$

A full partially observed Markov process formulation requires a measurement model linking the time series of the reported incidence to $C(t_j)$, in addition to the process model in (4.2). We use a negative-binomial measurement model accounting for over- or under-reporting of cholera incidence, i.e.

$$
\text{cases}(t_j) \sim \text{NB}(\epsilon(t) C(t_j), p).
$$

where $\epsilon(t) > 0$ represents the proportion of cases reported. To account for the change of the case definition that occurred on January 1st, 2018, the reporting rate changes over time:

$$
\epsilon(t) = \begin{cases} \epsilon_1 & \text{if t < Jan 1st, 2018} \\ \epsilon_2 & \text{otherwise} \end{cases} \tag{4.6}
$$

The parameters of the model are shown in Table S14.

4.1.3 Additional Data

- Satellite-based daily precipitation measurements from October 2010 to March 2015: TRMM 3B42 RT Derived Daily Product [44], https://disc2.gesdisc.eosdis.nasa.gov/dods/TRMM_3B42RT_Daily_7.info
- GPM satellite-based precipitation measurements from April 2015 to December 2016 https://disc.gsfc.nasa.gov/datacollection/GPM_3IMERGDL_04.html.
- Population of Haitian departements: from the Institut Haitien de Statistique et d'Informatique (IHSI): http://www.ihsi.ht/pdf/projection/Estimat_PopTotal_18ans_Menag2015.pdf

Table S14: Parameters of the model.

Parameter	Calibration	Value or bound	Unit	description
β^i ($\times$10 dept.)	yes	$[0, \infty]$	–	Exposure
c^i ($\times$10 dept.)	yes	$[0, \infty]$	–	Force of infection in dept. i from cases in other depts.
ϵ_1	yes	$[0, 2]$	–	reporting fraction before January 1st, 2018
ϵ_2	yes	$[0, 2]$	–	reporting fraction after January 1st, 2018
σ_w	yes	$[0, 0.1]$	–	std-dev of the perturbation of $F(t)$
p	yes	$[0, \infty]$	–	dispersion parameter of reporting
θ_I	yes	$[0, \infty]$	–	Shedding sympt.
θ_A	yes	$[0, \theta_I]$	–	Shedding asympt.
μ_B	yes	$[0, \infty]$	d^{-1}	Bacterial mortality in environment
r	yes	$[0, \infty]$	–	Exponent rainfall
λ	yes	$[0, \infty]$	–	Coef. rainfall
ρ	no	$1/(8 \cdot 365)$	d^{-1}	Loss of immunity [19, 20]
σ	no	0.25	–	Symptomatic/exposed
α	no	0.004	d^{-1}	Mortality due to cholera [43]
γ	no	$1/5$	d^{-1}	Recovery rate sympt. [39]
k	no	3	–	number of recovered compartments
μ	no	$1/(63.6 \cdot 365)$	d^{-1}	mortality rate from life expectancy (World bank, 2017)
$\eta_{1d}(t), \eta_{2d}(t)$	no	as in spec.	–	VE for 1 and 2 doses

- Departmental level cholera cases October 2010 - January 2019: extracted from the Ministère de la Santé Publique et de la Population (MSPP) reports, `https://mspp.gouv.ht/newsite/documentation.php`, obtained in the MSF dashboard `https://epicentre-msf.shinyapps.io/haiti-2016-cholera/`.

Rainfall measurements are provided on a regular grid. To get a value for each department we averaged the measurements over the extent of the department.

4.1.4 Model runs

We construct a future rainfall time series up to the year 2030 by sampling from the past 20 years of data with replacement blocks of 15 days. To keep the correct seasonality the day of the year of each block is preserved.

The model was simulated with a constant time-step of 4.8h, and the ODE for the bacterial concentration was integrated using a Runge-Kutta 4 scheme.

The deployment of vaccination for each scenario is shown in figure S17.

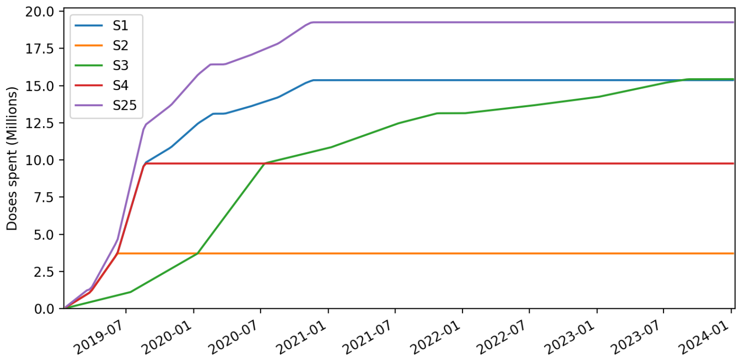

Figure S17: Deployment over time of the vaccination doses in the four scenario with vaccination.

4.2 Model Selection and Fitting/Calibration

The model is calibrated separately for each department on the weekly reported cases from 2014-03-01 to 2019-01-12. The calibration procedure is based on a frequentist multiple iterated filtering algorithm (MIF2 [45]). The initial conditions on March 1st, 2014 are derived by enforcing the model dynamics on the reported cases from the start of the epidemic in 2010. The MIF2 algorithm performance deteriorates quickly with the spatial dimension of the model as the number of particles needed for calibration increase exponentially [46]. To address this problem we first calibrate each department independently. In a second step, using the departmental calibration as a starting point, we calibrate the entire spatial model. The department-specific calibration procedure is as follow:

1. All unknown parameters (see Table S14) are calibrated on the reported cases of Artibonite, where the epidemic had a clear seasonal dynamic from 2014 to 2018 with a sufficiently large number of cases, thus providing a good signal for the model. This allows to calibrate the unknown epidemiological and rainfall-related parameters on the most informative time series available.
2. For the other nine departments, we calibrate the most sensitive parameters, the exposure β^i and the mobility parameter c^i only, while fixing the remaining parameters to their best fit found for Artibonite.
3. We exclude the large, rainfall unrelated cholera outbreak in the Ouest department in 2015-2016 (mainly Port-au-Prince) [47] from the calibration since we consider that it's not part of the endemic dynamics we are focusing on in this study.

During this phase, the mobility coefficients c_i are calibrated using the reported cholera cases (data) from the other departments (appropriatly scaled with the reporting rate and symptomatic fraction).

After visual convergence is reached in each departements, we now use the departmental best fits as starting points for a country-wide calibration. This mainly affects the mobility parameter c_i, which governs the departmental inter-dependence, as it now calibrated on the actual simulated incidence from other departments.

The final parameters are shown in Table S15.

4.3 Assessment of Model Fit

The calibrated parameters are shown in table S15. The model fit is shown in Figure S18. An example of the number of individuals in the different compartments over time is shown in Figure S19.

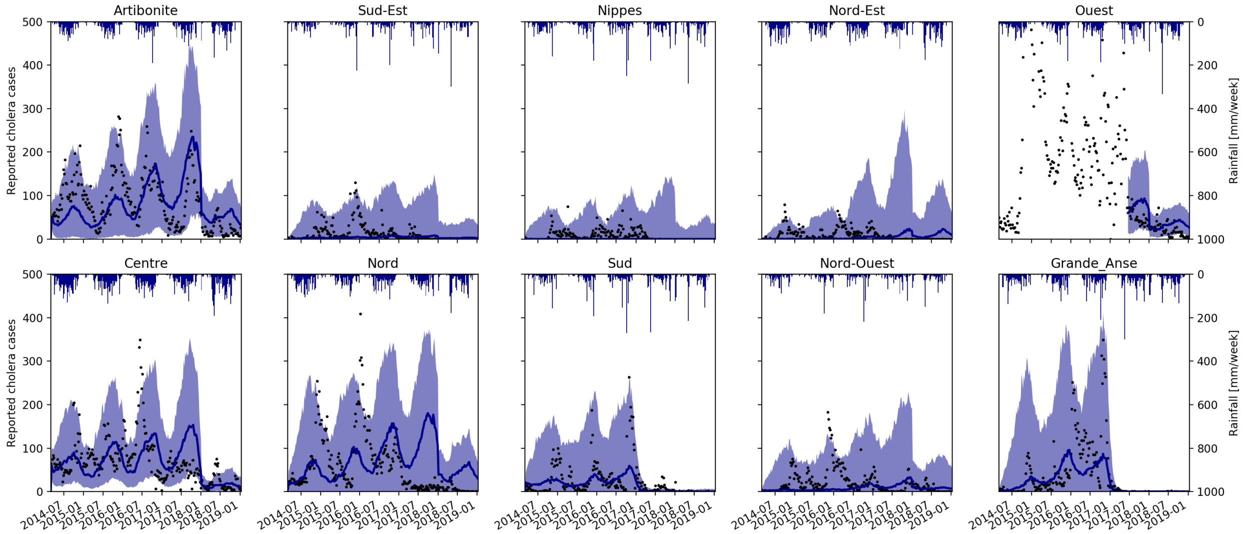

Figure S18: Fit on calibration data for the best parameter of the model. The median (blue line) and the q025 and q975 quantiles (shaded area) over 1000 realization of the stochastic model are shown. Weekly reported cholera cases are shown as black dots.

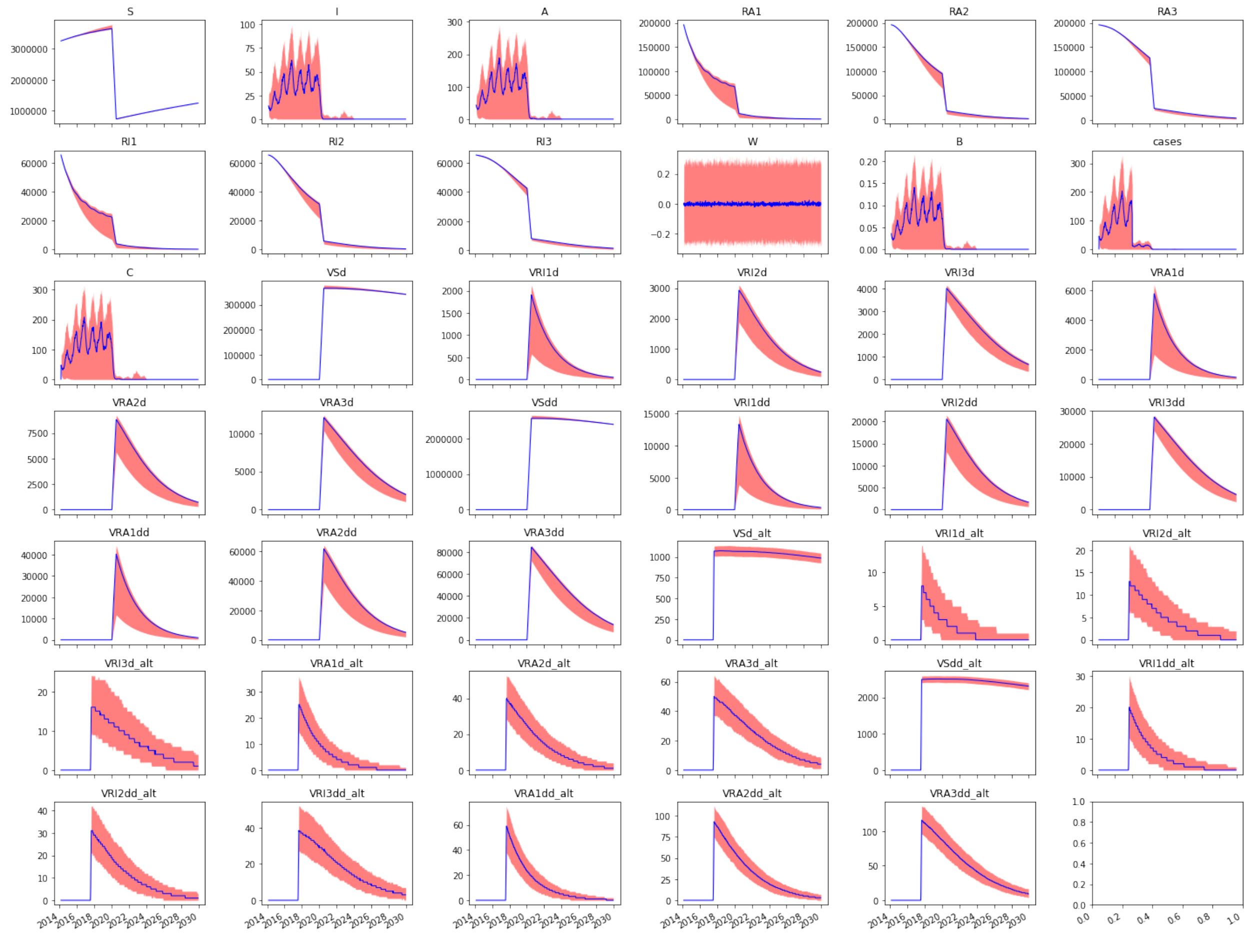

Figure S19: Total number of individual belonging to each compartment in the Ouest department, for vaccination scenario S3. Median values over 500 runs are shown in blue. The uncertainty bands represent the 2.5th and the 9.75th quantiles. Compartments with the suffix ”_alt” indicate already conducted vaccination campaigns that took place in Haiti (see the meta-supplement). Other vaccination compartments concern the potential future mass vaccination campaigns that are the subject of this study.

Table S15: Value of the maximum likelihood parameters after calibration.

Parameter	Departement	Value	Unit
β^i			–
	Artibonite	0.51	–
	Sud-Est	1.38	–
	Nippes	2.99	–
	Nord-Est	3.24	–
	Ouest	0.09	–
	Centre	1.9	–
	Nord	0.58	–
	Sud	1.30	–
	Nord-Ouest	1.14	–
	Grand'Anse	2.82	–
c^i			
	Artibonite	$1.53e^{-6}$	–
	Sud-Est	$6.10e^{-7}$	–
	Nippes	$3.05e^{-7}$	–
	Nord-Est	$8.21e^{-7}$	–
	Ouest	$1.07e^{-6}$	–
	Centre	$1.06e^{-5}$	–
	Nord	$5.31e^{-7}$	–
	Sud	$1.03e^{-6}$	–
	Nord-Ouest	$5.85e^{-7}$	–
	Grand'Anse	$8.76e^{-7}$	–
ϵ_1		0.97	–
ϵ_2		0.10	–
σ_w		0.0081	–
p		101.2	–
θ_I		$3.45e^{-4}$	–
θ_A		$1.5e^{-5}$	–
μ_B		0.36	d^{-1}
r		0.31	–
λ		0.27	–

Model-4 Supplement

Dennis L. Chao
Institute for Disease Modeling

5.1 Model Description

The Institute for Disease Modeling cholera transmission model is based on a previous model described in [2]. The model has been extended to allow it to be run over multiple years and to use rainfall data to drive cholera transmission. Many of the model parameters are summarized in Table S16, including those associated with the additions to the model. The model source code and data are available at: https://github.com/dlchao/cholera-model-haiti-2018.

The model is individual-based, so that each individual is explicitly represented in a synthetic population of Haiti. The synthetic population is spatially distributed on a 1km $\times$ 1km grid to represent the spatial extent of Haiti (Figure S20). For each grid cell, households are sampled from the appropriate Haitian department from the microcensus data from the Haitian Institute of Statistics and Informatics distributed by IPUMSi [48], based on 2003 census data. Each cell is populated with enough households to approximate the gridded 2015 population estimate from WorldPop [49]. Specifically, we aggregate the 100m gridded estimate HTI_ppp_v2b_2015.tif (2015 estimate, not UN-adjusted) to a 1km $\times$ 1km grid to compute the target population of each cell. The ages of the individuals in the synthetic households is taken from the households sampled from the microcensus data, which reports ages to the nearest year. The age structure of the synthetic population should thus reflect the age structure of the Haitian population at the national, departmental, and household levels. The synthetic population has 10,911,656 people, 11.7% are under 5 years old and 212,230 (1.94%) of whom are 0 years old.

Individuals in each cell are aggregated into well-mixed communities of about 1,000 individuals, within which transmission occurs. When there are fewer than 1,500 individuals in a cell, they are considered to be a single community. Individuals can be susceptible, exposed, infectious, or recovered from cholera (Figure S21). Susceptible individuals can be infected either by exposure to infectious household members by exposure to contamination in the environment. Once infected, the individual is asymptomatic and non-infectious for a 1 to 5-day latent period. After the latent period, the individual has an 80% chance of becoming asymptomatically infected and a 20% chance of becoming symptomatically infected. Infectious individuals can both infect susceptible household members directly or shed *Vibrio* into the local environment associated with each community. We assume symptomatic infections shed 10$\times$ more than asymptomatic. Although symptomatic infections have been associated with orders of magnitude more shedding, we hypothesized that it would not be associated with proportionately higher transmission if symptomatic cholera incapacitates people (hampers mobility and social contacts). *Vibrio* in the environment decays at an exponential rate with a 14-day halflife. We had tested the model with shorter halflifes and found that the cholera frequently disappeared well before 2019, while values of 2 weeks or more allowed for persistence. If a community is on a river, infectious individuals shed into the river as well. Environmental *Vibrio* shed within the past day is *hyperinfectious* [50]. The daily probability of infection from environmental *Vibrio* in the local community is:

$$\text{Probability of infection} = \beta \frac{(B + 2B_H)/N + B_R + 2B_{RH}}{\kappa + (B + 2B_H)/N + B_R + 2B_{RH}} \tag{5.1}$$

where B is the amount of contamination in the local environment, B_R is the amount of contamination in the local river (if the community is on a river), B_H is the amount of hyperinfectious *Vibrio* (shed within the last day) in the environment, B_{RH} is the amount of hyperinfectious *Vibrio* in the river, κ is the environmental half-saturation constant, and β is a scalar that can be adjusted to change the infectiousness of environmental *Vibrio*. Note that hyperinfectious *Vibrio* is assumed to be twice as infectious as non-hyperinfectious. After a 1 to 3-day infectious period, individuals recover and are immune to infection. In the current version of the model, immunity can wane. Each day, there is

a small chance that recovered individuals become susceptible (simulates exponential distribution of protection from natural infection) so that the average duration of immunity is 8 years. This long duration of protection was necessary to replicate the low levels of cholera for years after the initial outbreak, which we believe conferred protection to much of the population through infection. We found that when the immunity was shorter (3 or 5 years), the cholera epidemic resurged well before 2019. We added temporary immunity to the model for newborns. We draw a uniform random number from 0 to 364 days for each newborn to determine the length of full immunity. This is for two reasons: infants are at low risk of exposure (breastfeeding) and the synchronous fully susceptible birth cohort could trigger epidemics.

The model truncates very low probabilities of infection from the environmental reservoir to 0. Because the *Vibrio* level in the environmental reservoir decays exponentially, it never reaches 0 and the daily probability of infection from the reservoir should likewise approach but never reach 0. But because of the limitations of the random number generator (RNG) used, the daily probability of infection from the reservoir never dropped below 2^{-32}, which caused spontaneous resurgences of cholera long after apparent elimination occurred. In the model, a random event (such as infection) occurs if the value drawn from the random number generator is less than the probability of the event in question. The model uses GSL's 32-bit random number generator (GNU Scientific Library version 2.5, https://www.gnu.org/software/gsl/doc/html/index.html), which generates pseudorandom numbers in the range of 0 to 4294967295 then converts these integers to doubles by dividing by 4294967296. Since the RNG returns 0 with a probability of $1/2^{32}$, then the lowest probability our code can handle is 2^{-32}. We addressed this issue by assuming no infection occurs if the daily infection probability from the reservoir is below 2^{-32}.

Transmission of disease across communities can occur in several ways: commuting to work, traveling along highways, sporadic long-distance travel, and rivers, which are described in more detail in [2]. Working-age individuals spend a portion of their time in communities some distance away according to a power law. They can therefore become exposed to cholera at these work destinations or shed cholera in them. Because the model now includes age, we now specify that 60% of individuals 15 years old and older "commute" to work, based on Haiti's employment-to-population ratio reported at https://fred.stlouisfed.org/series/SLEMPTOTLSPZSHTI. Major highways are included in the model. Individuals living next to a highway have a small chance each day of swapping positions with another individual along the road network. This simulations the travel of susceptible of infectious individuals on longer trips while keeping the population size of their respective communities constant. Even less frequently, random individuals from across the country can be swapped, which mimics sporadic long-range travel that is not dictated by the road net-

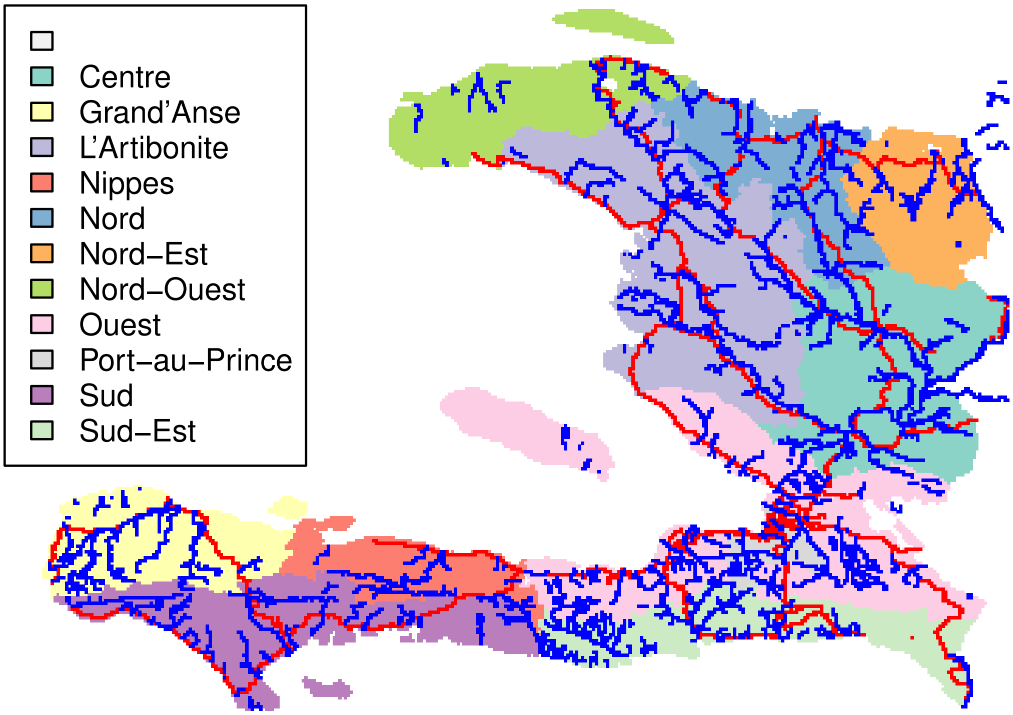

Figure S20: Map of departments and major features of Haiti. Rivers are in dark blue, highways in dark red, and the population associated with each department is in the color indicated by the legend.

work. The locations and directionality of rivers are included in the model. Vibrio shed into rivers travels downstream each day, potentially infecting individuals in downstream communities. Highway and river locations were obtained from OpenStreetMap [51].

The new version of the model ages the population for simulating multi-year epidemics. We age the population on March 15 of every year, which has been reported to be the "low season" for cholera [52]. Within the model individuals are not explicitly aged, but instead we copy the immune status (but not active infections) from individuals one year younger in the same "community" (about 1000 individuals or less), as we did in a previously described dengue model [53]. Newborns (age 0 years) are set to be unexposed. Household structures and ages are kept constant. We found that if we drew from the same 1kmx1km grid instead of community, Port-au-Prince and Nord would experience out-of-season outbreaks in April. These regions contain the most densely populated part of Haiti, and might sustain cholera through the dry season.

We assume that environmental shedding by infectious people is dependent on a department's rainfall that day. We obtain rainfall by computing the average rainfall that falls across the extent of each department each day according to the STAR Satellite Rainfall Estimates from 2010 through 2018 (https://www.star.nesdis.noaa.gov/smcd/emb/ff/HEsouthAmerica.php). When simulating years after 2018, we draw from rainfall data in one-year blocks (January 1 through December 31) from 2010 through 2018 excluding 2013, which had a long gap in the data. We used the rainfall estimates for Ouest department for the population of Port-au-Prince. If rainfall data are missing, we assume there is no rain that day. For modeling simplicity, we assume all years are 365 days, so we skip leap days in the weather data. Daily rainfall ranged form 0 to over 330mm of rain, and there is generally more rain in the south than in the north (Figure S22). The amount of *Vibrio cholerae* shed into the environment is multiplied by one of 5 scalars based on the day's rainfall. Environmental shedding is lowest on days with no rainfall (about 55% of days depending on the department), and higher when there is more rainfall, as defined by four categories: $<$ 1mm (about 16% of all days), $<$ 10mm, $<$ 50mm, or $\geq$ 50mm rainfall in a day (Figure S22A).

As in the previously published version of the model, vaccine can protect against infection (VE_S), illness given infection (VE_P), and infectiousness (VE_I). We generally assign either VE_S or VE_P to be > 0 and the other parameters to 0. In the previous version of the model we had protection from vaccine increase from 0 to full efficacy over a period of two weeks in order to simulate a dosing schedule. We now assume that vaccine is effective upon administration, since timing will be less critical when vaccine is administered years after the epidemic peak in 2010–2011. We also only model vaccination with both doses of cholera vaccine, so we do not model those who only get one dose. We assume that vaccine is only 46.9% as protective among children under 5 years old than those older [6]. We believe that this reduced protection depends on the age at vaccination, not age at exposure [54]. We assume that vaccine protection

Figure S21: Model structure. A) Model compartmental diagram, modified from reference [2]. The new features include waning of immunity (red arrow) and the seasonally-driven environmental shedding (blue arrows). For this project, we explore the effect of rainfall on shedding (blue arrows), the impact of vaccination (green dashed arrows), and infectiousness of V cholerae in the environment (black arrow from "Environment" to the Susceptible–Exposed transition arrow). B) Model overview diagram.

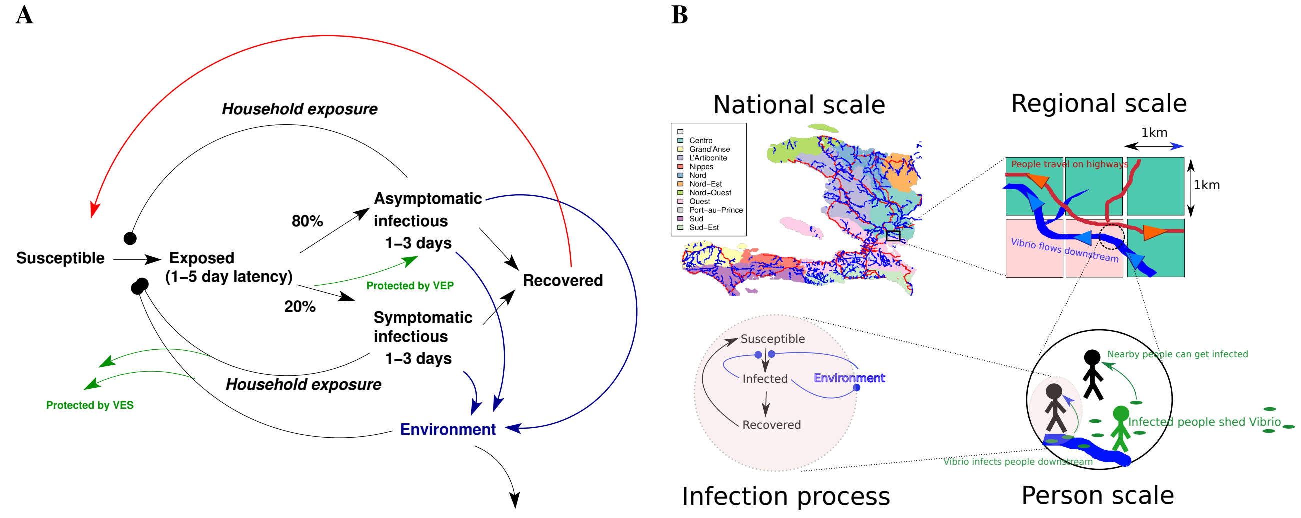

either does not wane or wanes linearly over time to approximate the log-linear waning scenarios (Figure S23).

Figure S22: Rainfall data. A) Amount of rainfall per day (averaged over departments). Each point on the y-axis is actually a "department–day", so the total number of data points is the number of days from 2010 to 2017 times 10. The highest amount of rainfall a department had in one day was 326.5mm. The bar on the right ("<330mm) includes all department–days of data. B) Weekly suspected cholera cases and monthly rainfall. Monthly rainfall by department is plotted below the x-axis. The gray bar indicates a 127-day gap in the satellite data from 2013.

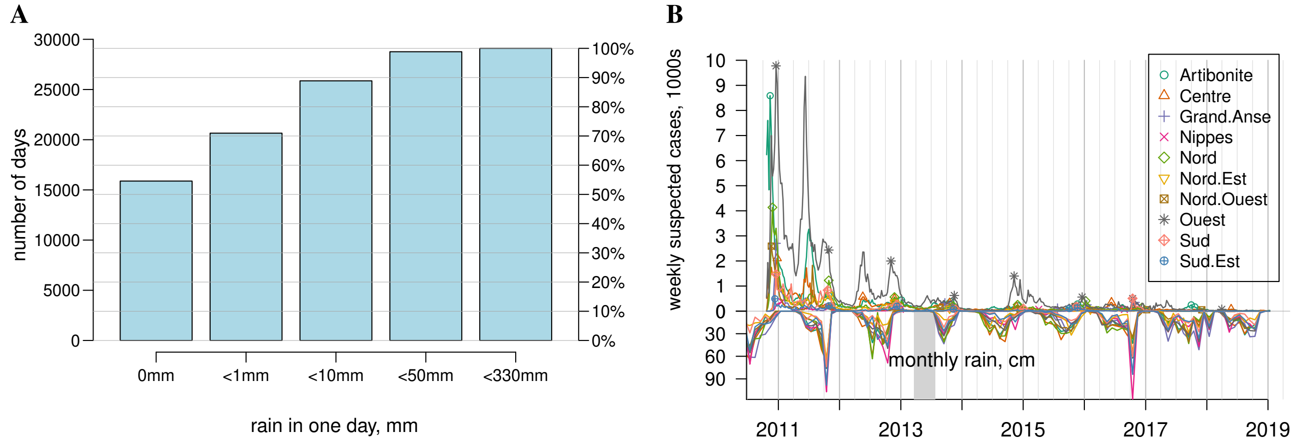

Table S16: Subset of model parameters. Some parameter values were taken from the previously published version of the model and further explanation can be found in [2].

parameter	default (range tested)	source/notes
incubation period	1–5 days	[55, 2]
infectious period	1–3 days	[18, 56]
duration of immunity after infection	8 years	
fraction symptomatic	20%	[2]
asymptomatic/symptomatic shedding ratio	10%	[2]
maximum daily probability of infection (β)	0.1 (0.05–0.35)	parameter sweeps A and B
environmental half saturation (κ)	0.2 (0.1–1.2)	parameter sweep A
shedding with low rain	1.0	reference
shedding with no rain	0.3 (0.2–0.8)	parameter sweeps B and C
shedding with heavy rain	6 (2–10)	parameter sweep C
decay of *V cholerae* in the environment	1/14 days^{-1}	
hyperinfectious *V cholerae* infectiousness	2$\times$	
amount shed into river (relative to environment)	1%	

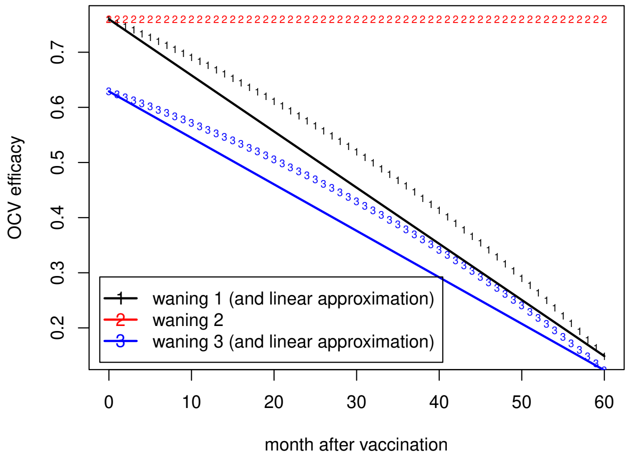

Figure S23: Vaccine efficacy waning scenarios. The consortium proposed 1-VE to decay at a log-linear rate, with a linear rescaling of efficacy for VE waning scenario 1 (plotted as numbers). The model approximates this as linear decay (solid lines). VE continues to decline until efficacy is 0.

5.2 Model Selection and Fitting/Calibration

We qualitatively fit the cholera transmission model so that the weekly symptomatic cholera cases had a few of the key features as the nationwide weekly suspected cases reported by MSPP from 2010 through 2018 (Figure S24) [4]. Because the reporting rate of cholera likely differed across departments [57] and over time, we felt that matching the timing and relative magnitude of peaks would be more robust to these variations than attempting to quantitatively fit the data as a time series assuming a fixed reporting rate. However, to make it easier to visually compare the output of the model to MSPP reported cases, we computed a "reporting rate" that is simply the number of cases reported by MSPP from 2010 through 2018 divided by the number of cases generated by our model over the same time period (Figure S24), though this constant "reporting rate" was not part of the model fitting process. We focused on producing the two large peaks in the first year of the epidemic (from late 2010 through the fall of 2011), the regular peaks coinciding with the rainfall around October–November each year, and the persistence of cholera through at least January 2019. We were unable to consistently capture the peaks observed in 2012, possibly indicating an overestimate of the spread of cholera in 2011 that consumed too many susceptibles to allow for large outbreaks in 2012. Because of the computational cost of running the model, we performed a limited exploration of parameter space and used parameter value estimates from the earlier publication [2] when possible.

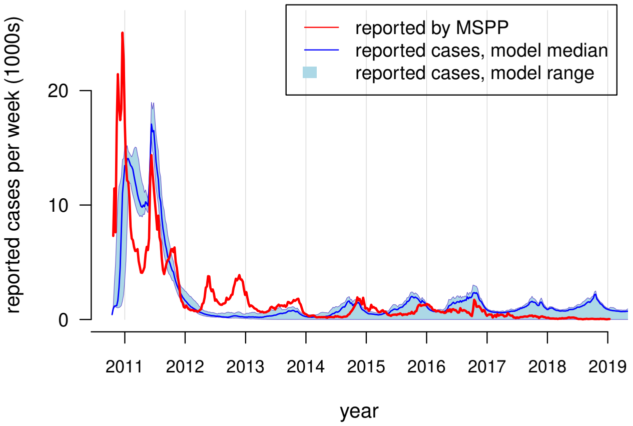

Figure S24: Model fit to MSPP data. The red line indicates the number of suspected cholera cases reported by MSPP per week. The blue line indicates the median number of symptomatic cholera cases per week multiplied by 0.3 (reporting rate), while the light blue region spans the minimum and maximum number from 30 stochastic model runs.

We performed three "sweeps" of parameters, varying only two free parameters per sweep. For the first parameter sweep ("A", see Figure S25), we varied κ and β (see Equation 5.1). These are the two parameters that govern overall transmissibility of cholera. In parameter sweep B, we fixed κ and varied β and the relative amount of shedding on days with no rainfall (Figure S25). We believe this parameter combination would determine the persistence of cholera during the dry seasons in Haiti. In parameter sweep C, we fixed κ and β and varied the relative amount of shedding on days with no rainfall and days with the highest levels of rainfall (Figure S25). We believe this parameter combination determines the duration and height of seasonal cholera peaks.

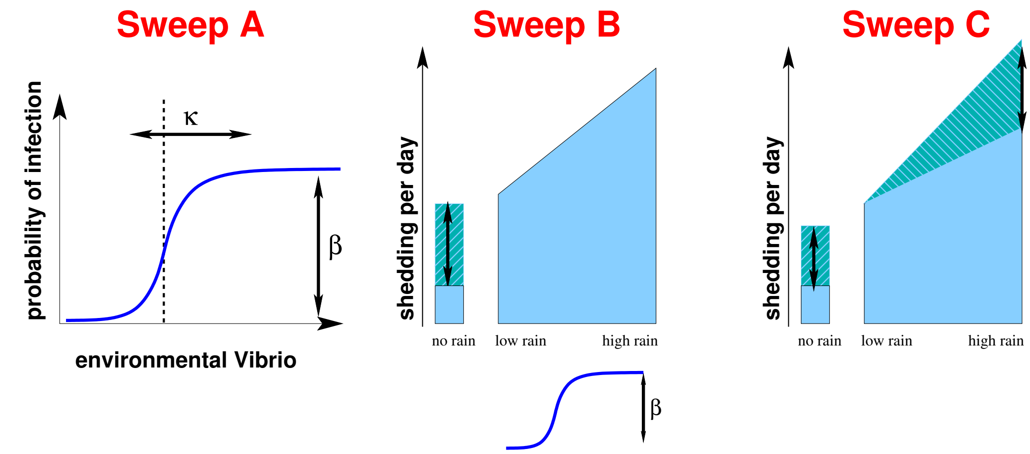

Figure S25: Diagram of the three parameter sweeps. In "sweep A", we vary κ and β. In "sweep B", we vary the amount of shedding when there is no rain and the amount of shedding when there is heavy rain. The amount shed when there is low rain is fixed and the intermediate levels are linearly interpolated. In "sweep C", we vary the amount of shedding when there is no rain and β.

5.3 Assessment of Model Fit

Since the earlier published version of the model, we have made several changes that affected our choice of parameter values. In [2], those infected with cholera were infectious from one to two weeks. Although infected people may in reality shed for over a week, we believe that a shorter period is more appropriate for the purposes of modeling transmission. [56] used historical data to estimate the serial interval to be 3.7 days, and this seems consistent with observations in Bangladesh [18]. Since we assume the latent period to be about 1.5 days (range of 1 to 5 days), then the infectious period should be 2 days in our model to match an approximately 3.5 day serial interval. We decided to make individuals infectious for 1 to 3 days (uniform distribution). Reducing the infectious period necessitated a cascade of other parameter changes. We also reduced the relative infectiousness of hyperinfectious *Vibrio*. Originally, we assumed that environmental *Vibrio* was 100× more infectious on the first day relative to subsequent days [50]. This effectively creates a "person-to-person" cholera transmission that is 100× stronger than the "environmental" transmission, which would basically be an SIRS model with a short serial interval and negligible environmental component. To allow the environmental component to play a more important role in transmission, we decreased the infectiousness of hyperinfectious *Vibrio* to be only 2× as infectious. After making all of these changes, we ran several 2-parameter sweeps to find appropriate values for the parameters introduced in this version of the model.

For parameter sweep A, we found that setting κ to 0.1-0.2 and β to 0.1 generated epidemic curves with two major peaks during the first year of the epidemic. Higher values of κ were often associated with years in which there were no seasonal peaks, and higher values of β caused cholera to spread throughout the country to rapidly so that there was only a single massive peak during the first year (Figure S26). When $\kappa = 0.2$ and $\beta = 0.1$, there were about 2,000,000 cases of cholera by the end of 2011 (Figure S27). In the MSPP reports [4], there were about 500,000 suspected cases by this point, implying a reporting rate of about 25%. Our model considers only "asymptomatic" and "symptomatic" cases and ignores the spectrum of illness that actually occurs, so it is difficult to interpret this estimated reporting rate. The MSPP also reports the estimated number of suspected cases who were under 5 years old and the number $\geq$5 years. In the model, about 10% of cases are under 5 when the epidemic starts, but this fraction climbs to about 20% within four years or less S28. This is because initially the entire population is fully susceptible to infection so cholera strikes all age groups equally, but as the epidemic progresses the population exposed to cholera ages and susceptible birth cohorts are introduced each year.

In parameter sweep B, we fixed κ and varied β and the relative amount of shedding on days with no rainfall (Figure S25). We found that when there was a high amount of shedding when there was no rainfall (e.g., shedding was reduced by a factor of 0.5 compared to days with minimal rainfall), the seasonal peaks of cholera started to flatten, but when shedding was too low during periods with no rain, cholera would often disappear after the second or third year of the epidemic (Figure S29). We found that shedding 0.3 on rainless days compared to days with minimal rain produced plausible seasonal peaks.

In parameter sweep C, we fixed κ and β and varied the relative amount of shedding on days with no rainfall and days with the highest levels of rainfall (Figure S25). We assume a linear relationship between rainfall and shedding to the environment, so shedding at intermediate levels of rainfall is interpolated between 1.0 (the base level of shedding when there is minimal but >0 rainfall) and the level of shedding at the highest levels of rainfall. When the relative shedding at the highest levels of rainfall was too low (e.g., only twice as high as at minimal rainfall), the seasonal peaks were too long. We suspect that there is more cholera transmission during the heavier rains from September to November than during the lighter rains from March through May. The seasonal peaks looked more realistic at higher levels of shedding with high rains (six or ten times as much shedding), but it was difficult to pick an optimal value (Figure S26).

To project the effectiveness of cholera vaccination, we simulated cholera transmission through 2029. For simulating transmission from 2010 through 2018, we used rainfall data from the appropriate years, but we re-used these data to simulate rainfall from 2019 onward. To do this, we drew random permutations of the years with sufficient rainfall data (2010 through 2018 except for 2013), then generated rainfall time series by concatenating data from these years (daily data from January 1 through December 31). We generated 30 rainfall time series, so that we could run the model 30 times. Thus, the model runs include both stochastic effects for all years and rainfall differences from 2019 onward. We also filled in the gap in data from April 1 2013 to July 25 2013 with data from randomly selected years. Because each run used different rainfall data after 2018, the model results had more variation after 2018 (Figure S31). Prediction of major events like hurricanes would improve forecasting of cholera transmission and the impact of vaccination [58].

We found it difficult to maintain cholera transmission at low levels, and we deliberately chose parameters to ensure

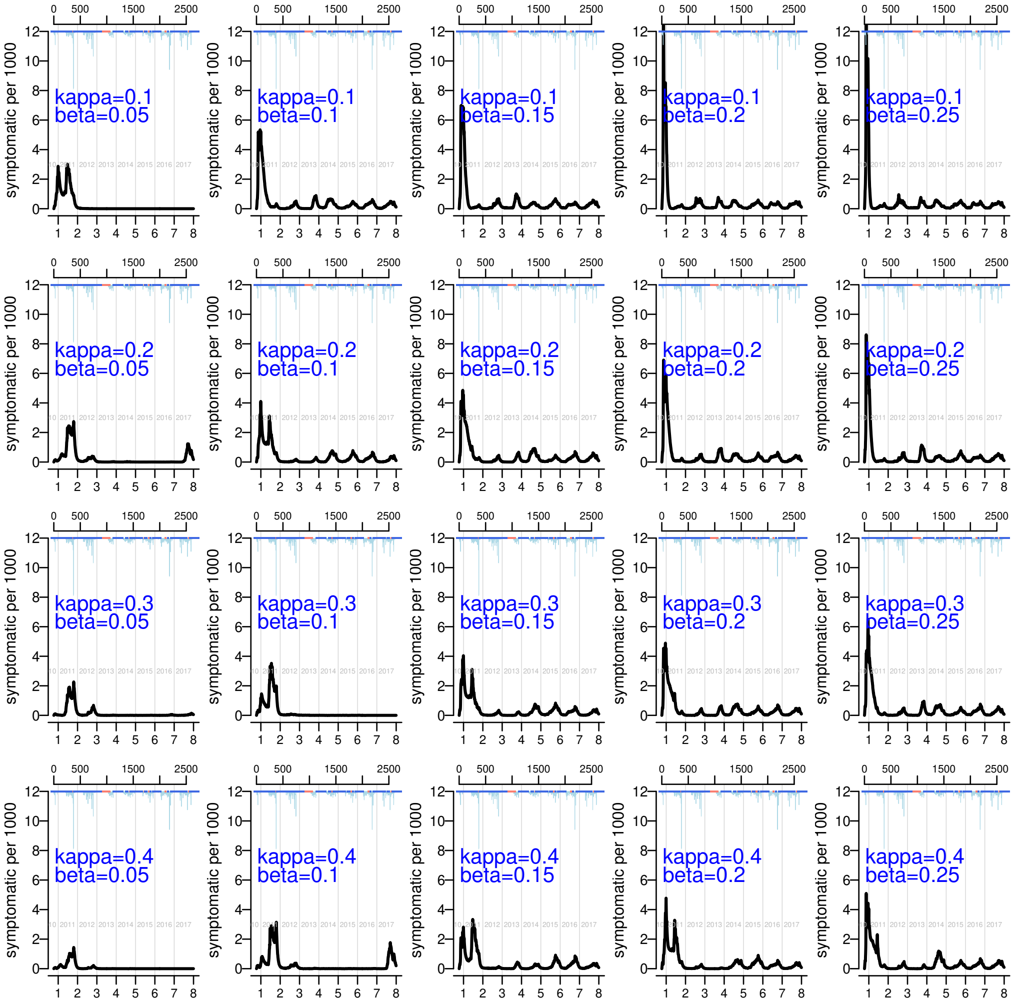

Figure S26: National symptomatic cholera prevalence in parameter sweep "A". The bottom x-axis has tick marks on January 1 of each year, and the top x-axis has the number of days of simulation. Year "1", the first full year of the simulations, corresponds to 2011. κ and β were varied. Shedding when there is no rain was 0.3 times shedding when there is minimal rain, and shedding is 6 times higher when there is heavy rain.

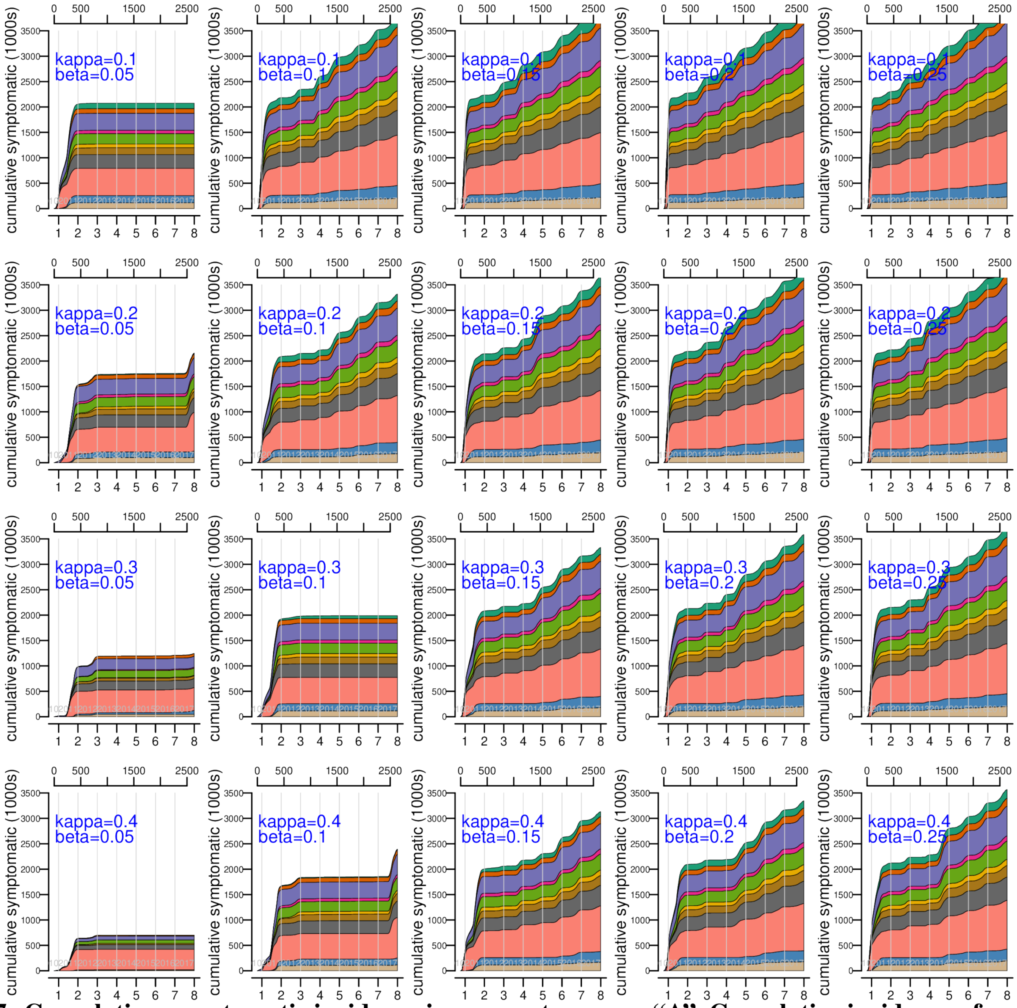

Figure S27: Cumulative symptomatic incidence in parameter sweep "A". Cumulative incidence of symptomatic cholera for each department is plotted as different colors.

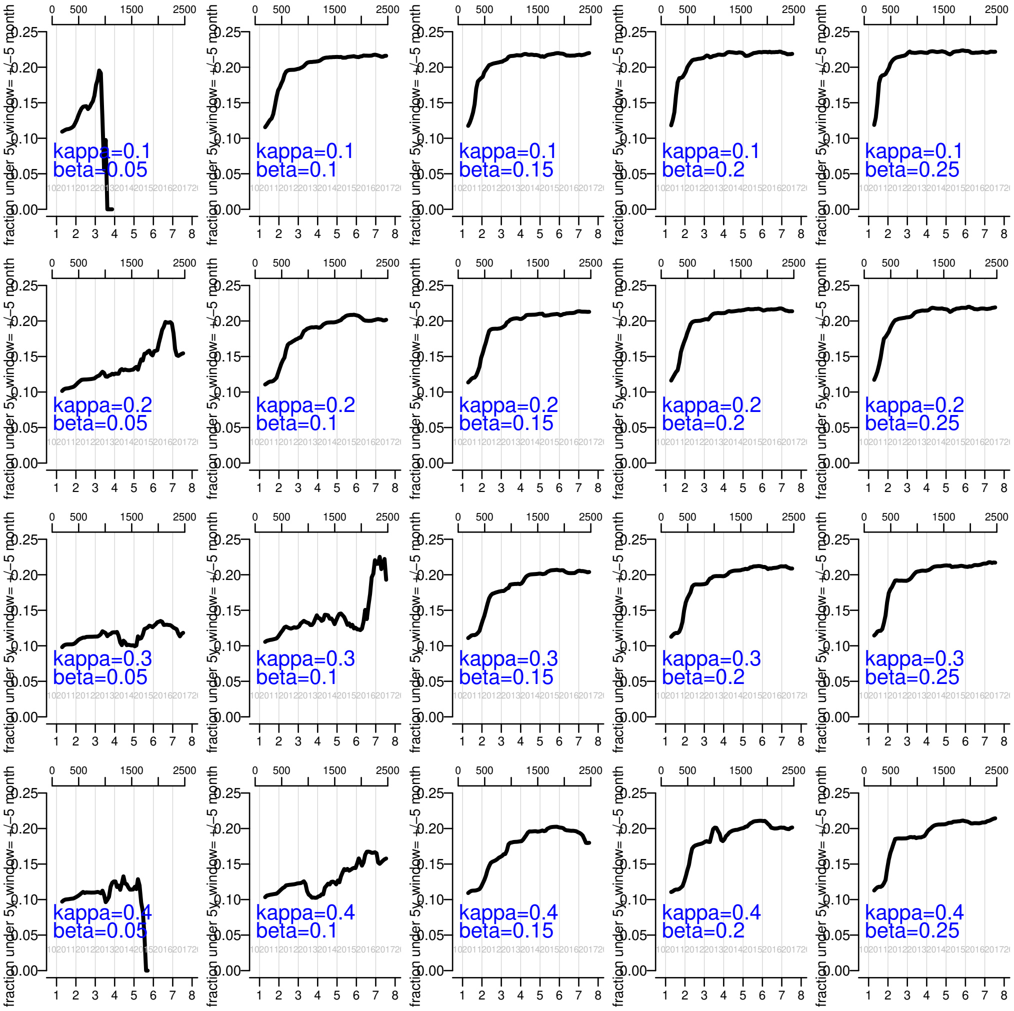

Figure S28: Proportion of symptomatic cases under 5 years old in parameter sweep "A". The proportion was computed over an 11-month moving window (using data from 5 months before to 5 months after to plot each month's proportion

.

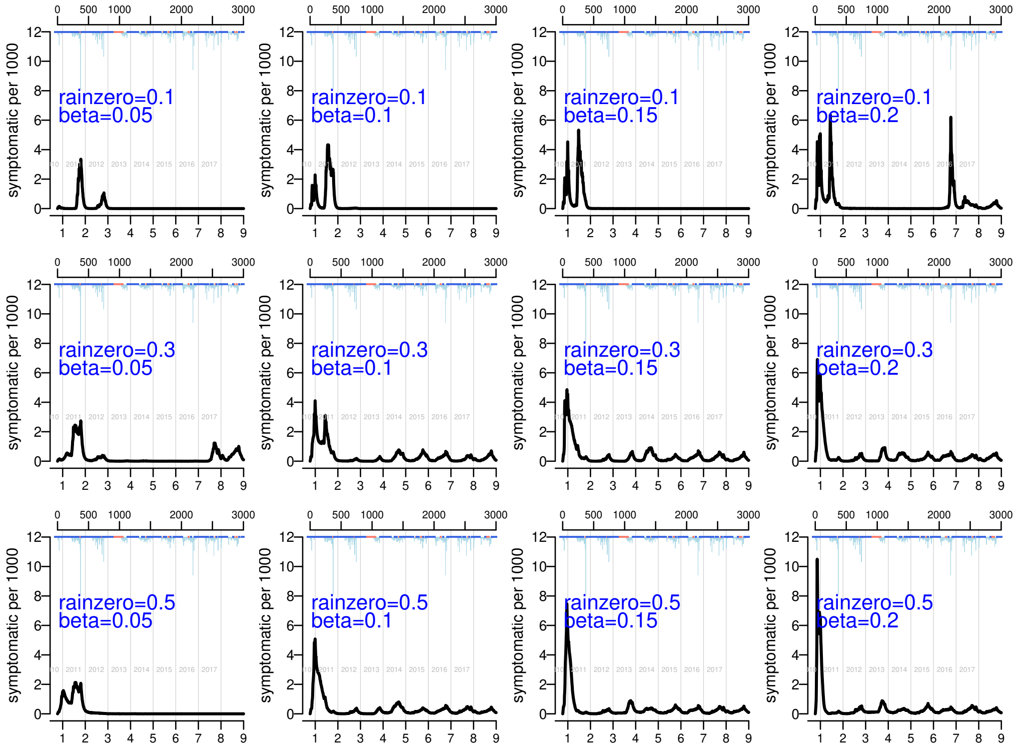

Figure S29: Symptomatic prevalence in a parameter sweep of beta and rainzero (sweep "B"). Cumulative incidence of symptomatic cholera for each department is plotted. The shedding when there is minimal rain is 1.0 and heavy rain is 6.0. Shedding when there is no rain (from top to bottom row: 0.1,0.3,0.5), and beta is varied from 0.1 to 0.25 (left to right: 0.1,0.15,0.2,0.25,0.3,0.35).

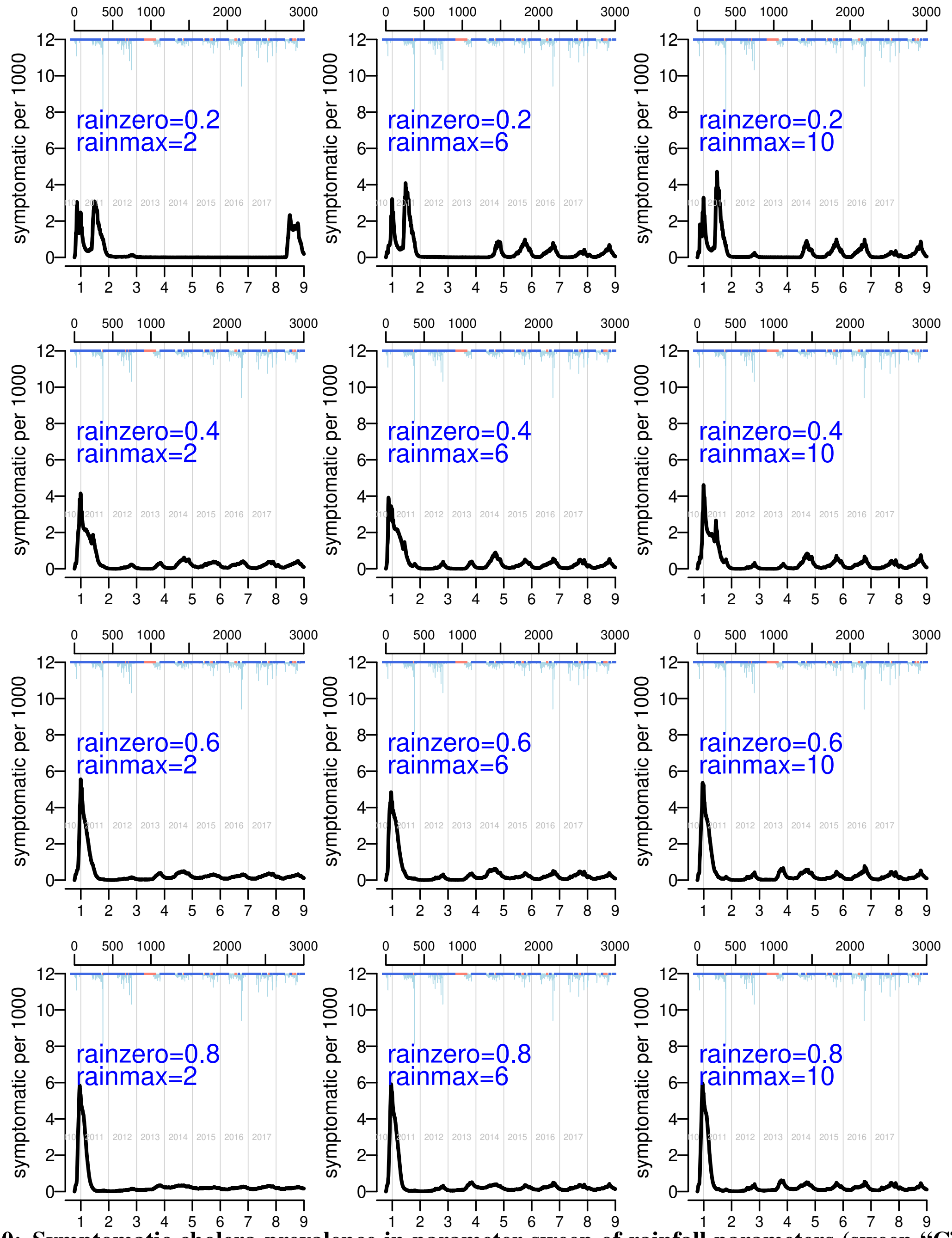

Figure S30: Symptomatic cholera prevalence in parameter sweep of rainfall parameters (sweep "C"). Prevalence of symptomatic cholera for each department is plotted. The bottom x-axis has tick marks on January 1 of each year, and the top x-axis has the number of days of simulation. The shedding when there is minimal rain is 1.0. Shedding when there is no rain (from top to bottom row: 0.2,0.4,0.6,0.8) and when there is heavy rain (from left to right column: 2, 6, 10) are simulated.

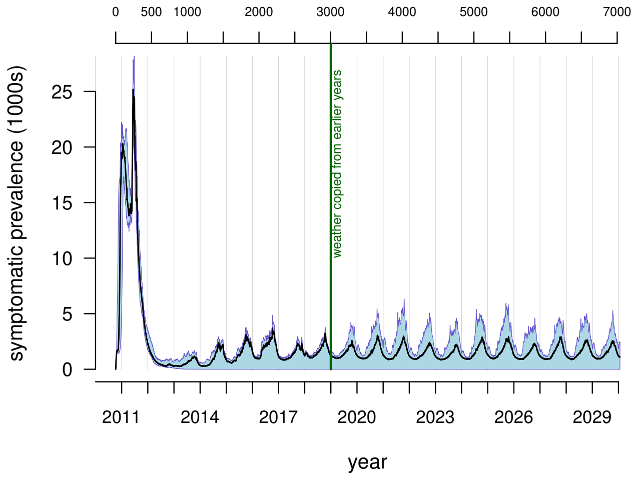

Figure S31: The results of 30 runs without interventions. Starting in January 2019, rainfall data is drawn from previous years. The blue region shows the minimum and maximum prevalence of symptomatic cholera across stochastic runs, and the black line shows the median. Rain data from 2010 to 2018 is used, and rain for each year after 2018 is copied from randomly selected years (excluding 2013).

persistence of cholera through 2019. We therefore consider our model fit to produce an overestimate of transmission, particularly after the initial epidemic peaks. Our model assumes that transmissibility of cholera, behavior of people, and the population size does not change over time. Given these constraints, the incidence of cholera slowly increases over time after the first waves in the model, since the population-level immunity from the initial epidemic disappears over time by waning immunity and population turnover. We also assume that there is no long-term environmental reservoir that would allow cholera to persist in Haiti.

5.4 Simulation of Vaccination Campaigns

We simulated rollout of vaccine by department. We assumed that vaccinated people were protected from infection but not against showing symptoms after getting infected ($VE_S > 0$ and $VE_P = 0$). We vaccinate approximately the same number of people each day from the start to the end date of the rollout. After vaccination is scheduled to start, a fixed amount of vaccine is added to the supply each day and cells (1kmx1km areas) within the desired department are chosen randomly for vaccination until the available vaccines are depleted. Small amounts of vaccine that are not used are carried forward to the next day. Cells are only vaccinated when there is sufficient vaccine to cover them at the desired level – those that are too large may be selected again later. Within the department of Ouest, we treat the communes of Port-au-Prince as a separate department for bookkeeping purposes. When we vaccinate Ouest, we first vaccinate the Port-au-Prince communes. We define "Port-au-Prince" to be the communes of Port-au-Prince, Delmas, Carrefour, and Pétion-Ville (about 2.6 million people). IHSI also includes the communes of Cite Soleil and Tabarre in Port-au-Prince, but they are not in the GADM shapefile we used.

We simulate four vaccination scenarios: full country in 2 years (scenario #1), two departments in 2 years (Centre and L'Artibonite) (scenario #2), full country in 5 years (scenario #3), and three departments in 2 years (Centre, L'Artibonite, and Ouest) (scenario #4). All rollouts begin on January 12, 2019, and departments are vaccinated in order of highest to lowest reported incidence in 2014 (Centre, L'Artibonite, Ouest, Nord-Ouest, Nord, Sud, Nippes, Nord-Est, Sud-Est, and Grand Anse). In these scenarios, 10,463, 2,348, 4,185, and 6,184 people are (fully) vaccinated per day, respectively, which covers 70% of the population. See Figure S32 for the number of individuals vaccinated in these four scenarios. The model does not accommodate "partially-vaccinated" people (i.e., everyone gets both doses of vaccine), so we do not consider 1-dose coverage in a vaccination campaign. Population-level vaccine coverage drops by nearly 2% per year because of births and deaths.

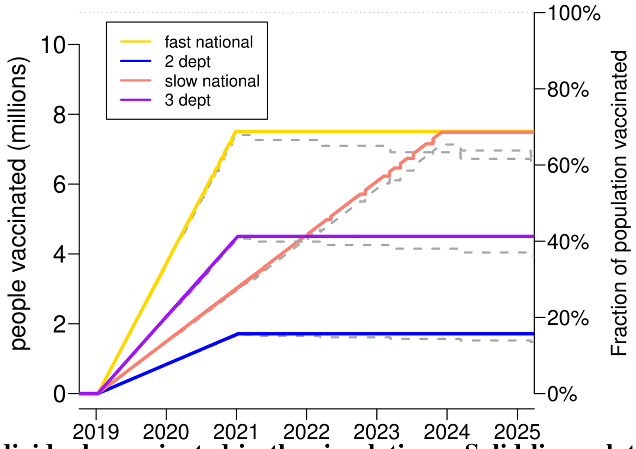

Figure S32: Number of individuals vaccinated in the simulations. Solid lines plot the cumulative number of people vaccinated for the four vaccination scenarios. Dashed gray lines indicate the number of living vaccinated people in the population, which declines as new individuals are born and others die. The left y-axis is scaled to the number of people vaccinated while the right y-axis is scaled to the fraction of Haiti's population that is vaccinated. All vaccinees receive both doses of OCV – no one is vaccinated with only one dose.

5.5 Calculating Elimination Measures

We define elimination to be the absence of any new cholera infections for one year. The time of elimination is the first day of a 365-day window with no new cholera infections (although there may be more infections one year after that), and the "time to elimination" is the number of days from January 12 2019 to the time of elimination. We found that in some runs, cholera spontaneously disappeared before vaccination started. We did not include these runs when computing the probability of elimination. We define the probability of elimination to be the fraction of runs have elimination among those that do *not* have elimination before the intervention starts. Resurgence is defined as seeing a new cholera infection after apparent elimination. We have an alternative definition of elimination that uses a threshold (T), where elimination is defined as when there are $< T$ (rather than < 1) new infections during a one-year window, and resurgence is defined as seeing T new cholera infections within any 365-day window after apparent elimination.

We did not observe a resurgence of cases after infections are not observed for one year (Figure S33). In theory, the aquatic reservoir could spark a new epidemic after such a long absence of cases but this is very unlikely because the amount of *Vibrio* in the reservoir decays exponentially and no external contamination or infected people are introduced. For the scenarios tested, once the number of infections drops below 1000 per year, resurgence was not observed (Figure S33). If there are 1000–9999 cases in a one-year window, resurgence could occur. When vaccine efficacy does not wane, elimination frequently occurs with the 2-year nationwide rollout with 70% coverage (Figure S34). In these runs, elimination generally occurs within three or four years of the start of vaccine rollout, so we would expect similar probability of elimination if the vaccine maintains high efficacy for 4 years.

Variation among runs for a single scenario comes from a combination of stochastic effects (different random number generator seeds) and the different random draws that determine rainfall after December 2018 and during the spring of 2013 with missing rainfall data.

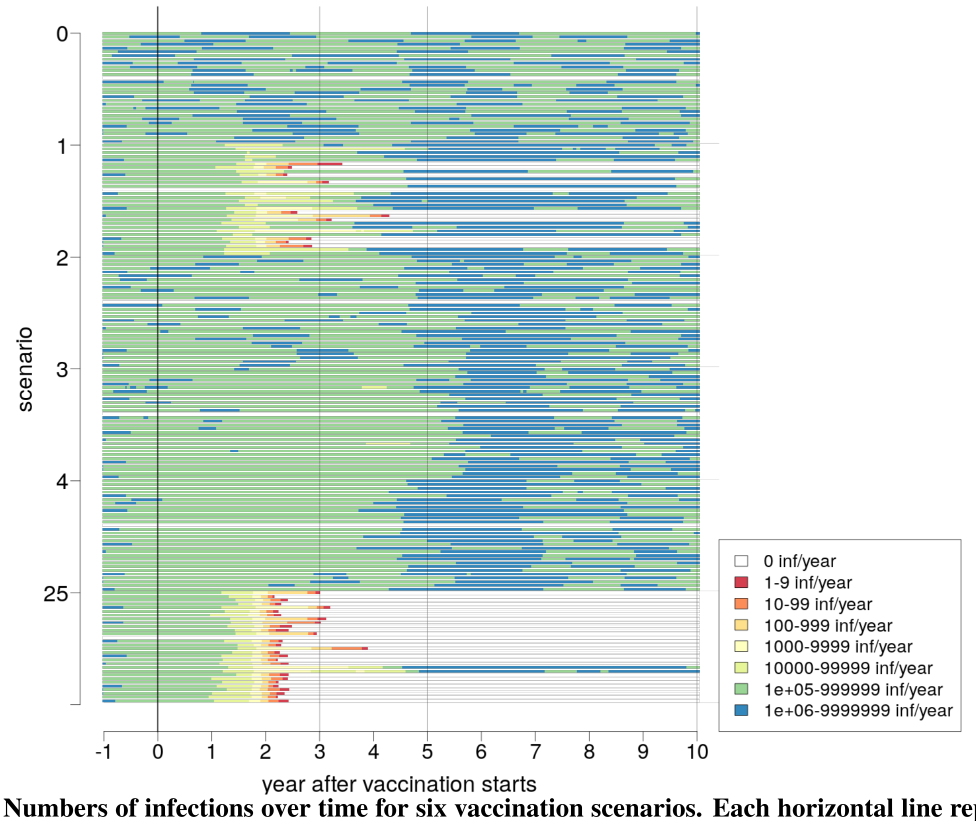

Figure S33: Numbers of infections over time for six vaccination scenarios. Each horizontal line represents the output from a single stochastic run. Six scenarios were chosen: 0) no vaccination, 1) vaccination of the whole country over 2 years (70% vaccine coverage), 2) vaccination of 2 departments over 2 years, 3) vaccination of the whole country over 5 years, 4) vaccination of 3 departments over 2 years, and 25) vaccination of the whole country over 2 years with high coverage (95% coverage). The runs for each scenario is depicted as a stack of 30 horizontal lines. For each day of the year, the number of cholera infections that occur over the next 365 days is indicated by color. For example, regions in red indicate days for which there are only 1 to 9 infections over the next 365 days in the model run, while green indicates 100,000 to 999,999 infections. White dots indicate that no new infections occur within 365 days, the strictest definition of "elimination".

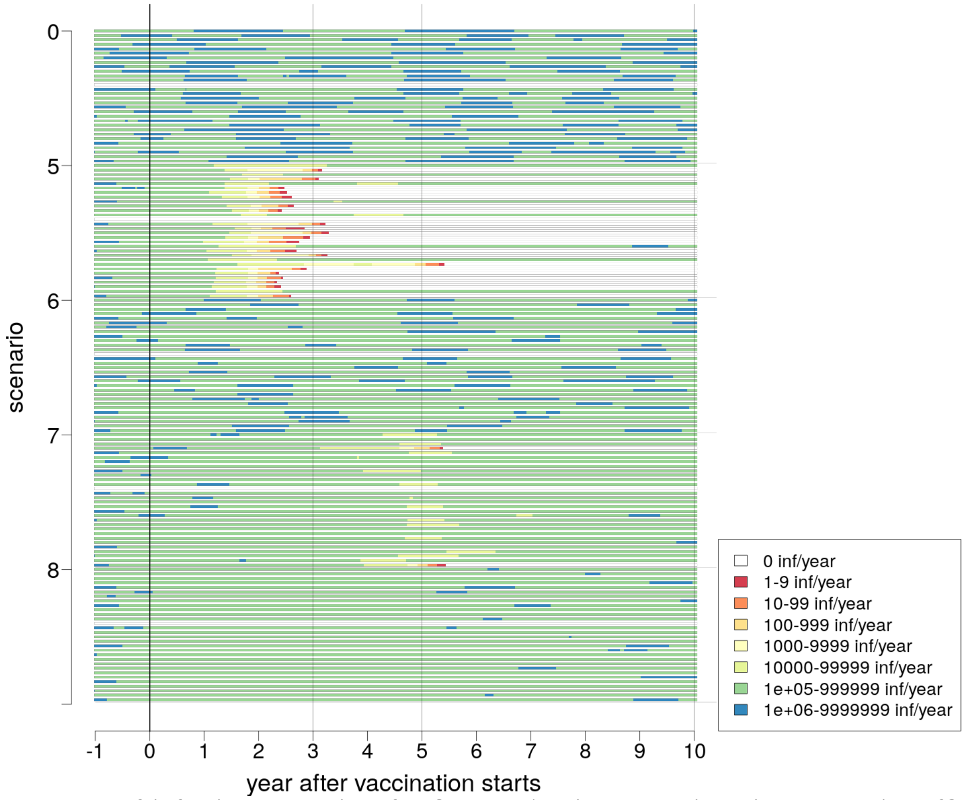

Figure S34: Numbers of infections over time for five vaccination scenarios with no vaccine efficacy waning. Each horizontal line represents the output from a single stochastic run. Five scenarios were chosen: 0) no vaccination, 5) vaccination of the whole country over 2 years, 6) vaccination of 2 departments over 2 years, 7) vaccination of the whole country over 5 years, and 8) vaccination of 3 departments over 2 years. The runs for each scenario is depicted as a stack of 30 horizontal lines. For each day of the year, the number of cholera infections that occur over the next 365 days is indicated by color. For example, regions in red indicate days for which there are only 1 to 9 infections over the next 365 days in the model run, while green indicates 100,000 to 999,999 infections. White dots indicate that no new infections occur within 365 days, the strictest definition of "elimination". In these scenarios, vaccine efficacy does not decline over time.

Bibliography

[1] Rinaldo A, Bertuzzo E, Mari L, Righetto L, Blokesch M, Gatto M, et al. Reassessment of the 2010–2011 Haiti Cholera Outbreak and Rainfall-Driven Multiseason Projections. Proceedings of the National Academy of Sciences. 2012 Apr;109(17):6602–6607.

[2] Chao DL, Halloran ME, Longini IM Jr. Vaccination strategies for epidemic cholera in Haiti with implications for the developing world. Proc Natl Acad Sci U S A. 2011 Apr 26;108(17):7081–5.

[3] Azman AS, Luquero FJ, Ciglenecki I, Grais RF, Sack A, Lessler J. The Impact of a One-Dose versus Two-Dose Oral Cholera Vaccine Regimen in Outbreak Settings: A Modeling Study. PLOS Medicine. 2015 aug;12(8):e1001867.

[4] Ministère de la Santé Publique et de la Population. Profil statistique Cholera;. http://mspp.gouv.ht/newsite/.

[5] Ministère de la Santé Publique et de la Population et Direction Nationale de l'Eau Potable et de L'Assainissement, République d'Haïti. Plan National D'Élimination Du Cholera, Developpement Du Moyen Terme, Juillet 2016 - Décembre 2018;.

[6] Bi Q, Ferreras E, Pezzoli L, Legros D, Ivers LC, Date K, et al. Protection against cholera from killed whole-cell oral cholera vaccines: a systematic review and meta-analysis. Lancet Infect Dis. 2017 Oct;17(10):1080–1088.

[7] Franke MF, Ternier R, Jerome JG, Matias WR, Harris JB, Ivers LC. Long-term effectiveness of one and two doses of a killed, bivalent, whole-cell oral cholera vaccine in Haiti: an extended case-control study. The Lancet Global Health. 2018;6(9):e1028–e1035. Available from: https://linkinghub.elsevier.com/retrieve/pii/S2214109X18302845.

[8] Bjørnstad ON, Finkenstadt BF, Grenfell BT. Dynamics of Measles Epidemics: Estimating Scaling of Transmission Rates Using a Time Series SIR Model. Ecological Monographs. 2002;72(2):169–184.

[9] Grenfell BT, Bjornstad ON, Finkenstadt BF. Dynamics of Measles Epidemics: Scaling Noise, Determinism, and Predictability with the TSIR Model. Ecological Monographs. 2002;72(2):185–202.

[10] Lewnard JA, Antillón M, Gonsalves G, Miller AM, Ko AI, Pitzer VE. Strategies to Prevent Cholera Introduction during International Personnel Deployments: A Computational Modeling Analysis Based on the 2010 Haiti Outbreak. PLoS Medicine. 2016;13(1):1–23.

[11] United Nations DESA, Population Division. World Population Prospects 2019, Haiti;. Available from: https://population.un.org/wpp/.

[12] King AA, Nguyen D, Ionides EL. Statistical Inference for Partially Observed Markov Processes via the R Package pomp. Journal of Statistical Software. 2016;69(12):1–43.

[13] King AA, Ionides EL, Bretó CM, Ellner SP, Ferrari MJ, Kendall BE, et al.. pomp: Statistical Inference for Partially Observed Markov Processes; 2018. R package, version 1.19. Available from: https://kingaa.github.io/pomp/.

[14] Ionides EL, Nguyen D, Atchadé Y, Stoev S, King AA. Inference for dynamic and latent variable models via iterated, perturbed Bayes maps. Proceedings of the National Academy of Sciences of the United States of America. 2015;112(3):719–24. Available from: http://www.pnas.org/content/112/3/719.

[15] Institute Haïtien de Statistique et d'Informatique. Estimation de la population de 2015;. Available from: http://www.rgph-haiti.ht/haiti-en-bref/.

[16] Central Intelligence Agency. The World Factbook 2016–17; 2016. Available from: `https://www.cia.gov/library/publications/the-world-factbook/index.html`.

[17] Azman AS, Rudolph KE, Cummings DAT, Lessler J. The Incubation Period of Cholera: A Systematic Review. Journal of Infection. 2013 May;66(5):432–438.

[18] Weil AA, Khan AI, Chowdhury F, Larocque RC, Faruque ASG, Ryan ET, et al. Clinical outcomes in household contacts of patients with cholera in Bangladesh. Clin Infect Dis. 2009 Nov 15;49(10):1473–9.

[19] Levine MM, Black RE, Clements ML, Cisneros L, Nalin DR, Young CR. Duration of Infection-Derived Immunity to Cholera. The Journal of Infectious Diseases. 1981 Jun;143(6):818–820.

[20] Koelle K, Pascual M. Disentangling Extrinsic from Intrinsic Factors in Disease Dynamics: A Nonlinear Time Series Approach with an Application to Cholera. The American Naturalist. 2004;163(6):901–913.

[21] Halloran ME, Struchiner CJ, Longini IM. Study designs for evaluating different efficacy and effectiveness aspects of vaccines. American Journal of Epidemiology. 1997;146(10):789–803.

[22] Berman L. "Haiti Earthquake Data (VECTORS)". Harvard Dataverse; 2015. Available from: `{https://doi.org/10.7910/DVN/BAGUVN}`.

[23] DIVA-GIS. Haiti Elevation Grid File;. Available from: `{http://www.diva-gis.org/datadown}` [cited Nov, 2016].

[24] Lewer JJ, Van den Berg H. A gravity model of immigration. Economics Letters. 2008;99(1):164 – 167. Available from: `http://www.sciencedirect.com/science/article/pii/S0165176507002455`.

[25] Fung ICH. Cholera transmission dynamic models for public health practitioners. Emerg Themes Epidemiol. 2014 Feb;11(1):1.

[26] Jackson BR, Talkington DF, Pruckler JM, Fouche MDB, Lafosse E, Nygren B, et al. Seroepidemiologic survey of epidemic cholera in Haiti to assess spectrum of illness and risk factors for severe disease. Am J Trop Med Hyg. 2013 Oct;89(4):654–664.

[27] WHO. Prevention and control of cholera outbreaks: WHO policy and recommendations; 2010. `https://www.who.int/cholera/technical/prevention/control/en/`.

[28] Nelson EJ, Harris JB, Morris JG Jr, Calderwood SB, Camilli A. Cholera transmission: the host, pathogen and bacteriophage dynamic. Nat Rev Microbiol. 2009 Oct;7(10):693–702.

[29] WHO. The global burden of cholera; 2012. https://www.who.int/bulletin/volumes/90/3/11-093427/en/.

[30] Virtanen P, Gommers R, Oliphant TE, Haberland M, Reddy T, Cournapeau D, et al. SciPy 1.0–Fundamental Algorithms for Scientific Computing in Python. arXiv e-prints. 2019 Jul;p. arXiv:1907.10121.

[31] WHO. Haiti's Ministry of Health successfully vaccinates 729,000 persons against cholera; 2016. `https://www.paho.org/hq/index.php?option=com_content&view=article&id=12771:haiti-ministry-of-health-vaccinates-729000-persons-against-cholera&Itemid=135&lang=en`.

[32] Bertuzzo, Mari L , Righetto L , Gatto M , Casagrandi R , Blokesch M , et al. Prediction of the Spatial Evolution and Effects of Control Measures for the Unfolding Haiti Cholera Outbreak. Geophysical Research Letters. 2011 Mar;38(6).

[33] Bertuzzo E, Finger F, Mari L, Gatto M, Rinaldo A. On the Probability of Extinction of the Haiti Cholera Epidemic. Stochastic Environmental Research and Risk Assessment. 2016 Dec;30(8):2043–2055.

[34] Pasetto D, Finger F, Camacho A, Grandesso F, Cohuet S, Lemaitre JC, et al. Near Real-Time Forecasting for Cholera Decision Making in Haiti after Hurricane Matthew. PLOS Computational Biology. 16-May-2018;14(5):e1006127.

[35] Lemaitre J, Pasetto D, Perez-Saez J, Sciarra C, Wamala JF, Rinaldo A. Rainfall as a Driver of Epidemic Cholera: Comparative Model Assessments of the Effect of Intra-Seasonal Precipitation Events. Acta Tropica. 2019 Feb;190:235–243.

[36] King AA, Ionides EL, Pascual M, Bouma MJ. Inapparent Infections and Cholera Dynamics. Nature. 2008 Aug;454(7206):877.

[37] Camacho A, Bouhenia M, Alyusfi R, Alkohlani A, Naji MAM, de Radiguès X, et al. Cholera Epidemic in Yemen, 2016–18: An Analysis of Surveillance Data. The Lancet Global Health. 2018 Jun;6(6):e680–e690.

[38] Kühn J, Finger F, Bertuzzo E, Borgeaud S, Gatto M, Rinaldo A, et al. Glucose- but Not Rice-Based Oral Rehydration Therapy Enhances the Production of Virulence Determinants in the Human Pathogen Vibrio Cholerae. PLOS Neglected Tropical Diseases. 2014;8(12):e3347.

[39] Kaper JB, Morris JG, Levine MM. Cholera. Clinical Microbiology Reviews. 1995 Jan;8(1):48–86.

[40] Codeço CT. Endemic and Epidemic Dynamics of Cholera: The Role of the Aquatic Reservoir. BMC Infectious Diseases. 2001 Feb;1:1.

[41] Bretó C, Ionides EL. Compound Markov Counting Processes and Their Applications to Modeling Infinitesimally Over-Dispersed Systems. Stochastic Processes and their Applications. 2011 Nov;121(11):2571–2591.

[42] Bretó C, He D, Ionides EL, King AA. Time Series Analysis via Mechanistic Models. The Annals of Applied Statistics. 2009 Mar;3(1):319–348.

[43] Bertuzzo E, Azaele S, Maritan A, Gatto M, Rodriguez-Iturbe I, Rinaldo A. On the Space-Time Evolution of a Cholera Epidemic. Water Resources Research. 2008;44(1).

[44] Huffman GJ, Adler RF, Bolvin DT, Gu G, Nelkin EJ, Bowman KP, et al. The TRMM Multisatellite Precipitation Analysis (TMPA): Quasi-Global, Multiyear, Combined-Sensor Precipitation Estimates at Fine Scales. Journal of Hydrometeorology. 2007;8(1):38–55.

[45] Ionides EL, Nguyen D, Atchadé Y, Stoev S, King AA. Inference for Dynamic and Latent Variable Models via Iterated, Perturbed Bayes Maps. Proceedings of the National Academy of Sciences of the United States of America. 2015 Jan;112(3):719–724.

[46] Park J, Ionides EL. A guided intermediate resampling particle filter for inference on high dimensional systems. arXiv preprint arXiv:170808543. 2017;.

[47] Rebaudet S, Bulit G, Gaudart J, Michel E, Gazin P, Evers C, et al. The National Alert-Response Strategy against Cholera in Haiti: A Four-Year Assessment of Its Implementation. bioRxiv. 2018 Feb;p. 259366.

[48] Minnesota Population Center. Integrated Public Use Microdata Series, International; 2019. Available at `https://international.ipums.org/international/`.

[49] Sorichetta A, Hornby GM, Stevens FR, Gaughan AE, Linard C, Tatem AJ. High-resolution gridded population datasets for Latin America and the Caribbean in 2010, 2015, and 2020. Sci Data. 2015;2:150045.

[50] Hartley DM, Morris JG Jr, Smith DL. Hyperinfectivity: a critical element in the ability of *V. cholerae* to cause epidemics. PLoS Med. 2006 Jan;3(1):e7.

[51] OpenStreetMap contributors. Planet dump retrieved from https://planet.osm.org ; 2019. `https://www.openstreetmap.org`.

[52] Rebaudet S, Gazin P, Barrais R, Moore S, Rossignol E, Barthelemy N, et al. The dry season in Haiti: a window of opportunity to eliminate cholera. PLoS Curr. 2013;5.

[53] Chao DL, Halstead SB, Halloran ME, Longini IM Jr. Controlling dengue with vaccines in Thailand. PLoS Negl Trop Dis. 2012 Oct;6(10):e1876. Available from: `http://dx.doi.org/10.1371\%2Fjournal.pntd.0001876`.

[54] Fong Y, Halloran ME, Park JK, Marks F, Clemens JD, Chao DL. Efficacy of a bivalent killed whole-cell cholera vaccine over five years: a re-analysis of a cluster-randomized trial. BMC Infect Dis. 2018 Feb 20;18(1):84.

[55] Sack DA, Sack RB, Nair GB, Siddique AK. Cholera. Lancet. 2004 Jan 17;363(9404):223–33.

[56] Phelps M, Perner ML, Pitzer VE, Andreasen V, Jensen PKM, Simonsen L. Cholera Epidemics of the Past Offer New Insights Into an Old Enemy. J Infect Dis. 2018 Jan 30;217(4):641–649.

[57] Andrews JR, Basu S. Transmission dynamics and control of cholera in Haiti: an epidemic model. The Lancet. 2011;377(9773):1248–1255. Available from: `http://www.sciencedirect.com/science/article/pii/S0140673611602730`.

[58] Hulland E, Subaiya S, Pierre K, Barthelemy N, Pierre JS, Dismer A, et al. Increase in Reported Cholera Cases in Haiti Following Hurricane Matthew: An Interrupted Time Series Model. Am J Trop Med Hyg. 2019 Feb;100(2):368–373.
